# Accelerating the development of a psychological intervention to restore treatment decision-making capacity in patients with schizophrenia-spectrum disorder: a study protocol for a multi-site, assessor-blinded, pilot Umbrella trial (the DEC:IDES trial)

**DOI:** 10.1186/s40814-023-01323-0

**Published:** 2023-07-08

**Authors:** Paul Hutton, James Kelly, Christopher D. J. Taylor, Brian Williams, Richard Emsley, Candy Ho Alexander, Anvita Vikram, David Saddington, Andrea McCann, Joseph Burke, Emma Eliasson, Sean Harper, Thanos Karatzias, Peter J. Taylor, Andrew Watson, Nadine Dougall, Jill Stavert, Suzanne O’Rourke, Angela Glasgow, Regina Murphy, Karen Palmer, Nosheen Zaidi, Polly Bidwell, Jemma Pritchard, Lucy Carr, Amanda Woodrow

**Affiliations:** 1 School of Health & Social Care, Edinburgh Napier University, Edinburgh, UK; 2 Edinburgh Research & Innovation Centre for Complex and Acute Mental Health Problems, Edinburgh, UK; 3 Faculty of Health & Medicine, Lancaster University, Lancaster, UK; 4 Lancashire & South Cumbria NHS Foundation Trust, Preston, UK; 5 Pennine Care NHS Foundation Trust, Ashton-Under-Lyne, UK; 6 Division of Psychology & Mental Health, Manchester Academic Health Sciences Centre, University of Manchester, Manchester, UK; 7 School of Health, Social Care & Life Sciences, University of the Highlands and Islands, Inverness, UK; 8 Institute of Psychiatry, Psychology & Neuroscience, Kings College London, London, UK; 9 NHS Lothian, Edinburgh, UK; 10 NHS Research Scotland Mental Health Network, Edinburgh, UK; 11 National Centre for Suicide Research and Prevention, Karolinska Institutet, Stockholm, Sweden; 12 School of Health in Social Science, University of Edinburgh, Edinburgh, UK

**Keywords:** Schizophrenia, CBT, MCT, Decision-making capacity, Patient autonomy, Umbrella trial, Supported decision-making, Randomised controlled trial

## Abstract

**Background:**

A high proportion of patients diagnosed with schizophrenia-spectrum disorders will at some point in their lives be assessed as not having the capacity to make their own decisions about pharmacological treatment or inpatient care (‘capacity’). Few will be helped to regain it before these interventions proceed. This is partly because effective and safe methods to do so are lacking. Our aim is to accelerate their development by testing, for the first time in mental healthcare, the feasibility, acceptability and safety of running an ‘Umbrella’ trial. This involves running, concurrently and under one multi-site infrastructure, multiple assessor-blind randomised controlled trials, each of which is designed to examine the effect on capacity of improving a single psychological mechanism (‘mechanism’). Our primary objectives are to demonstrate feasibility of (i) recruitment and (ii) data retention on the MacArthur Competence Assessment Tool-Treatment (MacCAT-T; planned primary outcome for a future trial) at end-of-treatment. We selected three mechanisms to test: ‘self-stigma’, low self-esteem and the ‘jumping to conclusions’ bias. Each is highly prevalent in psychosis, responsive to psychological intervention, and hypothesised to contribute to impaired capacity.

**Methods:**

Sixty participants with schizophrenia-spectrum diagnoses, impaired capacity and one or more mechanism(s) will be recruited from outpatient and inpatient mental health services in three UK sites (Lothian, Scotland; Lancashire and Pennine; North West England). Those lacking capacity to consent to research could take part if the key criteria were met, including either proxy consent (Scotland) or favourable Consultee advice (England). They will be allocated to one of three randomised controlled trials, depending on which mechanism(s) they have. They will then be randomised to receive, over an 8-week period and in addition to treatment as usual (TAU), 6 sessions of either a psychological intervention which targets the mechanism, or 6 sessions of assessment of the causes of their incapacity (control condition). Participants are assessed at 0 (baseline), 8 (end-of-treatment) and 24 (follow-up) weeks post-randomisation using measures of capacity (MacCAT-T), mechanism, adverse events, psychotic symptoms, subjective recovery, quality of life, service use, anxiety, core schemata and depression. Two nested qualitative studies will be conducted; one to understand participant and clinician experiences and one to investigate the validity of MacCAT-T appreciation ratings.

**Discussion:**

This will be the first Umbrella trial in mental healthcare. It will produce the first 3 single-blind randomised controlled trials of psychological interventions to support treatment decision-making in schizophrenia-spectrum disorder. Demonstrating feasibility will have significant implications not only for those seeking to support capacity in psychosis, but also for those who wish to accelerate the development of psychological interventions for other conditions.

**Trial registration:**

ClinicalTrials.gov NCT04309435. Pre-registered on 16 March 2020.

**Supplementary Information:**

The online version contains supplementary material available at 10.1186/s40814-023-01323-0.

## Background

Approximately 9–10 people per 1000 will be diagnosed with a schizophrenia-spectrum disorder (‘psychosis’) at some point in their lives [1]. Those affected may experience a range of distressing symptoms, from hallucinations, delusions and conceptual disorganisation to reduced motivation, anhedonia and cognitive impairment. They have a much greater risk of dying by suicide relative to the general population [2] which, together with significantly poorer physical health, contributes to a reduction in their life expectancy of 14–15 years [3].

People with psychosis are also very likely to experience both ‘external’ threats to their autonomy, in the form of involuntary psychiatric treatment, and ‘internal’ threats, arising from the effects of psychotic symptoms on their ability to make decisions [4, 5]. In turn, clinicians must balance their duty to provide them with effective care and treatment with their duty to promote their autonomy. Although traditional treatments for psychosis may have beneficial effects on autonomy over the medium to longer-term [6], these must be weighed against the immediate loss of autonomy that occurs if they are administered under compulsion, particularly if they interfere with one’s bodily integrity, liberty or right to a private life [7, 8]. In many jurisdictions, it is therefore necessary—but not sufficient—to first demonstrate that a person lacks the ability or ‘mental capacity’ to make decisions about that treatment, before it can proceed without their consent [9, 10].

Definitions of mental capacity vary; however, most involve the ability to understand decision-relevant information and the ability to communicate one’s decision. Many also involve the abilities to retain, use and weigh relevant information and/or appreciate it [11]. When the capacity to make treatment decisions is lost (hereafter ‘capacity’), there is a long-standing ethical, legal and human-rights based imperative for clinicians to support its return [12–15]. In practice, however, such support is rare [16, 17].

There is increasing pressure for this to change. The United Nations Committee on the Convention on the Rights of Persons with Disabilities (UNCRPD), the National Institute of Clinical and Care Excellence (NICE) and the recent Wessely and Scott Reviews of UK mental health legislation have all emphasised the fundamental importance of supporting treatment decision-making to protect a person’s autonomy [18–21]. Despite these developments, there is a lack of evidence—across healthcare—on how to do this effectively [19].

For people with psychosis, our recent systematic review confirmed there are no evidence-based interventions to restore their ability to make their own treatment decisions [5]. We have conducted a number of studies to address this [4, 22–29] which, taken together, suggest a lack of capacity in this group may stem from specific cognitive, emotional and social factors, the independent and interacting effects of which are moderated by awareness of them. We specifically predict that avoiding exposure to self-stigmatising beliefs about illness may motivate a person to reject the possibility they have any need for care, that low self-esteem may fuel distrust and treatment-related paranoia and that individuals with a ‘jumping to conclusions’ bias may struggle to gather sufficient information about treatment before accepting or rejecting it. How these factors interact in those lacking capacity is not yet known, but it is plausible to suggest, for instance, that low self-esteem and high self-stigma may reinforce each other [30, 31] or that a person with low self-esteem and the JTC bias may be particularly vulnerable to developing self-stigma.

Self-stigma, low self-esteem and the jumping to conclusions bias are each highly prevalent in psychosis [24, 29, 32], and psychological interventions that selectively reduce them already exist [22, 33–38]. This means we can conduct ‘interventionist-causal RCTs’ (IC-RCTs) to examine whether they also improve capacity [39, 40]. Participants in an IC-RCT are selected to ensure they have both the condition (e.g., impaired capacity) and the hypothesised cause of the condition (e.g., jumping to conclusions bias), before being randomly allocated to either a control condition or an intervention designed to reduce the potential cause [40]. Positive results mean both a cause and a treatment component have been identified, whereas null results are informative insofar as they allow model refinement.

However, because complex conditions have multiple causes, multiple IC-RCTs are required to develop a comprehensive intervention, which can be expensive and delay treatment development. One solution is to run several of these trials at the same time within one overall infrastructure, with each focused on a different cause. This removes duplication of time and effort in relation to protocol development, ethical approval, advertising, recruitment, management, staff training, so on, therefore greatly increasing efficiency. Indeed, we estimate that this approach, which is also known as an ‘Umbrella trial’, could produce an effective intervention in half the time, for half the cost. However, although they have been highly successful in improving treatments for cancer and other physical health problems [41, 42], Umbrella trials have never before been used to develop a psychological or pharmacological intervention for a mental health problem [42, 43].

Our aim is therefore to conduct the first Umbrella trial in mental healthcare, using this to accelerate the development of the first evidence-based intervention to support capacity in psychosis. The DEC:IDES (‘DEcision-making Capacity: Intervention Development and Evaluation in Schizophrenia-spectrum disorders’) trial involves running three IC-RCTs in parallel, each testing the effect on capacity of an intervention to either reduce self-stigma, improve self-esteem or reduce the jumping to conclusions bias. Our aims at this stage are restricted to demonstrating feasibility, acceptability and safety. Our primary objectives are to demonstrate feasibility of recruitment and determine data quality and completion rates for the MacArthur Competence Assessment Tool for Treatment (MacCAT-T) [44], a widely used measure of treatment decision-making capacity and our planned primary outcome in a future trial. Our secondary objectives include assessing adverse events, data completion rates for secondary efficacy and mechanism outcomes, participant and clinician acceptability of the trial, and the construct validity of the MacCAT-T.

## Methods

### Design

**Fig. 1:**
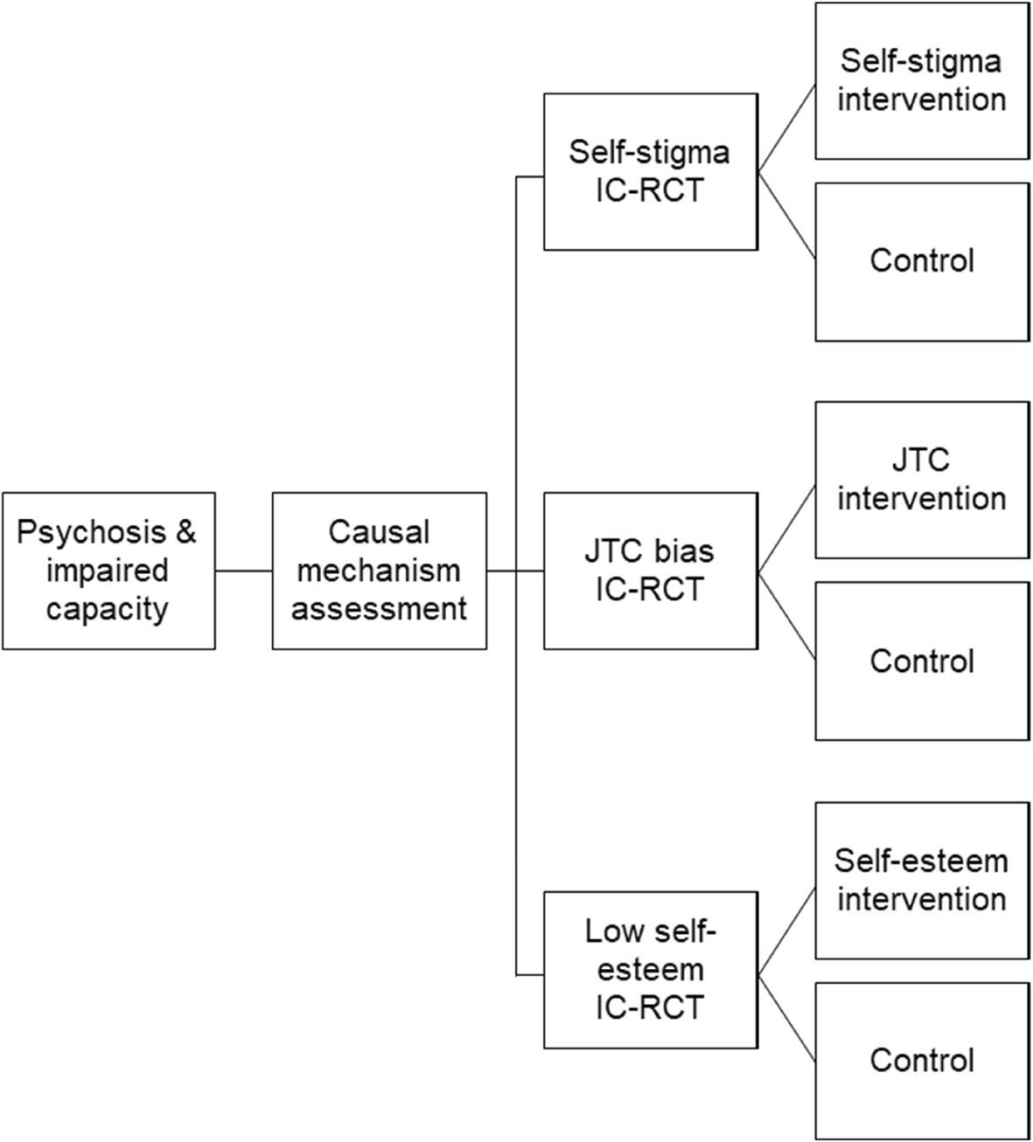
An Umbrella trial of psychological intervention to improve impaired treatment decision-making capacity in psychosis

DEC:IDES is a multi-site single (rater) blind Umbrella trial of psychological interventions to support treatment decision-making capacity in people diagnosed with schizophrenia-spectrum disorders. Participants are randomly allocated to receive treatment as usual (TAU) plus a psychological intervention to improve either (i) self-stigma, (ii) self-esteem or (iii) the jumping to conclusions (JTC) reasoning bias, or TAU plus an attention control condition (see Fig. 1). Each intervention group is compared to its own control group (each receive the same standardised procedure) to ensure participants in each trial are equivalent with respect to their presenting mechanism. This means the study consists of three 2-arm IC-RCTs (treatment vs. control) running in parallel under one overall infrastructure, or ‘Umbrella’. TAU is measured, but not changed.

We provide control participants with a ‘non-specific’ attention control condition (in addition to TAU) because we need to demonstrate acceptability before using it in a larger trial. An appropriate attention-control reduces the risk of a false-positive finding favouring intervention efficacy (i.e., it increases confidence that group differences reflect specific causal effects of the intervention). Our control condition involves a therapist completing further assessment of factors which help or hinder a participant’s capacity and is carefully matched for time and attention to the interventions.

Assessments are carried out at 0 (baseline), 8 (end-of-treatment; EoT) and 24 weeks (follow-up; FU) by a researcher masked to treatment allocation. Due to limited resources and because our intention at this stage is simply to demonstrate the feasibility of retaining participants for follow-up, only those randomised in the first 5 (England) to 23 (Scotland) months are eligible for the 24-week assessment.^1^ We have multiple sites (1 in Scotland; 2 in England) primarily to examine the feasibility of multi-site procedures. To minimise cost, we made an a priori decision to recruit 75% of participants from the lead site, NHS Lothian.

There are two nested qualitative studies; one uses framework analysis [45] to document and understand participant and clinician experiences of the trial (‘Qualitative study 1’), and another uses case study methodology [46, 47] to investigate the validity of improvements in MacCAT-T appreciation ratings (‘Qualitative study 2’). Both these studies are conducted after participants have completed their final research assessment.

DEC:IDES was approved by two NHS Research Ethics Committees (RECs) representing Scotland (IRAS ID: 263575) and England (IRAS ID: 265638). The NHS Health Research Authority required separate approval from both Scotland and England because of differences in legal regimes governing the inclusion of adults who lack capacity to consent to research. DEC:IDES was pre-registered on 16 March 2020, prior to first randomisation,

(NCT04309435; https://clinicaltrials.gov/ct2/show/NCT04309435).^2^

Informed consent is acquired from all participants with intact decision-making capacity to consent to research, using an approved consent form.^3^ Special REC approval was obtained to include participants who lacked capacity to consent to participate, based on the study and its procedures meeting criteria specified in the Mental Capacity (England & Wales) Act (2005) and Adults with Incapacity (Scotland) Act (2001) [48, 49].

A Trial Steering Committee (TSC) provides oversight of the trial and incorporates the functions of a Data Monitoring and Ethics Committee (DMEC). Membership includes service-user and carer representatives, researchers and clinicians. The TSC reviews all SAEs as they occur and can recommend to the Sponsor and NHS REC that the trial be terminated for reasons of safety.

A schedule of enrolment, interventions and assessments is provided in Table 1. See Additional file 1 for a combined checklist incorporating the ‘Standard Protocol Items: Recommendations for Interventional Trials’ (SPIRIT) checklist and the CONSERVE extension to SPIRIT for reporting changes due to extenuating circumstances, together with relevant items from the ‘Consolidated Standards of Reporting Trials’ (CONSORT) extensions for pilot and feasibility trials [50], for harms [51] and for social and psychological interventions [52]. Figure 3 provides the CONSORT participant flow diagram template, designed to provide more detailed information on recruitment flow [50].

**Table 1:** Schedule of enrolment, interventions and assessments (SPIRIT 2013 guidelines)

		**Study period**			
**Time point**	**Enrolment**	**Allocation**	**Post-allocation**		
	*-t*_*1*_	*0 weeks*	*8 weeks (end-of-treatment)*	*24 weeks (follow-up)*	> *24 weeks*
**Enrolment**					
*Eligibility screen*	X				
*Informed consent*	X				
*Allocation*		X			
**Interventions**					
*Self-esteem*			X		
*Self-stigma*			X		
*JTC*			X		
*Control*			X		
**Assessments**					
*Planned primary outcome*		X	X	X	
*Planned secondary outcomes*		X	X	X	
*Planned mechanisms*		X	X	X	
**Qualitative studies**					
*Study 1*					X
*Study 2*					X

### Participants

We calculated that 60 participants (20 per trial) would allow us to estimate a data non-retention rate of 15%, at week 8, to within a 95% confidence interval of ± 9%.

Individuals can participate in DEC:IDES if they are as follows:

- Diagnosed with schizophrenia-spectrum disorder (schizophrenia, schizoaffective disorder, delusional disorder, psychosis not otherwise specified, brief psychotic disorder)
- Aged 18–65
- Able to be interviewed and complete the measures
- Registered as a patient with clinical or social care services
- Judged to lack capacity to make treatment decisions by their referring clinician and the researcher (using the MacCAT-T)
- Have either (i) low self-esteem, defined as a score of < 15 on the Rosenberg Self-Esteem Scale (RSES) [53]; (ii) high self-stigma, defined as a score of ≥ 60 on Internalised Stigma of Mental Illness Inventory (ISMI) [54]; and/or (iii) a JTC bias, defined as selecting ≤ 2 beads on the Beads Task [29, 55].

Individuals are unable to participate if they are as follows:

- Have a moderate to severe learning disability
- Have psychosis of a predominantly organic origin (e.g. brain injury, physical health condition, epilepsy) or a primary diagnosis of substance or alcohol use disorder
- Cannot understand English sufficiently to engage in conversation without an interpreter
- Present with a level of risk to others that cannot be managed via suitable adjustments.

**Fig. 2:**
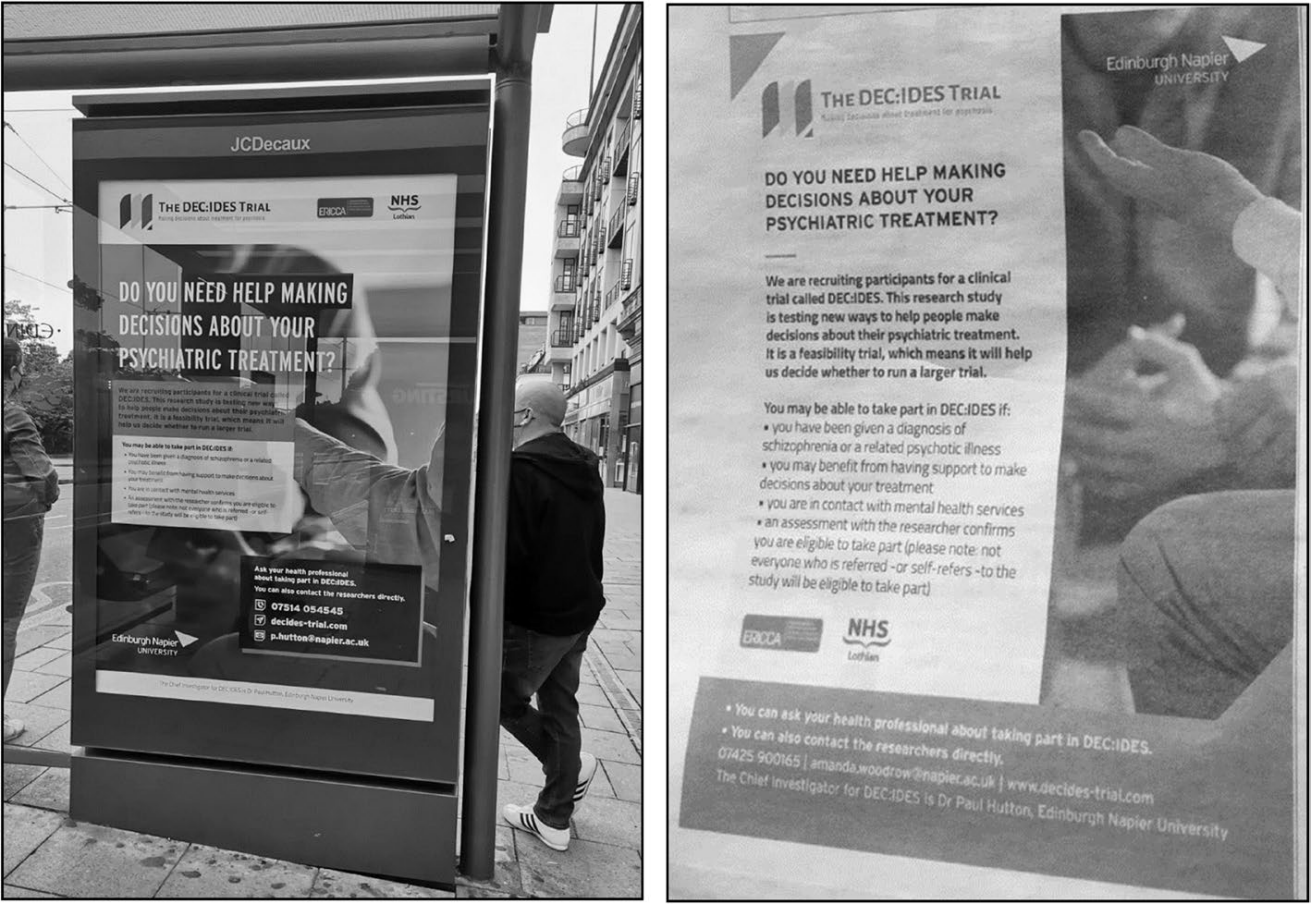
Newspaper and bus shelter recruitment adverts in Lothian site

**Fig. 3:**
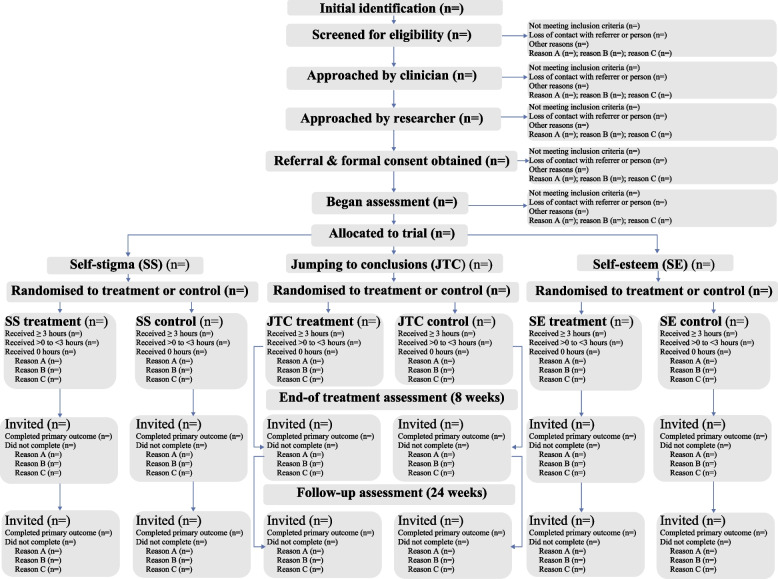
CONSORT diagram template

Research assistants (RAs) seek referrals from clinicians in inpatient and outpatient clinical services in NHS Lothian, Lancashire & South Cumbria NHS Foundation Trust and Pennine Care NHS Foundation Trust. We also accept self-referral, but only if the participant agrees to us contacting their mental health provider for risk assessment purposes. Posters are placed in inpatient wards and outpatient clinics to advertise the study to participants. These provide the study website address, which hosts information sheets and the consent form. Adverts were also placed in newspapers and bus stop shelters in the NHS Lothian area (see Fig. 2). Interested participants are provided with an information sheet, and any initial questions are answered. They are then recontacted by an RA a minimum of 48 h later. Those eligible and consenting then enter the trial. To compensate them for their time, participants received a £10 supermarket voucher after the baseline assessment, and again after the end-of-treatment and follow-up assessments (£30 in total). Reasons for exclusion will be reported as per the CONSORT 2010 guidelines [56] and the 2016 extension for feasibility and pilot trials [50]. Figure 3 provides the CONSORT participant flow diagram template, designed to provide more detailed information on recruitment flow.

#### Qualitative study 1

We aim to recruit 6 patients and 6 staff members from the Lothian site for this study, via a mixture of purposive and random sampling. We try to ensure there is one patient from every arm of the study, that half are from an inpatient setting and that one third are self-referrers. A similar process is applied to selecting the staff sample. When we have multiple patients or staff to choose from, we use an online randomisation website (https://www.random.org/lists/) to determine order of invitation.

#### Qualitative study 2

We aim to recruit up to 10 patients from the Lothian site for this study. Participants are eligible if they have completed the main study in the preceding 6 months and if their scores for appreciation on the MacCAT-T have improved by at least 1 point between assessments. If there are more than 10 eligible participants, the order of invitation will again be determined randomly.

### Randomisation and blinding

There are two points of randomisation: randomisation to trial (‘R1’) and randomisation to treatment or control (‘R2’). R1 is used to allocate individuals when they are eligible for two or more trials. Randomisation is conducted by the automated and online service provided by Sealed Envelope (https://www.sealedenvelope.com/) using concealed and randomly generated allocation sequences (without stratification or random permuted blocks) per each possible combination of trials, with 1:1 or 1:1:1 ratios.^4^ This sequence was generated and stored online by Sealed Envelope and was inaccessible to the research team.

R2 is also performed by Sealed Envelope, using a single concealed and randomly generated allocation sequence (1:1 ratio), stratified by trial and using random permuted blocks of 2 and 4. There was no stratification by therapist or site. PJT used Sealed Envelope to generate this sequence, which was inaccessible to the rest of the research team. PJT was masked to participant allocation and played no role in their enrolment or assessment. In order to minimise non-ignorable missing data, R2 is performed as late as possible, which is normally the beginning of the participant’s first meeting with their therapist.^5^ The result of R2 is communicated by email to the Chief Investigator, who informs the therapist by phone, who in turn informs the participant.

Outcome assessors are masked to the result of R2 but not R1 (no comparisons between trials are planned). Masking is maintained by (i) assessors and therapists having separate offices, phone numbers and filing systems; (ii) assessors reminding participants at the start of any phone calls or in-person meetings to not disclose their allocation; and (iii) assessors not examining clinical notes after R2 are performed. Assessors and therapists also refrain from discussing participants after R2, unless required to manage risk. If an unmasking occurs, this and its cause are recorded, and a new masked assessor completes any subsequent assessments. Deliberate unmasking was only allowed if doing so was judged by the CI and PI to be required to prevent harm occurring to the participant, a researcher or a third party.

### Assessments

Table 2 provides specific information on the purpose and timing of all measurements and outcomes.

**Table 2:** Timing and purpose of assessments

**Assessment**	**Time point**					**Purpose**					
	*0 weeks*	*8 weeks*	*24 weeks*	*Every therapy session*	*If early withdrawal*	*Describe sample*	*Determine eligibility*	*Feasibility of assessing efficacy*	*Feasibility of assessing change in mechanisms*	*Assess adverse events*	*MacCAT-T validation*
MacCAT-T	X	X	X			X	X	X			X
CIPD	X					X	X				
ISMI	X					X	X				
RSES	X	X	X			X	X		X		
Beads Task	X	X	X			X	X		X		
SIMS	X	X	X			X			X		
Demographics interview^a^	X					X					
BNA	X					X					
PANSS	X	X	X			X		X			
QPR	X	X	X			X		X			
SQoL	X	X	X			X		X			
CSRI	X	X	X			X		X			
BAI	X	X	X			X		X			
BCSS	X	X	X			X		X			
CDSS	X	X	X			X		X			
CDSS item 8 (suicidality)	X	X	X	X		X				X	
AEP—trial completers		X	X							X	
AEP—withdrawal					X					X	
CGI-P	X	X	X	X		X				X	
CGI-R	X	X	X	X		X				X	
SAI	X	X	X			X					X
CGI-C	X	X	X			X					X

*Note*: *MacCAT-T* MacArthur Competence Assessment Tool-Treatment; *CIPD* Clinical Interview for Psychotic Disorders; *ISMI* Internalised Stigma of Mental Illness Inventory; *RSES* Rosenberg Self Esteem Scale; *SIMS* Semi-structured Interview Measure of Stigma; *BNA* Brief Neurocognitive Assessment; *PANSS* Positive and Negative Syndrome Scale; *QPR* Questionnaire about the Process of Recovery; *SQoL* Schizophrenia Quality of Life Scale; *CSRI* Client Service Receipt Inventory; *BAI* Beck Anxiety Inventory; *BCSS* Brief Core Schema Scale; *CDSS* Calgary Depression Scale; *AEP* Adverse Events in Psychotherapy questionnaire; *CGI-P* Clinical Global Impression – Participant version; *CGI*-*R* Clinical Global Impression – Researcher version; *SAI* Schedule for Assessment of Insight; *CGI-C* Clinical Global Impression – Capacity version

^a^Incorporates questions from the Alcohol Use Disorders Identification Test (AUDIT) and the Drug Abuse Screening Test (DAST)

#### Sample characteristics

To characterise the sample and confirm eligibility, baseline information on demographics, legal status, offending history, medication regime stability, level of service engagement, alcohol/drug use measured using questions from the Alcohol Use Disorders Identification Test [57] and the Drug Abuse Screening Test [58] and other treatments are gathered via interview with the participant, consultation with their referrer and/or treatment provider and review of clinical records. Additional measures administered only at baseline include the Clinical Interview for Psychotic Disorders (CIPD; to confirm and record diagnosis) [59], the Brief Neurocognitive Assessment (BNA; to measure baseline cognitive functioning) [60] and the Internalised Stigma of Mental Illness questionnaire (ISMI; to determine eligibility for self-stigma trial) [54].^6^

#### Primary outcomes

We made an a priori decision to seek to progress to a definitive trial if we (1) achieved our target recruitment figure (*n* = 60) over the recruitment window and (2) acquired end-of-treatment (8 weeks) MacCAT-T data from ≥ 75% of those randomised. The MacCAT-T assesses participants on 4 domains of treatment decision-making capacity: (i) ‘understanding’, scored 0–6 (3 items); (ii) ‘reasoning’, scored 0–8 (4 items); (iii) ‘appreciation’, scored 0–4 (2 items); and (iv) ‘expressing a choice’, scored 0–2 (1 item) [61]. Higher scores indicate greater ability in each domain. We define data completion as the number of participants completing a MacCAT-T assessment at week 8 divided by the number of participants randomised to treatment or control.

#### Secondary outcomes

Secondary outcomes relate to data completion rates on the MacCAT-T at follow-up and planned secondary outcome measures at end-of-treatment and follow-up. We measure psychotic symptoms with the Positive And Negative Syndrome Scale (PANSS) [62], subjective recovery with the Questionnaire on the Process of Recovery (QPR) [63], quality of life with the Schizophrenia Quality of Life scale (SQoL) [64], service use with the Client Service Receipt Inventory (CSRI) [65], anxiety with the Beck Anxiety Inventory [66], core schemata with the Brief Core Schema Scale (BCSS) [67] and depression with the Calgary Depression Scale for Schizophrenia (CDSS) [68]. To assess feasibility of measuring psychological mechanisms (i.e., mediators of efficacy), we assess self-esteem with the RSES [53], data-gathering with the Beads Task (85:15 version) [55] and self-stigma with the Structured Interview Measure of Stigma (SIMS) [69].

#### Adverse events

We administer a range of other measures to detect any evidence of harm or threats to acceptability, following an adapted version of a previously used protocol [70, 71]. We record serious adverse events (SAEs; suicidal crisis, suicide attempts, suicide, death for reasons other than suicide, symptom exacerbation, readmission, other medically important events) and mild to moderate events (e.g., temporarily heightened distress). We define suicidal crisis without attempt as a score of 2 on item 8 of the CDSS. Severe symptom exacerbation is defined as a rating of ≥ 6 on a patient or researcher-rated Clinical Global Impression Severity (CGI-S) and Clinical Global Impression Improvement (CGI-I) scales [72, 73]. Both the patient and researcher-rated CGI-S are scored from 1 to 7, with higher scores indicating greater symptom severity; the CGI-I scales are also scored from 1 to 7, with higher scores indicating less improvement. Masked assessors administer the suicidality measure (item 8 of CDSS) at 0, 8 and 24 weeks, and the symptom exacerbation measures (patient and researcher rated CGI-I and CGI-S) at 8 and 24 weeks only. Non-masked therapists also complete these measures at the start of every intervention or control session. To assess mild to moderate adverse events, the number of participants stating they agree ‘quite a lot’ or ‘very much’ (corresponding to a score of 3 or 4, respectively) with each item on a self-report measure of adverse events (the Adverse Experiences in Psychotherapy questionnaire; AEP)^7^ at weeks 8 and 24 is recorded. Participants who leave the study early are invited to complete a parallel version of the AEP, designed to assess whether an adverse event led to their early discontinuation.

#### MacCAT-T construct validity

As part of the assessment of the MacCAT-T construct validity, the Schedule for Assessment of Insight (SAI)^8^ [74] is administered with patients, while their referrers or treatment providers are asked to complete a version of the Clinical Global Impression scale modified to assess (in) capacity (CGI–Capacity). Both are administered at 0, 8 and 24 weeks.

### Intervention and control procedures

Table 3 details the shared and specific components of the clinical procedures, each of which last 6 h and are delivered over an 8-week window. The default model of delivery is weekly 1-h sessions; however, this can be adjusted (e.g. shorter and more frequent sessions can be provided). The therapy window is deliberately short because we anticipate services and/or clinicians would be unwilling or unable to wait too long for a person to regain capacity before proceeding with treatment. Sessions will be recorded for supervision and a random sample will be assessed for adherence and competence.

**Table 3:** Details of interventions and control procedures

Key features & components of the interventions and control condition Self-stigma = A, Self-esteem = B, JTC = C, Control = D					Session
Engagement, listening, positive regard, empathy, collaboration	A	B	C	D	1–6
Structured & manualised to ensure focus, fidelity and homogeneity	A	B	C	D	1–6
Between-session activity for participant	A	B	C	D	1–6
Provision of structured self-help material relating to mechanism	A	B	C	-	1–6
Therapeutic work on non-targeted causal mechanisms excluded	A	B	C	D	1–6
Psychological formulation of causal mechanism and capacity (during trial)	A	B	C	-	1–2
Normalising via presentation of destigmatising written/audio-visual material	A	-	-	-	1–2
Behavioural experiments & anti-stigma data logs to reduce stigma beliefs and strengthen non-stigmatising illness beliefs	A	-	-	-	3–4
Identifying & improving positive-self beliefs, building self-confidence & reducing negative-self beliefs. Use of positive stimuli	-	B	-	-	1–2
Positive data log; positive activity planning (connection to others; being active; learning and giving); strengthening positive-self beliefs	-	B	-	-	3–4
Education about JTC bias, exercises to generate alternative explanations & increase evidence-gathering	-	-	C	-	1–2
Identification and modification of positive beliefs about JTC decision-making, building positive beliefs about evidence-gathering, & practice of non-JTC decision-making	-	-	C	-	3–4
Practice of new strategies and development of shared plan to maintain gains	A	B	C	-	5–6
Assessment only: history taking, additional psychometrics & neuropsychological assessment of factors affecting capacity (formulation after trial completion)	-	-	-	D	1–6
Between session tasks focused on aiding assessment (e.g., life event timeline)	-	-	-	D	1–6

Each intervention and the control condition are delivered by the same therapists, according to structured and manualised protocols. Therapists were either clinical psychologists who had trained in cognitive behavioural therapy (CBT), or CBT therapists accredited by the British Association for Behavioural and Cognitive Psychotherapies. Initial and ongoing training on the clinical protocols are provided by the Chief Investigator (CI) in conjunction with local site Principal Investigators (PIs), who also provide therapists with regular individual supervision. To refine the clinical procedures, therapists are asked to keep a written diary to record what they perceived to be the positive and challenging aspects of intervention delivery. Clinical procedures were discontinued if a participant experienced an SAE which the CI and/or an independent clinical member of the TSC judged to be caused by those procedures and discontinuation would not cause them further harm.

All clinical procedures involve non-specific therapeutic elements of engagement, listening, positive regard, empathy and collaboration. They are all structured, agenda-driven and manualised, and all involve between-session activity for the participant (i.e., ‘homework’). In the interventions, the between-session activity is focused on understanding and/or resolving the target psychological mechanism (whether low self-esteem, self-stigma or the JTC bias), whereas in the control condition it is focused on gathering additional information to enable further assessment of factors which may affect their capacity (e.g., completion of questionnaires or completing a life event timeline). The interventions follow the principles of cognitive-behavioural therapy for psychosis (CBTp) [75]. However, unlike traditional CBTp where therapy goals are often decided in collaboration with the patient, the interventions here are focused on a specific mechanism and the specific outcome of improving capacity, although effort is made to relate this to the personal goals of the participant.

The content of the self-stigma intervention is focused on negative beliefs about schizophrenia, psychosis and psychotic symptoms, and their potential effect on treatment decision-making. Building on previous work [33], it involves provision of normalising and destigmatising information, or completion of behavioural experiments and anti-stigma data logs focused on challenging stigma-related beliefs, or building and strengthening alternative non-stigmatising ones. Building on the work of others [36], the self-esteem intervention is focused on beliefs about the self and their potential relationship to decision-making about treatment. Only it involves strengthening positive-self beliefs and weakening negative-self ones via the use of a positive data log or activity planning, for example. The JTC intervention is focused on the JTC bias. Adapted from a version developed for an earlier trial [22], which was in turn a distilled version of a module taken from Metacognitive Training (MCT) [76], it involves explaining this bias to participants, raising awareness of its potential effects on treatment decision-making, and encouragement of greater evidence-gathering.

The aim of the control condition is simply to gather more information on factors which may help or hinder the participant’s treatment decision-making. It includes administration of additional psychometric measures, interviews and/or questionnaires. The therapist merely assesses; they do not provide feedback, try to increase understanding, or conduct formulation. However, once a participant completes the trial, the therapist recontacts them and their clinician (if the participant consents), to offer a psychological formulation focused on understanding their impaired decision-making, with recommendations to support it. We tested the acceptability and safety of this overall approach in a previous case series [25].

### Treatment as usual (TAU)

TAU for inpatients with psychosis in the UK typically involves regular assessment and care from NHS psychiatrists, nurses and other professionals. Pharmacological treatment with antipsychotic medication is nearly always offered [77]. This is administered either orally and/or via injection and in many cases involuntarily. Cognitive behavioural therapy (CBT) is offered to approximately half of inpatients [77], in addition to other psychosocial interventions such as art therapy or occupational therapy. TAU for outpatients with psychosis typically involves assessment and care from a community mental health team (CMHT). These involve a range of professionals, including psychiatrists, psychiatric nurses, clinical psychologists and occupational therapists. Patients are usually prescribed antipsychotic medication and approximately one quarter are offered CBT [77]. In England, but not Scotland, all people experiencing their first episode psychosis are required to be offered rapid intervention and support from specialist Early Intervention in Psychosis services. Both England and Scotland allow for the use of Community Treatment Orders, whereby outpatients are required to adhere to pharmacological treatments or be readmitted to hospital. Focused psychological support for treatment decision-making capacity is likely to be rare at best, regardless of setting.

Although TAU may—and indeed ought to—have beneficial effects on the outcomes targeted by DEC:IDES, randomisation should ensure these are evenly distributed across the treatment and control groups. Service usage will be measured at baseline, EoT and FU and any between-group differences will be noted.

### Analysis

A Statistical Analysis Plan (SAP; see Additional file 1) has been prepared by PJT prior to data entry, in conjunction with PH, RE and ND.

#### Characterising the sample

All baseline data will be reported for the sample as a whole and per arm of each trial. All quantitative participant baseline characteristics will be summarised by numbers and percentages, mean and standard deviation (SD), or median and interquartile range (IQR), as appropriate.

#### Primary outcomes

Data completion rates at 8 weeks post-randomisation (end of treatment) on the MacCAT-T will be presented as a percentage with 95% CIs, for the overall study, per trial and per arm of each trial. Proportion of the recruitment target actually recruited will be reported as a percentage with 95% CIs. We will also report standardised and unstandardised effect sizes for group differences within each trial on the MacCAT-T at 8 weeks, with 95% CIs. Due to very limited power, these will not be subject to any efficacy-related interpretation. Effect sizes will be reported for both the (i) ‘as randomised’ (intention-to-treat; ITT) sample and (ii) those randomised who also received ≥ 3 h of their allocated clinical procedures—i.e., a ‘per-protocol’ population.

#### Secondary outcomes

Data completion rates for all planned secondary efficacy and mechanism outcomes will be presented as a percentage with 95% CIs, for the overall study, per trial and per arm of each trial. Effect sizes and 95% CIs for each efficacy and mechanism outcome will be reported, for both the ITT and per-protocol samples. We will also report the number of blind breaks.

#### Adverse events

For each treatment and control group, we will report number of (i) deaths by suicide, (ii) deaths not caused by suicide, (iii) participants attempting suicide, (iv) participants with suicidal crises, (v) participants experiencing severe symptom exacerbation and (vi) participants stating they agree ‘quite a lot’ or ‘very much’ (corresponding to a score of 3 or 4, respectively) with each item on the AEP. We will also report any other medically important SAEs. Whether any SAEs were judged by an independent clinical expert, Sponsor and/or NHS REC to be causally related to research and/or clinical procedures will also be reported.^9^

#### Qualitative study analyses

For qualitative study 1, framework analysis will be applied to interview recordings in line with the approach outlined by Gale (2013) [45]. This involves transcription, familiarisation and coding; development and application of a wider analytical framework; and charting data into the framework matrix and interpretation. We will use case study methodology for qualitative study 2. Analysis of interviews and research data will be structured using Yin’s (2014) overall ‘explanation building’ framework [47]. The steps involve making an initial explanatory proposition and comparing the findings of an initial case against this proposition. This is then revised, and other details of the case and any additional cases are compared against the revision. This process is repeated as many times as needed.

#### MacCAT-T validation

We will calculate and report the correlation between (i) MacCAT-T appreciation ratings and SAI ratings and (ii) MacCAT-T total scores and clinician CGI ratings of capacity, both with 95% CIs.

### Service user and carer involvement

Service users and carers were involved at a number of stages in the project. We held a knowledge exchange event where we sought their views on the value of our intended research programme, and several service users reviewed the design and content of our participant information sheets. Two service users also joined our Trial Steering Committee and provided oversight and guidance on project completion.

### Changes to protocol

Several changes were made to the study protocol at different phases of the project and received ethical approval where required. Their timing and purpose is outlined in Table 4. All the changes that occurred between the study being publicly registered and the first randomisation involved mitigation of pandemic-related health risks to participants and staff. The majority of the changes that occurred after the first randomisation involved mitigation of the effects of the pandemic on recruitment, with the main ones being the extension of the recruitment period in the Lothian site, increasing the numbers allowed to take part in the individual trials and allowing previous trial completers to return to take part in one of the other trials, if eligible. Preferential allocation to the self-esteem trial was introduced primarily to mitigate the much lower than expected prevalence of low self-esteem in this population, a finding which will be discussed further when we report our results.

**Table 4:** Timing and purpose of changes to protocol

**Description of change**	**Date of sponsor approval**	**Project phase**	**Reason(s) for change**
RSES replaced RSQ	17/2/20	Pre-registration	To improve methods (e.g., in light of new information)
CIPD replaced SCID	17/2/20	Pre-registration	To reduce or save research costs
BCSS added	17/2/20	Pre-registration	To improve methods (e.g., in light of new information)
CSRI added	17/2/20	Pre-registration	To improve methods (e.g., in light of new information)
Randomisation sequence parameters changed	17/2/20	Pre-registration	To improve methods (e.g., in light of new information)
English docs updated with Scottish REC changes	26/9/20	Pre-randomisation	To align English & Scottish REC approved protocols
COVID-19 information sheet introduced	3/12/20	Pre-randomisation	To mitigate pandemic health risks
Remote consent introduced	3/12/20	Pre-randomisation	To mitigate pandemic health risks
Remote clinical procedures introduced	3/12/20	Pre-randomisation	To mitigate pandemic health risks
Remote research assessments introduced	3/12/20	Pre-randomisation	To mitigate pandemic health risks
COVID-19 protocol introduced	3/12/20	Pre-randomisation	To mitigate pandemic health risks
Newspaper recruitment advert launched	8/2/21	Post 1st randomisation	To mitigate the effect of the pandemic on recruitment
Bus stop recruitment adverts launched	17/5/21	Post 1st randomisation	To mitigate the effect of the pandemic on recruitment
Recruitment window in Lothian extended	1/7/21	Post 1st randomisation	To mitigate the effect of the pandemic on recruitment
Lothian research staff reduced	1/7/21	Post 1st randomisation	To mitigate the effect of the pandemic on recruitment via rebudgeting of research costs
English site opening delayed (without extending recruitment window)	1/7/21	Post 1st randomisation	To mitigate the effect of the pandemic on recruitment
Independent statistician replaced by PJT	1/7/21	Post 1st randomisation	To mitigate the effect of the pandemic on recruitment via rebudgeting of research costs
CIPD dropped at 8 and 24 weeks	1/7/21	Post 1st randomisation	To fix an error
English site closure delayed (without extending recruitment window)	8/6/22	Post 1st randomisation	To mitigate the effects of NHS staffing problems and the pandemic on recruitment
Recruitment window in Lothian extended	8/6/22	Post 1st randomisation	To mitigate pandemic health risks, its effects on recruitment, and the effect of NHS staffing problems
More than 20 participants allowed to participate in self-stigma or jumping to conclusions trials	20/8/22	Post 1st randomisation	To mitigate the effects of the pandemic and low prevalence of self-esteem on recruitment, and to ensure best use of research and treatment costs
Preferential allocation to self-esteem trial introduced	20/8/22	Post 1st randomisation	To mitigate the impact of the low prevalence of low self-esteem on recruitment
Previous participants allowed to return to take part in 1 of the other trials (if eligible)	20/8/22	Post 1st randomisation	To mitigate the effects of the pandemic and low prevalence of self-esteem on recruitment, and to ensure best use of research and treatment costs
Randomisation to treatment or control allowed to happen before clinical session 1 in some cases	27/9/22	Post 1st randomisation	To improve feasibility
Lothian sample size increased by 1 (45 to 46)	6/10/22	Post 1st randomisation	To fix an error
Study end date extended in all sites (without extending recruitment window)	6/10/22	Post 1st randomisation	To mitigate the effect of NHS staffing problems and to complete other tasks

*Note*: *RSES* Rosenberg Self Esteem Scale; *RSQ* Robson Self-concept Questionnaire; *BCSS*, Brief Core Schema Scale; *CSRI*, Client Service Receipt Inventory; *CIPD*, Clinical Interview for Psychotic Disorders; *PJT*, Dr Peter James Taylor, University of Manchester

## Discussion

A high proportion of inpatients diagnosed with schizophrenia-spectrum disorders are assessed as not being able to make their own decisions about their psychiatric treatment [78–80]. Few, however, receive support to regain it [4, 16, 17, 19]. Although various authorities have provided generic suggestions for ways to support capacity, such as simplification and repetition of information, the reduction of anxiety or sedation, delaying the decision, or speech and language therapy [14, 15, 19–21], such strategies are unlikely to be sufficient for people with complex or severe conditions such as psychosis. Regardless, even these strategies are rarely implemented [16].

The recent Wessely and Scott reviews [20, 21] both recommend using the well-established approaches of advocacy and advance statements^10^ to support people to make decisions about their psychiatric treatment. However, while evidence suggests these approaches may be important for supporting people to communicate and implement these decisions [81–85], particularly in the context of power asymmetries between patients and clinicians, they make no claim to identify and/or mitigate threats to the internal, psychological processes from which those decisions emerge [85, 86]. While these approaches may support the exercise of legal capacity, they do not seek to rectify impaired mental capacity.

The aim of DEC:IDES is to begin the much-needed process of developing approaches that try to directly restore capacity for people diagnosed with schizophrenia-spectrum disorder. To say that there is much work to do is an understatement. The Code of Practice for the Adults with Incapacity Act (Scotland) (2000), states ‘Every possible assistance must be given to the adult to understand his or her own medical condition and the decision that is required in relation to treatment.’ and ‘There is an absolute obligation to facilitate the exercise of capacity, where possible.’ [15]. In England and Wales, the corresponding Code of Practice for the Mental Capacity Act (2005) states ‘All practical and appropriate steps must be taken to help people to make a decision for themselves.’ [14]. In the two decades since the laws underpinning these directives were passed, there have been no notable advances in what counts as ‘possible’ for people with psychosis, and no new ‘practical and appropriate steps’ have been developed [5, 19].

We need to make up for this lost time. People with psychosis cannot wait another 10–20 years for the emergence of effective and safe interventions to support their treatment decision-making. Ambitious new methodologies are needed to accelerate the intervention-development process. Demonstrating the feasibility of DEC:IDES will have significant implications not only for those seeking to support capacity in psychosis, but also those who wish to accelerate the development of interventions for other conditions.

DEC:IDES compares interventions to an attention control, rather than usual care alone. Some consider usual care alone to be a better comparator because it best represents a problem’s natural course; however, we consider this to be moot given the confounding effects of regression to the mean.^11^ More importantly, if we are to make evidence-based justifications for the time and cost involved in training people to deliver a complex intervention, then demonstrating superiority to a non-specific control is required at some stage in the developmental ‘pipeline’. Not introducing this early either unnecessarily extends the length and expense of this pipeline or, if introduced later, increases uncertainty over both feasibility and efficacy *after* significant investment has already been made. Although early use of a non-specific control might increase the risk of type II error in relation to the non-specific benefits of a complex intervention, we do not think confirming that these exist is worth the investment.

DEC:IDES has a number of other key features. We have planned a detailed assessment of adverse events, thus allowing any early signs of harm to be detected. Assessor-blinding will allow us to quantify the potential risk of blind-breaks in a larger trial. Finally, involvement of multiple sites helps us identify and resolve challenges to working across the different legal jurisdictions of England and Scotland. Demonstrating that we can deliver a multi-site trial at this stage will be essential for assessing the feasibility of a larger trial, where multiple sites will be essential.

The main limitation of DEC:IDES is that we did not design it to be adaptive. That is, we did not build in a priori interim analyses of feasibility together with the flexibility to drop, extend or replace trials. We anticipated this to be too challenging to navigate at this early stage, particularly given existing funding structures. However, if DEC:IDES is successful, then demonstrating adaptivity will be a key future objective. Another limitation is that our pre-specified progression criteria were lacking in detail. Although we clearly stated we would seek to proceed to a definitive trial if we met our recruitment and data retention objectives, we failed to specify the consequences of not doing so. Unlike other feasibility trials [87], we did not outline in advance the circumstances under which we would continue the research programme with modifications, or simply discontinue it. It is important to note that DEC:IDES was conducted almost entirely during the global COVID-19 pandemic. Drawing inferences from trials conducted during this period is going to be challenging, but if DEC:IDES is feasible in this unusually adverse context, then it is likely to be feasible in others. Finally, we did not formally assess alignment between patient and intervention goals. Although we hope our qualitative interviews will shed light on whether any such misalignment is a threat to acceptability, a future trial might consider assessing this more systematically—not least because ambivalence about support for treatment decision-making is reasonable to expect in this group.

DEC:IDES was initially funded for 19 months. Staff were appointed in December 2019, and recruitment commenced in late February 2020, before being paused almost immediately due to the pandemic. A major rebudgeting and reallocation of resources was agreed with the funder which, together with a small amount of additional funding, enabled the overall duration to increase to 34 months. Recruitment recommenced in October 2020, but with reduced research staff and significant pandemic-related constraints, including no access to psychiatric inpatient wards and no face-to-face contact with community participants. Recruitment closed in October 2022. The final post-treatment assessments will be completed on 31 March 2023. Study results will be published shortly after. If DEC:IDES achieve its feasibility goals, we anticipate that the next step will be a definitive Umbrella trial, with a sample size sufficient to make inferences about efficacy.

### Supplementary Information

**Additional file 1:** Statistical Analysis Plan (SAP).

**Additional file 2:** Supplementary Appendix.

## Data Availability

Data collected in this study will be stored for 10 years and made available for audit by ENU or NHS approved staff. We made an a priori decision that, because the small sample size increases the risk of participant identification, it would not be available to other researchers.

## Footnotes

1 The number of participants eligible for follow-up increased when the recruitment window was increased to mitigate the impact of the COVID-19 pandemic. See CONSERVE checklist.

2 The first UK-wide COVID-19 stay-at-home order (‘lockdown’) was announced 7 days later, on 23 March 2020.

3 Audio recording of consent was introduced as an alternative to written consent in order to reduce face-to-face contact during the COVID-19 pandemic. See CONSERVE checklist.

4 On 2 September 2022, an amendment was approved to allow preferential allocation to the self-esteem trial. This was introduced to mitigate the negative effect on recruitment of an unexpectedly low prevalence of low self-esteem in this population. It means that if a participant is eligible for the self-esteem trial, they are non-randomly allocated to it regardless of whether they are also eligible for the self-stigma and/or JTC trials. Participants who are eligible only for the latter two trials continue to be randomly allocated to one of them.

5 On 28 November 2022, an amendment was submitted to allow R2 to proceed prior to session 1 if the randomisation administrator was unavailable at the time of session 1 (e.g. due to leave or illness).

6 See the Statistical Analysis Plan (SAP) in appendix for further specification.

7 Hutton, P., Byrne, R., & Morrison, T. (2017). Adverse effects in psychotherapy measure. Unpublished manuscript.

8 We intended to use the Expanded Schedule for the Assessment of Insight; however, an error led to the original version being used instead.

9 We will also report which events were identified via research assistants who were blind to treatment arm allocation (assessed at weeks 0, 8 and 24) and which events were identified via clinicians who were not blind to allocation (assessed at start of each clinical session).

10 The Wessely and Scott reviews use the terms ‘Advance Choice Documents’ and ‘Advance Choice’, respectively.

11 This is most likely to occur when (a) the problem fluctuates and does not follow an inevitably deteriorating course, (b) the baseline assessment of problem severity takes place because the problem meets a specified threshold of severity and (c) the post-treatment assessment takes place because a fixed time-point has been reached.
