# Correction: Accelerating the development of a psychological intervention to restore treatment decision‑making capacity in patients with schizophrenia‑spectrum disorder: a study protocol for a multi‑site, assessor‑blinded, pilot Umbrella trial (the DEC:IDES trial)

**DOI:** 10.1186/s40814-023-01376-1

**Published:** 2023-08-14

**Authors:** Paul Hutton, James Kelly, Christopher D. J. Taylor, Brian Williams, Richard Emsley, Candy Ho Alexander, Anvita Vikram, David Saddington, Andrea McCann, Joseph Burke, Emma Eliasson, Sean Harper, Thanos Karatzias, Peter J. Taylor, Andrew Watson, Nadine Dougall, Jill Stavert, Suzanne O’Rourke, Angela Glasgow, Regina Murphy, Karen Palmer, Nosheen Zaidi, Polly Bidwell, Jemma Pritchard, Lucy Carr, Amanda Woodrow

**Affiliations:** 1 School of Health & Social Care, Edinburgh Napier University, Edinburgh, UK; 2 Edinburgh Research & Innovation Centre for Complex and Acute Mental Health Problems, Edinburgh, UK; 3 Faculty of Health & Medicine, Lancaster University, Lancaster, UK; 4 Lancashire & South Cumbria NHS Foundation Trust, Preston, UK; 5 Pennine Care NHS Foundation Trust, Ashton‑Under‑Lyne, UK; 6 Division of Psychology & Mental Health, Manchester Academic Health Sciences Centre, University of Manchester, Manchester, UK; 7 School of Health, Social Care & Life Sciences, University of the Highlands and Islands, Inverness, UK; 8 Institute of Psychiatry, Psychology & Neuroscience, Kings College London, London, UK; 9 NHS Lothian, Edinburgh, UK; 10 NHS Research Scotland Mental Health Network, Edinburgh, UK; 11 National Centre for Suicide Research and Prevention, Karolinska Institutet, Stockholm, Sweden; 12 School of Health in Social Science, University of Edinburgh, Edinburgh, UK

**Correction: Pilot Feasibility Stud 9, 117 (2023)**

**https://doi.org/10.1186/s40814-023-01323-0**

Following publication of the original article [1], the authors reported that the Additional file 2: Supplementary Appendix was not published together with the article.

The original article [1] has been updated.

### Supplementary Information

**Additional file 2:** Supplementary Appendix.
