# Supplementary material for: Accelerating the development of a psychological intervention to restore treatment decision-making capacity in patients with schizophrenia-spectrum disorder: a study protocol for a multi-site, assessor-blinded, pilot Umbrella trial (the DEC:IDES trial)
Source: Pilot Feasibility Stud. 2023 Jul 8;9:117. doi: 10.1186/s40814-023-01323-0 (PMC10329297; doi:10.1186/s40814-023-01323-0)
Supplement: Supplementary file 1 — Additional file 1: Statistical Analysis Plan (SAP). [file 40814_2023_1323_MOESM1_ESM.docx]

**Statistical Analysis Plan (SAP)**

**Title of the Trial:** DEcision-making Capacity: Intervention Development & Evaluation in Schizophrenia-spectrum Disorder (DEC:IDES)

**Trial registration number:** NCT04309435

**SAP version number with dates: v4 3^rd^ March 2023**

**Protocol version number and date: v9 6^th^ December 2022**

**SAP revisions:**

| Protocol version | Updated SAP version number | Section number changed | Description of and reason for change | Date changed | Initials |
| --- | --- | --- | --- | --- | --- |
| *9* | *2* | *2.5.3*  *5.2.3*  *5.2.7* | *2.5.3: Clarified that DMEC part of TSC*  *5.2.3: Threshold for defining an individual total or subscale score as missing and non-imputable was reduced from >30% missing to >25%, to be consistent with the ≤25% goal employed for missing data across the sample, on the planned primary outcome at EoT.*  *5.2.7: Specified SPSS and r as software packages* | *30/1/2023* | *PH* |
| *9* | *3* | *1.1*  *5.2 (q)* | *1.1. Significantly shortened background section and referred readers to main protocol instead*  *5.2 (q). Added summary of analysis to be applied in qualitative study 2 (MacCAT-T validity)* | *12/2/2023* | *PH* |
| *9* | *4* | *5.2 (q)*  *5.2.2*  *5.2.6*  *5.2.7* | *5.2 (q) Separated qualitative and quantitative analyses into separate questions*  *5.2.2. Added bootstrapping as method for dealing with non-normal estimates*  *5.2.6 Added details of analysis to be used to calculate effect sizes for planned primary outcome, and other outcomes*  *5.2.7 Replaced R with Stata* | *03/03/2023* | *PH* |
| *9* | *5* | *5*  *5.2 (r)*  *5.2 (s)* | *5. Added additional reporting guidelines*  *5.2 (r) Added details of planned analysis*  *5.2 (s) Added details of planned analysis* | *09/03/2023* | *PH* |
| *9* | *6* | *4.71*  *5.2 (r)*  *5.2 (s)* | *4.7.1 Change from E-SAI to SAI noted*  *5.2 (r) Added baseline data to analysis of concordance*  *5.2 (s) Changed analysis of MacCAT-T total scores to polyserial from polychoric. Added baseline data to analysis of concordance.* | *10/03/2023* | *PH* |

**Roles and responsibility:** *names, affiliations and roles of SAP contributors*

**Roles and responsibility:** Signatures

| **SAP Author:** | | |
| --- | --- | --- |
| Signature: ...................................................................................................... |  | Date:  10.03.2023 |
| Name: (please print):  Dr Peter Taylor |  |  |

| **Senior Statistician:** | | |
| --- | --- | --- |
| Signature: .................................................................................................... |  | Date:  10.03.2023 |
| Name: (please print):  Professor Richard Emsley |  |  |

| **Chief Investigator:** | | |
| --- | --- | --- |
| Signature: ...................................................................................................... |  | Date:  10.03.2023 |
| Name: (please print):  Professor Paul Hutton |  |  |

**Table of contents**

[1 Introduction 5](#_Toc129380898)

[1.1 Background and rationale 5](#_Toc129380899)

[1.2 Aims and Research Questions 5](#_Toc129380900)

[1.2.1 Study Aims 5](#_Toc129380901)

[1.2.2 Study Research Questions 5](#_Toc129380902)

[2 Trial Methods 6](#_Toc129380903)

[2.1 Trial design 6](#_Toc129380904)

[2.2 Randomisation details 6](#_Toc129380905)

[2.3 Sample size 7](#_Toc129380906)

[2.4 Framework 7](#_Toc129380907)

[2.5 Statistical interim analysis and stopping guidance 7](#_Toc129380908)

[2.5.1 Information on Interim analyses specifying what interim analyses will be carried out and listing of time points 7](#_Toc129380909)

[2.5.2 Any planned adjustment of the significance level due to interim analysis 7](#_Toc129380910)

[2.5.3 Details of guidelines for stopping a trial early 7](#_Toc129380911)

[2.6 Timing of final analysis 7](#_Toc129380912)

[2.7 Timing of outcome assessments 8](#_Toc129380913)

[3 Statistical Principles 9](#_Toc129380914)

[3.1 Confidence intervals and P-values 9](#_Toc129380915)

[3.2 Adherence and protocol deviations 9](#_Toc129380916)

[3.2.1 Definition of adherence to the intervention and how this is assessed including extent of exposure 9](#_Toc129380917)

[3.2.2 Description of how adherence to the intervention will be presented 10](#_Toc129380918)

[3.2.3 Serious breach of protocol requirements 10](#_Toc129380919)

[3.2.4 How serious protocol breaches will be reported 10](#_Toc129380920)

[3.3 Analysis populations 10](#_Toc129380921)

[4 Trial Population 11](#_Toc129380922)

[4.1 Screening data 11](#_Toc129380923)

[4.2 Eligibility criteria 11](#_Toc129380924)

[4.3 Inclusion Criteria 11](#_Toc129380925)

[4.4 Exclusion Criteria 11](#_Toc129380926)

[4.5 Recruitment 11](#_Toc129380927)

[4.6 Withdrawal/follow-up 12](#_Toc129380928)

[4.6.1 Level of withdrawal 12](#_Toc129380929)

[4.6.2 Timing of withdrawal/ lost to follow up data 12](#_Toc129380930)

[4.6.3 Presentation of ‘lost to follow up’ data 12](#_Toc129380931)

[4.7 Baseline patient characteristics 12](#_Toc129380932)

[4.7.1 List of Baseline Characteristics 13](#_Toc129380933)

[4.7.2 Methods to Summarise Baseline Characteristics 14](#_Toc129380934)

[5 Analysis 15](#_Toc129380935)

[5.1 Primary analyses 15](#_Toc129380936)

[5.2 Secondary analyses 16](#_Toc129380937)

[5.2.1 Methods used to check assumptions of statistical methods 22](#_Toc129380938)

[5.2.2 Details of alternative methods to be used if distributional assumptions do not hold 22](#_Toc129380939)

[5.2.3 Missing data 23](#_Toc129380940)

[5.2.4 Sensitivity analyses 23](#_Toc129380941)

[5.2.5 Subgroup analyses 23](#_Toc129380942)

[5.2.6 Additional analyses 23](#_Toc129380943)

[5.2.6.1 Analyses of planned primary outcome 23](#_Toc129380944)

[5.2.6.2 Analyses of other outcomes 24](#_Toc129380945)

[5.2.7 Software 24](#_Toc129380946)

[6 References 25](#_Toc129380947)

[7 Appendix 26](#_Toc129380948)

# Introduction

## Background and rationale

*(See main protocol for more detailed synopsis)*

Our aim is to conduct the first Umbrella trial in mental healthcare, using this to accelerate the development of the first evidence-based intervention to support treatment decision-making capacity in psychosis. The DEC:IDES (‘DEcision-making Capacity: Intervention Development and Evaluation in Schizophrenia-spectrum disorders’) trial involves running three ‘interventionist-causal’ randomised controllled trials (IC-RCTs) in parallel, each testing the effect on capacity of an intervention to either reduce self-stigma, improve self-esteem or reduce the jumping to conclusions bias. Our aims at this stage are restricted to demonstrating feasibility, acceptability and safety. Our primary objectives are to demonstrate feasibility of recruitment and determine data quality and completion rates for the MacArthur Competence Assessment Tool for Treatment (MacCAT-T) (1), our planned primary outcome in a future trial. Our secondary objectives include assessing adverse events, data completion rates for secondary efficacy and mechanism outcomes, participant and clinician acceptability of the trial, and the construct validity of the MacCAT-T.

## Aims and Research Questions

### Study Aims

| **Aims** | **Linked RQs** |
| --- | --- |
| Aim 1: Test data quality for a definitive trial (primary aim) | RQ 1,2,5 |
| Aim 2: Test recruitment and retention rates for a definitive trial | RQ 2,3 |
| Aim 3: Test trial procedures for a definitive trial | RQ 3,4 |
| Aim 4: Assess acceptability of interventions for this group | RQ 3,4 |
| Aim 5: Refine treatment protocols to ensure feasibility in clinical settings | RQ 3,4 |
| Aim 6: Consolidate research infrastructure to support main trial | RQ 3,4 |

### Study Research Questions

| **Research questions (RQ):** |
| --- |
| RQ 1: What data quality and completion rates can be obtained for the main outcome measures? |
| RQ 2: What proportion of eligible patients consent, engage, & complete the trial? |
| RQ 3: What do participants, clinicians & collaborators think will improve study processes, including interventions? |
| RQ 4: Are trial procedures acceptable to them, and feasible to implement? |
| RQ 5: What sample size is required in a full study? |
|  |

# Trial Methods

## Trial design

The planned study is a feasibility/pilot of a multi-site single (rater) blind Umbrella trial of psychological interventions to support treatment decision-making capacity in people diagnosed with non-affective psychosis (schizophrenia-spectrum disorders). It has been designed primarily to examine post-treatment (8 week) data retention rates for the planned primary outcome (MaCAT-T capacity ratings) in a future definitive trial. Participants will be randomly allocated to receive treatment as usual (TAU) plus a psychological intervention to improve either (i) self-stigma, (ii) self-esteem, or (iii) the jumping to conclusions reasoning bias, or TAU plus an attention control condition (see Fig. 1). Assessors blind and independent to treatment group will conduct assessments at baseline, post-treatment (8 weeks) and follow-up (6 months). To minimise piloting costs 75% of the 60 patient participants will be recruited in the lead site (NHS Lothian). Each intervention group will be compared to its own control group (each receive the same standardised procedure) to ensure participants in each trial are equivalent with respect to their presenting mechanism. Acceptability and feasibility of trial procedures (RQs 3 & 4) will also be examined through qualitative interviews with participants and referrers/clinicians, using Framework Analysis (2). Qualitative analysis of change in participant appreciation, as measured by the MaCAT-T, will be performed using qualitative case study methodology (3).


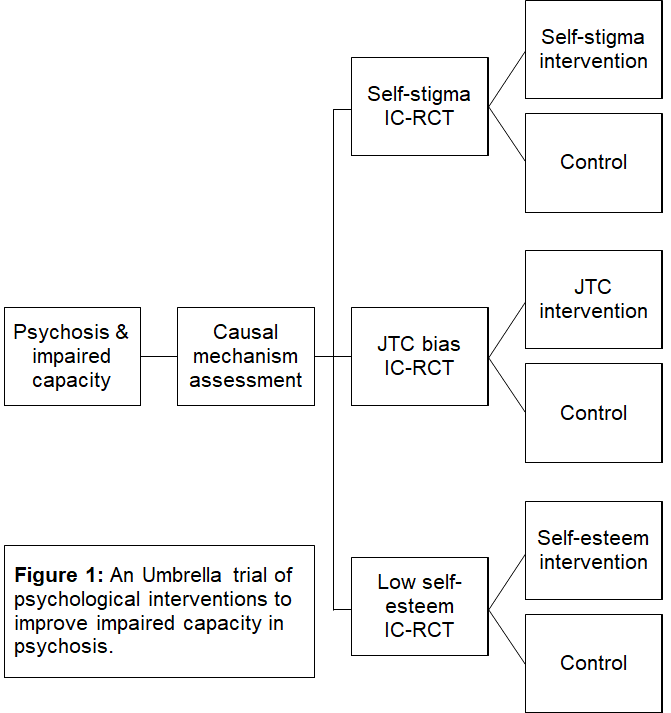


## Randomisation details

There will be two points of randomisation; randomisation to trial (‘R1’) and randomisation to treatment or control (‘R2’). R1 will be used to allocate individuals to a trial when they are eligible for more than one. Randomisation will be conducted by the automated and online service provided by Sealed Envelope,^[[1]](#footnote-1)^ using a concealed and randomly generated allocation sequence (without stratification or random permuted blocks) per each possible combination of trials; SE-SS, SE-JTC, SS-JTC and SS-JTC-SE. There will be no requirement to mask outcome assessors to the results of R1 because no comparisons between trials are planned. R2 will also be performed by Sealed Envelope, using a single concealed and randomly generated allocation sequence, stratified by trial and using random permuted blocks of 2 and 4. In order to minimise non-ignorable missing data (primary outcome 2), we aimed to perform R2 as late as possible, defined as the beginning of the participant’s first session with their therapist. The result of R2 will be communicated by email to the Chief Investigator, who will communicate the outcome by phone to the relevant therapist, who will subsequently inform the participant.

## Sample size

We calculated that 60 participants (20 per trial) would allow us to estimate a drop-out rate (i.e., data non-retention) of 15%, at week 8, to within a 95% confidence interval (95% CI) of +/- 9%. The formula we used is 95% CI = 1.96 x √(proportion x (1-proportion) / N), which translates to N = (proportion x (1- proportion)) / (95% CI / 1.96). We estimated the proportion of people with schizophrenia-spectrum disorders and impaired capacity who have at least one of our proposed causal mechanisms to range from 80-100%. Pilot trials suggested ≥80% of those who have one of these mechanisms would consent and be randomised, ≥85% of whom will complete the trial.

## Framework

This is a pilot/feasibility trial, with the aim of demonstrating acceptability and feasibility of research and clinical procedures.

## Statistical interim analysis and stopping guidance

### Information on Interim analyses specifying what interim analyses will be carried out and listing of time points

No interim analysis will be performed.

### Any planned adjustment of the significance level due to interim analysis

None

### Details of guidelines for stopping a trial early

For this pilot trial, the Trial Steering Committee (TSC) incorporates the functions of a Data Monitoring and Ethics Committee (DMEC). It will review all instances of adverse events, whether or not they are judged to be attributable to the trial or interventions, and, based on this information, determine whether the participant should be withdrawn and/or whether the trial should be suspended, stopped or continued.

## Timing of final analysis

All analyses will be performed at the end of the follow-up period, once all data are either collected or data from any participants with outstanding outcome data is declared missing as agreed by the CI and supervising statistician.

## Timing of outcome assessments

Baseline assessments will be completed before randomisation. End of treatment (EoT) assessments and follow-up assessments will commence at 56 days (8 weeks) and 168 days (24 weeks) post-randomisation.

# Statistical Principles

## Confidence intervals and P-values

Due to very limited power and the overriding feasibility/pilot aims of the project, all analyses of efficacy outcomes should be considered exploratory and/or hypothesis generating. Proportions, means, standard deviations, effect sizes (standardised and unstandardised) and corresponding 95% confidence intervals (CIs) for all time points will therefore be reported descriptively, on both a strict intention-to-treat and per-protocol basis (≥50% attendance in treatment or control).

## Adherence and protocol deviations

### Definition of adherence to the intervention and how this is assessed including extent of exposure

Therapists are required to try to maintain fidelity to the delivery of the intervention protocols on the features and components specified in Table 1. Therapist fidelity on these domains will be rated by clinical supervisors as present, partially present or absent for a randomly selected proportion of participants. Supervisors will also comment on what they believe are the reasons the component was present, partially present or absent. This information, together with written therapist reflections on obstacles to protocol delivery for each participant, will be used to improve our clinical procedures.

| **Table 1: Key features & components of the interventions and control condition** | | | | | |
| --- | --- | --- | --- | --- | --- |
| **Self-stigma = A Self-esteem = B JTC = C Control = D** | | | | | **Session** |
| Engagement, listening, positive regard, empathy, collaboration | A | B | C | D | 1-6 |
| Structured & manualised to ensure focus, fidelity and homogeneity | A | B | C | D | 1-6 |
| Between-session activity for participant | A | B | C | D | 1-6 |
| Provision of structured self-help material relating to mechanism | A | B | C | - | 1-6 |
| Therapeutic work on non-targeted causal mechanisms excluded | A | B | C | D | 1-6 |
| Psychological formulation of causal mechanism and capacity (during trial) | A | B | C | - | 1-2 |
| Normalising via presentation of destigmatising written/audio-visual material | A | - | - | - | 1-2 |
| Behavioural experiments & anti-stigma data logs to reduce stigma beliefs and strengthen non-stigmatising illness beliefs | A | - | - | - | 3-4 |
| Identifying & improving positive-self beliefs, building self-confidence & reducing negative-self beliefs. Use of positive stimuli | - | B | - | - | 1-2 |
| Positive data log; positive activity planning (connection to others; being active; learning and giving); strengthening positive-self beliefs | - | B | - | - | 3-4 |
| Education about JTC bias, exercises to generate alternative explanations & increase evidence-gathering | - | - | C | - | 1-2 |
| Identification and modification of positive beliefs about JTC decision-making, building positive beliefs about evidence-gathering, & practice of non-JTC decision-making | - | - | C | - | 3-4 |
| Practice of new strategies and development of shared plan to maintain gains | A | B | C | - | 5-6 |
| Assessment only: history taking, additional psychometrics & neuropsychological assessment of factors affecting capacity (formulation after trial completion) | - | - | - | D | 1-6 |
| Between session tasks focused on aiding assessment (e.g., life event timeline) | - | - | - | D | 1-6 |
|  |  |  |  |  |  |

Participant adherence is defined as attendance at a minimal proportion of the sessions. We considered participants to have had at least minimal adherence if they attended for at least 3 hours (50%) of the 6 available. Information from the qualitative interviews with participants will be used to refine our definition of adherence for a future definitive study.

### Description of how adherence to the intervention will be presented

Fidelity to the therapy domains described above will be presented as the proportion of participants where a given domain was present, partially present or absent during the reviewed sessions. For participants, we will report the average number of hours of contact with a therapist they receive and the proportion receiving at least 3 hours, with 95% CIs.

### Serious breach of protocol requirements

A serious breach is a breach which is likely to effect to a significant degree either the safety or physical or mental wellbeing of the participants in the study; or the scientific value of the study. If a potential serious breach is identified by the Chief investigator or delegates, the Sponsor will be notified within 24 hours. The Sponsor will assess the impact of the breach on the scientific value of the study, to determine whether the incident constitutes a serious breach and report to the relevant research ethics committee as necessary.

### How serious protocol breaches will be reported

All serious breaches of protocol requirements will be reported in narrative format, with description of what happened, when and why, and the actions taken in response.

## Analysis populations

Reporting of group differences on outcomes measures will be performed on both the (i) ‘as randomised’ (intention-to-treat; ITT) population, which means all participants randomised will be analysed according to the trial arm to which they are randomised (ii) those randomised who also received ≥3 hours of their allocated clinical procedures – i.e., a ‘per protocol’ (PP) population.

# Trial Population

## Screening data

A screening log will be used to identify:

1. the number of patients screened;
2. the percentage of those screened who are potentially eligible;
3. the proportion of those potentially eligible who consent;
4. the number eligible;
5. the proportion of those who consent who are confirmed as eligible

## Eligibility criteria

Participants with schizophrenia-spectrum disorder aged 18-65 and judged to lack the capacity to make decisions about their psychiatric treatment are the target population.

## Inclusion Criteria

To be included in the trial, an individual must be:

1. aged between 18 and 65 years;
2. able to be interviewed and complete the measures;
3. diagnosed with a schizophrenia-spectrum disorder (schizophrenia, schizoaffective disorder, delusional disorder, psychosis not otherwise specified, brief psychotic disorder);
4. presumed or already judged to have impaired treatment decision-making capacity;
5. registered as a patient with clinical or social care services

## Exclusion Criteria

An individual will be unable to take part if they:

1. have a moderate to severe learning disability;
2. have psychosis of a predominantly organic origin (e.g. brain injury, physical health condition, epilepsy) or have a primary diagnosis of substance or alcohol use disorder;
3. cannot understand English sufficiently to engage in conversation without an interpreter;
4. present with a level of risk to others, including the researchers, that cannot be managed feasibility via suitable adjustments, as judged by Chief Investigator

## Recruitment

Principal Investigators will support research assistants to visit clinical services to present the trial, determine initial interest, and distribute information sheets. Referrers will seek consent from potential participants to be contacted by the researchers. Those consenting will be given a participant information sheet and any initial questions will be answered. They will be recontacted after a minimum period of 48 hours, with a longer period if required. Those consenting will be assessed. Those eligible will enter the trial.

For those lacking research capacity, the same initial process will be followed, however in England we will seek the (binding) opinion of the potential participant's Consultee, and in Scotland we will seek consent from the appropriate person (Guardian, Welfare Attorney or nearest relative). In both countries, we will not proceed if there is any indication the potential participant objects to taking part.

We will accept self-referral. A poster will be placed in NHS mental health services to advertise the study. A study website will be available, which will host participant information sheets and contact details for those interested in self-referral. If an individual self-refers, we will only be able to include them in the study if they agree to us contacting a mental health professional involved in their care, to obtain information for risk assessment purposes and to ensure participation is not contraindicated in some way.

Referrers and clinicians will be invited to take part in the Framework analysis by the researchers. Those consenting will be given a participant information sheet and any initial questions will be answered. They will be recontacted after a minimum period of 48 hours, with a longer period if required. Those consenting will be invited to take part in the interviews.

For potential participants entering the study through the clinician-referral recruitment pathway, their clinician will discuss the study with them, answer any questions, give them an information sheet and seek their verbal consent to pass on their details to the research team, and allow them to complete an initial risk assessment. A trained and supervised research assistant (RA) will then contact clinicians to gather contact details for potential participants and complete the risk assessment. The RA will then contact potential participants directly to discuss the study further and answer any questions.

For potential participants entering the study through self-referral, the RA will obtain their explicit verbal consent to contact their keyworker/ care-coordinator to determine whether they meet inclusion criteria for the study and to complete an initial risk assessment. The RA will also ask the clinical care team if they have any concerns about the potential participant's capacity to consent to take part in research. Should participants meet inclusion criteria for the study and continue to express an interest in taking part, the RA will contact them to arrange an appointment. All potential participants will have as long as they wish to consider the information sheet prior to being contacted by the RA, with a minimum period of 48 hours. The information sheet will detail what participants are asked to do, how their information will be used and the possible risks and benefits of taking part in the study.

## Withdrawal/follow-up

### Level of withdrawal

The level of consent withdrawal will be tabulated for each arm of the study. This will be classified as “consent to clinical but not research procedures”, “consent to research procedures but not clinical”, “no clinical or research procedures”, “complete withdrawal as no clinical or research procedures, and removal of consent to use any already collected data”.

### Timing of withdrawal/ lost to follow up data

This will be presented in a CONSORT diagram (see Appendix, Figure 1) with numbers and reasons for withdrawal and/or exclusion from analysis given at each stage (56 days (approximately 8 weeks post-randomisation), 168 days (approximately 24 weeks post-randomisation).

### Presentation of ‘lost to follow up’ data

The numbers (with reasons) of losses to follow-up (drop-outs and withdrawals) over the course of the trial will be summarised overall and by trial arm.

## Baseline patient characteristics

### List of Baseline Characteristics

The list of demographic data which will be reported for participants is:

- Sex (male; female; other),
- Age (years),
- Education (years),
- Employment status (paid or self employment; voluntary employment; sheltered employment; unemployed; student; housewife/husband; retired)
- Marital status (single / married / in a relationship / divorced / widowed / other)
- Residential status (inpatient / rehabilitation ward / supported accommodation /independent living)
- Living arrangements (living alone [+/- children]; living with other relatives; living with husband/wife [+/- children]; living with others; living together as a couple; living with parents; not known)
- Ethnicity (white British; white other; Asian British; Asian other; black British; black other; other),
- Chart diagnosis (schizophrenia; schizoaffective disorder; schizophreniform disorder; delusional disorder; non-affective psychosis, other [to be specified])
- ICD-Code (N with each code)
- Time since first diagnosis (years)
- Duration of untreated psychosis (years)
- Time since first treatment for psychosis from mental health services (years)
- Prescribed antipsychotic medication (yes with N per type; no)
- Prescribed dose of antipsychotic medication (average chlorpromazine equivalents with 95% CIs, excluding those who are antipsychotic-free)
- Recent change in medication (yes with N per type; no)
- Receipt of past psychological therapy (yes with N per type; no)
- Duration of past psychological therapy (average number of weeks with 95% CIs, excluding those who have not received it)
- Legal status: (*Scotland*: voluntary; emergency treatment order; emergency detention certificate; compulsory treatment order; community compulsory treatment order; guardianship; short term detention; *England*: voluntary or informal; community treatment order; section 2; section 3; section 5 [2]; section 5 [4]; other [to be specified])
- Offending history: (yes with N per type; no)
- Number of previous convictions (0; 1; 2; 3; 4; 5; >5)
- Drug and/or alcohol misuse (past only with N per type; current and past with N per type; current only with N per type; no)

The assessments performed at baseline are:

- The Positive and Negative Syndrome Scale (PANSS)
- The Clinical Interview for Psychotic Disorders (CIPD)
- The MacArthur Competence Assessment Tool-Treatment (MacCAT-T)
- The Beads Task
- The Semi-structured Interview Measure of Stigma (SIMS)
- The Calgary Depression Scale (CDS)
- The Brief Neurocognitive Assessment (BNA)
- The Schedule for Assessment of Insight (SAI)^[[2]](#footnote-2)^
- The Rosenberg Self Esteem Scale (RSES)
- The Internalised Stigma of Mental Illness Inventory (ISMI)
- The Questionnaire about the Process of Recovery (QPR)
- The Schizophrenia Quality of Life Scale (SQoL)
- The Clinical Global Impression Scale – Severity – Participant version (CGI-SP)
- The Clinical Global Impression Scale – Severity – Researcher version (CGI-SR)
- The Clinical Global Impression Scale – Capacity (CGI-C)
- The Client Service Receipt Inventory (CSRI)
- The Beck Anxiety Inventory (BAI)
- The Brief Core Schema Scale (BCSS)

### Methods to Summarise Baseline Characteristics

All baseline figures will be reported for the sample as a whole and per arm of each trial. All quantitative participant baseline characteristics will be summarised by numbers and percentages, or mean (standard deviation; SD) / median (interquartile range; IQR), as appropriate.

Performance on the Beads Task at baseline will be reported as (i) average number of beads taken before a decision (mean & SD or median & IQR), and (ii) numbers and percentage making decisions based on 2 or fewer beads. Assessment scores for other measures will be summarised by mean total and/or subscale scores (where applicable) with standard deviation (or median & IQR). In addition, the following numbers and percentages will be reported, representing:

- Those with impairment on the Understanding, Reasoning, Appreciation or Expressing a choice domains of the MacCAT-T, as determined by the researcher.
- Those with impairment on 1, 2, 3 or 4 domains of the MacCAT-T, as determined by the researcher
- Those with a score of <15 on the RSES, signifying low-self-esteem
- Those with a score of ≥60 on the ISMI, signifying high self-stigma
- Those with scores of 1, 2, 3, 4, 5, 6 or 7 on the CGI-SP
- Those with scores of 1, 2, 3, 4, 5, 6 or 7 on the CGI-SR
- Those with scores of 1, 2, 3, 4, 5, 6 or 7 on the CGI-C
- Those with adjusted PANSS total scores (scored 0-180 rather than 30-210) of 0-27 (minimal or absent illness), 28-44 (mildy ill), 45-64 (moderately ill), 65-85 (markedly ill) and ≥86 (severely ill).
- Those with BAI total scores of 0–7 (minimal or absent anxiety), 8–15 (mild anxiety), 16–25 (moderate anxiety), and 26–63 (severe anxiety)
- Those with CDSS total scores of 0-6 (minimal or absent symptoms of depression) and 7-27 (possible major depressive episode)

# Analysis

Date will be reported in line with the Consolidated Standards of Reporting Trials (CONSORT) 2010 Statement, including the extensions to randomised pilot and feasibility trials (4), reporting of harms (5), and reporting of psychological and social interventions (6).

## Primary analyses

The specific listed research questions will be addressed as follows:

a) Research Question 1: What data quality and completion rates can be obtained for the main outcome measure?

**Outcomes**

This primary outcome refers to data completion rates at 8-weeks post-randomisation (end of treatment) on the MacCAT-T (anticipated primary outcome for a future trial). The MacCAT-T assesses participants on 4 domains: Understanding scored 0-6 (3 items); (ii) Reasoning scored 0-8 (4 items); (iii) Appreciation scored 0-4 (2 items); and (iv) Expressing a choice scored 0-2 (1 items). Higher scores indicate greater current ability in each domain. Data completion here refers to the number of participants completing MacCAT-T assessments at week 8 divided by the number of participants randomised to treatment or control.

**Analysis**

Data will be presented as a percentage with 95% CIs. Estimates will be presented for the overall study, per trial, and per arm of each trial.

b) Research Question 2: What proportion of eligible patients consent, engage, & complete the trial?

**Outcomes**

This includes our primary outcome, which relates to achieving the recruitment target (N=60). This will be defined as the overall number of participants recruited and randomised during the recruitment window, divided by the recruitment target. Our participant flow diagram (i.e., CONSORT diagram with extension for feasibility / pilot trials) will also report the (i) number of potentially eligible and eligible patients we identified; (ii) the number of eligible patients who consented to take part; (iii) the number of eligible patients who were randomised to treatment or control; (iv) the number of participants randomised to treatment or control who received at least 3 hours of the clinical procedures.

**Analysis**

The overall number of participants recruited and randomised during the recruitment window, divided by the recruitment target, will be reported as a percentage with 95% CIs. All other participant flow information will be reported in line with CONSORT requirements.

c) Research Question 3: What do participants, clinicians & collaborators think will improve study processes, including interventions?

**Outcomes**

Six patient participants and 6 clinical or social care staff who have either referred a participant to the study or have provided substantial care and treatment to a participant during the study will be invited to attend a qualitative interview to understand their experiences of the trial and to identify any changes they suggest.

**Analysis**

Framework analysis will be used to identify and synthesise the converging and diverging information that arise from interview data, highlighting relationships between different parts of the data. It will be used to derive descriptive and/or explanatory conclusions around themes.

d) Research Question 4: Are trial procedures acceptable to participants and clinicians, and feasible to implement?

**Outcomes**

Six patient participants and 6 clinical or social care staff who have either referred a participant to the study or have provided substantial care and treatment to a participant during the study will be invited to attend a qualitative interview to understand their experiences of the trial and to identify any changes they suggest.

**Analysis**

Framework analysis will be used to identify and synthesise the converging and diverging information that arise from interview data, highlighting relationships between different parts of the data. It will be used to derive descriptive and/or explanatory conclusions around themes.

e) Research Question 5: What sample size is required in a full study?

**Outcomes**

The planned primary outcome for a full study is the MacCAT-T score at post-treatment (8 weeks).

**Analysis**

Key summary statistics will be estimated (e.g. pooled within-group SD, baseline-outcome correlation coefficient) to inform calculation of the sample size required for a full efficacy trial based on this outcome at this time-point.

## Secondary analyses

The following secondary analyses will be conducted:

a) What data quality and completion rates can be obtained for the main outcome measure at follow-up?

**Outcomes**

This outcome refers to data completion rates at 24-weeks post-randomisation (follow-up) on the MacCAT-T. Data completion here refers to the number of participants completing MacCAT-T assessments at week 24 divided by the number of participants randomised to treatment or control AND eligible to receive a follow-up assessment^[[3]](#footnote-3)^

**Analysis**

Data will be presented as a percentage with 95% CIs. Estimates will be presented for the overall study, per trial, and per arm of each trial.

b) What data quality and completion rates can be obtained for our measures of targeted psychological mechanisms (self-esteem, self-stigma and JTC bias)?

**Outcomes**

This outcome refers to data completion rates at 0, 8 and 24-weeks on the Rosenberg Self-Esteem Scale (RSES; self-esteem), the Semi-Structured Interview Measure of Stigma (SIMS; self-stigma) and the Beads Task (JTC bias). Data completion here refers to the number of participants completing RSES, SIMS and Beads Task assessments at (i) 0 weeks, (ii) 8 weeks and (iii) 24 weeks divided by the number of participants randomised to treatment or control AND participating in the trial of interventions which target the mechanism in question. At 24 weeks, the denominator will be those who are eligible to receive a follow-up assessment. The RSES and SIMS each have a minimum score of 0 and a maximum score of 30 (RSES; higher scores indicate greater self-esteem) and 40 (SIMS; higher scores indicates greater self-stigma). Performance on the Beads Task is measured as the number of beads taken before a decision, with more beads indicating greater data-gathering.

**Analysis**

Data will be presented as a percentage with 95% CIs. Estimates will be presented for the overall study, per trial, and per arm of each trial at week 0, and per trial and arm of each trial at weeks 8 and 24.

c) Is there any evidence that clinical and research procedures might be associated with an increased number of deaths by suicide?

**Outcomes**

The number of deaths due to suicide occurring in each treatment and control group by week 8 and week 24 will be reported as part of the trial adverse event monitoring and reporting protocol. We will also report whether the death was judged by an independent clinical expert, Sponsor and/or NHS REC to be causally related to research and/or clinical procedures.

**Analysis**

The number of deaths by suicide will be presented for the overall study, per trial, and per arm of each trial at weeks 8 and 24, together with narrative summary of judgements of likely causality.

d) Is there any evidence that clinical and research procedures might be associated with an increased number of suicide attempts?

**Outcomes**

The number of participants attempting suicide in each treatment and control group by week 8 and week 24 will be reported as part of the trial adverse event monitoring and reporting protocol. We will also report whether the attempt was judged by an independent clinical expert, Sponsor and/or NHS REC to be causally related to research and/or clinical procedures.

**Analysis**

The number of participants attempting suicide will be presented for the overall study, per trial, and per arm of each trial at weeks 8 and 24, together with narrative summary of judgements of likely causality.^[[4]](#footnote-4)^

e) Is there any evidence that clinical and research procedures might be associated with an increased number of participants experiencing suicidal crisis without suicide attempt?

**Outcomes**

The number of participants experiencing suicidal crisis without attempting suicide and the overall number of suicidal crises occurring in each treatment and control group at weeks 0, 8 and week 24 will be reported as part of the trial adverse event monitoring and reporting protocol. Suicidal crisis without attempt is defined as a score of 2 on item 8 of the Calgary Depression Rating Scale for Schizophrenia (CDSS). Item 8 of the CDSS is scored 0 (absent) to 3 (severe), with higher scores indicating greater severity. We will also report whether the event was judged by an independent clinical expert, Sponsor and/or NHS REC to be causally related to research and/or clinical procedures

**Analysis**

The number of participants experiencing suicidal crises will be presented for the overall study, per trial, and per arm of each trial at weeks 0, 8 and 24, together with narrative summary of judgements of likely causality.^[[5]](#footnote-5)^

f) Is there any evidence that clinical and research procedures might be associated with an increased number of deaths not related to suicide?

**Outcomes**

The number of deaths not related to suicide occuring in each treatment and control group by week 8 and week 24 will be reported as part of the trial adverse event monitoring and reporting protocol. We will also report whether the death was judged by an independent clinical expert, Sponsor and/or NHS REC to be causally related to research and/or clinical procedures.

**Analysis**

The number of deaths not related to suicide will be presented for the overall study, per trial, and per arm of each trial at weeks 8 and 24, together with narrative summary of judgements of likely causality.

g) Is there any evidence that clinical and research procedures might be associated with an increased number of participants experiencing severe symptom exacerbation?

**Outcomes**

The number of participants experiencing severe symptom exacerbation in each treatment and control group by week 8 and week 24 will be reported as part of the trial adverse event monitoring and reporting protocol. Severe symptom exacerbation is defined as a rating of ≥6 on the patient or researcher-rated Clinical Global Impression Severity (CGI-S) and Clinical Global Impression Improvement (CGI-I) scales. Both the patient and researcher-rated CGI-S are scored from 1 to 7, with higher scores indicating greater symptom severity. Both the patient and researcher-rated CGI-I scales are scored from 1 to 7, with higher scores indicating less improvement. We will also report whether the severe symptom exacerbation was judged by an independent clinical expert, Sponsor and/or NHS REC to be causally related to research and/or clinical procedures.

**Analysis**

The number of participants experiencing severe symptom exacerbation will be presented for the overall study, per trial, and per arm of each trial at weeks 8 and 24, together with narrative summary of judgements of likely causality.^[[6]](#footnote-6)^

h) Is there any evidence that clinical and research procedures might be associated with an increased number of participants reporting mild to moderate adverse events?

**Outcomes**

The number of participants stating they agree 'quite a lot' or 'very much' (corresponding to a score of 3 or 4 respectively) with each item on a self-report measure of adverse events (the Adverse Experiences in Psychotherapy questionnaire; AEP) at weeks 8 and 24 will be reported as part of the trial adverse event monitoring and reporting protocol. The AEP contains 28-items, 21 of which attempt to measure potential adverse effects of trial participation as determined by the individual (e.g., *'Taking part has made me feel more anxious'*). Four items attempt to measure acceptability (e.g., “*Taking part involved too much hard work*”) and two item attempt to measure perceived need for care following participation ( “*My problems have improved to the point whereby I no longer feel I need help*”; “*Taking part hasn’t helped me with my problems*.”). Participants are also invited to describe their experience of taking part in the study in your own words. Participants who leave the study early are invited to complete a parallel version of the AEP, designed to assess whether their early discontinuation was a consequence of (an) adverse event(s).

**Analysis**

The number of participants reporting scores of 3 or 4 for each item on each version of the AEP will be presented for the overall study, per trial, and per arm of each trial at weeks 8 and 24.

i) What data quality and completion rates can be obtained for our measure of depression?

**Outcomes**

This outcome refers to data completion rates at 0, 8 and 24-weeks on the Calgary Depression Scale for Schizophrenia (CDSS). Data completion here refers to the number of participants completing the CDSS at (i) 0 weeks, (ii) 8 weeks and (iii) 24 weeks divided by the number of participants randomised to treatment or control. At 24 weeks, the denominator will be those who are eligible to receive a follow-up assessment. The CDSS is a 9-item interview-based measure and has a minimum score of 0 and a maximum score of 27, with higher scores indicating more severe depression.

**Analysis**

Data will be presented as a percentage with 95% CIs. Estimates will be presented for the overall study, per trial, and per arm of each trial.

j) What data quality and completion rates can be obtained for our measure of anxiety?

**Outcomes**

This outcome refers to data completion rates at 0, 8 and 24-weeks on the Beck Anxiety Inventory (BAI). Data completion here refers to the number of participants completing the BAI at (i) 0 weeks, (ii) 8 weeks and (iii) 24 weeks divided by the number of participants randomised to treatment or control. At 24 weeks, the denominator will be those who are eligible to receive a follow-up assessment. The BAI is a 21-item questionnaire and has a minimum score of 0 and a maximum score of 63, with higher scores indicating more severe anxiety.

**Analysis**

Data will be presented as a percentage with 95% CIs. Estimates will be presented for the overall study, per trial, and per arm of each trial.

k) What data quality and completion rates can be obtained for our measure of quality of life?

**Outcomes**

This outcome refers to data completion rates at 0, 8 and 24-weeks on the Schizophrenia Quality of Life Scale (SQoLS). Data completion here refers to the number of participants completing the SQoLS at (i) 0 weeks, (ii) 8 weeks and (iii) 24 weeks divided by the number of participants randomised to treatment or control. At 24 weeks, the denominator will be those who are eligible to receive a follow-up assessment. The SQoLS consists of 30 items on 3 sub-scales: (i) Psychosocial (15 items); (ii) Motivation and energy (7 items); and (iii) Symptoms and side effects (8 items). Scores are transformed per scale to have a range from 0-100, with higher scores indicating poorer quality of life.

**Analysis**

Data will be presented as a percentage with 95% CIs. Estimates will be presented for the overall study, per trial, and per arm of each trial.

l) What data quality and completion rates can be obtained for our measure of recovery?

**Outcomes**

This outcome refers to data completion rates at 0, 8 and 24-weeks on the Questionnaire about the Process of Recovery (QPR). Data completion here refers to the number of participants completing the QPR at (i) 0 weeks, (ii) 8 weeks and (iii) 24 weeks divided by the number of participants randomised to treatment or control. At 24 weeks, the denominator will be those who are eligible to receive a follow-up assessment. The scale consists of 15 items and scores range from 0-30. Higher scores are indicative of greater subjective recovery.

**Analysis**

Data will be presented as a percentage with 95% CIs. Estimates will be presented for the overall study, per trial, and per arm of each trial

m) What data quality and completion rates can be obtained for our measure of core schema?

**Outcomes**

This outcome refers to data completion rates at 0, 8 and 24-weeks on the Brief Core Schema Scale (BCSS). Data completion here refers to the number of participants completing the BCSS at (i) 0 weeks, (ii) 8 weeks and (iii) 24 weeks divided by the number of participants randomised to treatment or control. At 24 weeks, the denominator will be those who are eligible to receive a follow-up assessment. There are four subscales, each of which contains 6 items, has a minimum score of 0 and a maximum score of 24; (i) negative beliefs about the self; (ii) negative beliefs about others; (iii) positive beliefs about the self; and (iv) positive beliefs about other. Participants first indicate whether they hold a particular belief. If they do, they indicate on a scale from 1-4 how strongly they believe it.

**Analysis**

Data will be presented as a percentage with 95% CIs. Estimates will be presented for the overall study, per trial, and per arm of each trial.

n) What data quality and completion rates can be obtained for our measure of psychotic symptoms?

**Outcomes**

This outcome refers to data completion rates at 0, 8 and 24-weeks on the Positive and Negative Syndrome Scale (PANSS). Data completion here refers to the number of participants completing the PANSS at (i) 0 weeks, (ii) 8 weeks and (iii) 24 weeks divided by the number of participants randomised to treatment or control. At 24 weeks, the denominator will be those who are eligible to receive a follow-up assessment. The subscales of this interview measure are: (i) Positive symptoms (6 items); (ii) Negative symptoms (8 items); (iii) Excitement (4 items); (iv) Emotional distress (5 items); (v) Disorganised (7 items). The total score across the 30 items ranges from 30-210, however an adjusted scoring system (0-180) will be used in the current trial to allow calculation of those experiencing 0, 25%, 50%, 75% and 100% improvement. Higher scores indicate more severe symptomatology on all scales.

**Analysis**

Data will be presented as a percentage with 95% CIs. Estimates will be presented for the overall study, per trial, and per arm of each trial.

o) What data quality and completion rates can be obtained for our measure of service use?

**Outcomes**

This outcome refers to data completion rates at 0, 8 and 24-weeks on the Client Service Receipt Inventory (CSRI). Data completion here refers to the number of participants completing the CSRI at (i) 0 weeks, (ii) 8 weeks and (iii) 24 weeks divided by the number of participants randomised to treatment or control. At 24 weeks, the denominator will be those who are eligible to receive a follow-up assessment. Sections include information on number of inpatient days, number of contacts with outpatient services, medication & dose, criminal justice contacts, type and frequency of engagement with primary & community care contacts.

**Analysis**

Data will be presented as a percentage with 95% CIs. Estimates will be presented for the overall study, per trial, and per arm of each trial.

p) Is rater masking feasible in this trial?

**Outcomes**

All instances of outcome assessors (i.e., raters) becoming aware of whether a participant has been randomised to treatment or control (i.e., a blind break) will be recorded and reported to investigate feasibility of rater masking.

**Analysis**

The number of blind-breaks will be presented for the overall study, per trial, and per arm of each trial.

q) To what extent does improvement in appreciation ratings on the MacArthur Competence Assessment Tool for Treatment (MacCAT-T) reflect genuine improvement?

**Outcomes**

Up to 10 participants with improved MacCAT-T Appreciation subscale scores will be interviewed using qualitative 'case study' methodology to investigate the clinical validity of this change. The MacCAT-T Appreciation subscale is scored 0-4 (2 items), with higher scores indicating better appreciation.

**Analysis**

Analysis of interviews and research data will be structured using Yin’s (2014) overall ‘explanation building’ framework (3). The steps involve making an initial explanatory proposition and comparing the findings of an initial case against this proposition. This is then revised, and other details of the case and any additional cases are compared against the revision. This process is repeated as many times as needed.

r) To what extent do appreciation ratings on the MacArthur Competence Assessment Tool for Treatment (MacCAT-T) correspond with measures of insight?

**Outcomes**

MacCAT-T Appreciation ratings will be compared to ratings on the Schedule for the Assessment of Insight (SAI)^[[7]](#footnote-7)^.The MacCAT-T Appreciation subscale is scored 0-4 (2 items), with higher scores indicating better appreciation. The SAI is scored from 0 to 14, with higher scores consistent with greater insight.

**Analysis**

Our default approach will be to assess concordance between all SAI ratings of insight and MacCAT-T judgements of appreciation using a polyserial correlation, estimated between MacCAT-T judgement of appreciation and SAI scores. These will be performed independently on baseline, end-of-treatment and follow-up data, and each result will be reported with 95% CIs. Alternative methods may be used depending on whether the data meets distributional assumptions.

s) To what extent do overall ratings on the MacArthur Competence Assessment Tool for Treatment (MacCAT-T) correspond to clinician ratings of capacity?

**Outcomes**

Participants' clinicians will be asked to complete a version of the Clinical Global Impression Scale (CGI-capacity) at weeks 0 and 8, modified to assess treatment decision-making capacity. Concordance between CGI-capacity and MacCAT-T judgements of capacity will be assessed. The CGI-capacity is scored from 1 to 7, with higher scores indicating more severe decision-making incapacity. The MacCAT-T assesses participants on 4 domains: (i) Understanding scored 0-6 (3 items); (ii) Reasoning scored 0-8 (4 items); (iii) Appreciation scored 0-4 (2 items); and (iv) Expressing a choice scored 0-2 (1 items). Higher scores indicate greater current ability in each domain.

**Analysis**

Our default approach will be to assess concordance between CGI-capacity ratings and each of the 4 domains of MacCAT-T ratings of capacity using polychoric correlations, and concordance between CGI-capacity ratings and total MacCAT-T scores using polyserial correlations. These will be performed independently on baseline, end-of-treatment and follow-up data, and each result will be reported with 95% CIs. Alternative methods may be used depending on whether the data meets distributional assumptions.

### Methods used to check assumptions of statistical methods

The assumptions of normality for each outcome at each time-point per arm of each trial will be examined through summary statistics including the mean, median, standard deviation, skewness and kurtosis, as well as through histograms and normal probability plots of residuals.

### Details of alternative methods to be used if distributional assumptions do not hold

In the case of non-normal data, bootstrap methods will be applied where feasible (7).

### Missing data

Total scores for the various assessment measure tools and their subscales collected at baseline and outcome time-points will be computed providing no more than 25% of items are missing; otherwise the total score will be recorded as missing, but not the subscale score if ≤25% items are missing from that. If subscale and total scores have >0 to ≤25% missing items, the missing items will be imputed using the mean score of the other items contributing to that score.

### Sensitivity analyses

None planned.

### Subgroup analyses

None planned.

### Additional analyses

### Analyses of planned primary outcome

The first set of these analyses relates to the planned primary outcome for a definitive trial, the MacCAT-T. These will be performed on datasets incorporating all those randomised within each trial (intention-to-treat sample; ITT) who provided complete data on that outcome (observed cases; OC), regardless of intervention uptake. In small samples, analyses of observed cases has been shown to perform at least as well as maximum-likelhood estimation, fully conditional specification multiple imputation and joint multiple imputation in relation to type 1 error, power and degree of bias in regression coefficients, particularly when missing data is 20% or less and satisfies the Missing At Random assumption (8).

Sensitivity analyses will be performed to explore the effect of restricting the dataset to all per-protocol (PP) observed cases, consisting of all participants who were randomised, received at least 3 hours of clinical contact with the therapist and provided usable data. All analytical decisions will be made by a researcher masked to which group is treatment or control.

The following will be produced and reported for each trial:

- Means and SDs for overall MacCAT-T score at EoT and FU for treatment and control groups
- Means and SDs for the ‘appreciation’ subscale at EoT and FU for treatment and control groups
- Means and SDs for the ‘understanding’ subscale at EoT and FU for treatment and control groups
- Means and SDs for the ‘reasoning’ subscale at EoT and FU for treatment and control groups
- Means and SDs for the ‘communicating’ subscale at EoT and FU for treatment and control groups
- Unstandardised and standardised differences in group means in overall MacCAT-T scores at EoT and FU with 95% CI
- Unstandardised and standardised differences in group means in appreciation scores at EoT and FU with 95% CI
- Unstandardised and standardised differences in group means in understanding scores at EoT and FU with 95% CI
- Unstandardised and standardised differences in group means in reasoning scores at EoT and FU with 95% CI
- Unstandardised and standardised differences in group means in communication scores at EoT and FU with 95% CI

Effect sizes will be derived from individual linear regressions, incorporating treatment group and baseline values as predictor variables. Standardised mean differences and CIs for each trial will be calculated by dividing the unstandardised regression coefficients and corresponding CIs by the pooled SD of the baseline values. A Hedges’s g adjustment will be applied to account for the small sample sizes. Estimates based on data which deviates from the distributional assumptions of linear regression will be flagged, bootstrapped and supplemented with the median and interquartile range.

### Analyses of other outcomes

Analyses of planned secondary and safety outcomes will also be restricted to the ITT:OC sample. No multiple imputation will be performed. For each continuous outcome we will report:

- Means and SDs for each total and subscale score at EoT and FU for treatment and control groups
- Unstandardised and standardised differences in group means for each total and subscale score at EoT and FU with 95% CI

Effect sizes will be derived from individual linear regressions, incorporating group and baseline values of the outcome as predictors. Standardised mean differences and CIs for each trial will be calculated by dividing the unstandardised regression coefficients and corresponding CIs by the pooled SD of the baseline values. A Hedges’s g adjustment will be applied to account for the small sample sizes. Estimates for non-normally distributed outcomes will be flagged, boostrapped and supplemented with the median and interquartile range.

Event data will be presented as a count and as a percentage, with the ITT sample N and number of observed events serving as default values for the denominator and numerator, respectively. This assumes events, whether beneficial or adverse, have not occurred if missing, however readers can easily test the effect of different assumptions. We will report the corresponding relative and absolute risks of benefit or harm, together with numbers needed to treat for both, and 95% CIs for all estimates.

### Software

SPSS and Stata will be used to manage and analyse quantitative data.

# References

1. Grisso T, Appelbaum PS. MacArthur Competence Assessment Tool for Treatment (MacCAT-T). Professional Resource Press/Professional Resource Exchange; 1998.

2. Gale NK, Heath G, Cameron E, Rashid S, Redwood S. Using the framework method for the analysis of qualitative data in multi-disciplinary health research. BMC medical research methodology. 2013;13(1):1–8.

3. Yin RK. Case Study Research and Applications: Design and Methods. 5th ed. Thousand Oaks, California: SAGE Publications Inc.; 2014.

4. Eldridge SM, Chan CL, Campbell MJ, Bond CM, Hopewell S, Thabane L, et al. CONSORT 2010 statement: extension to randomised pilot and feasibility trials. BMJ. 2016;355.

5. Ioannidis JP, Evans SJ, Gøtzsche PC, O’neill RT, Altman DG, Schulz K, et al. Better reporting of harms in randomized trials: an extension of the CONSORT statement. Annals of internal medicine. 2004;141(10):781–8.

6. Montgomery P, Grant S, Mayo-Wilson E, Macdonald G, Michie S, Hopewell S, et al. Reporting randomised trials of social and psychological interventions: the CONSORT-SPI 2018 Extension. Trials. 2018;19(1):1–14.

7. Pek J, Wong O, Wong AC. How to address non-normality: A taxonomy of approaches, reviewed, and illustrated. Frontiers in psychology. 2018;9:2104.

8. McNeish D. Missing data methods for arbitrary missingness with small samples. Journal of Applied Statistics. 2017;44(1):24–39.

#
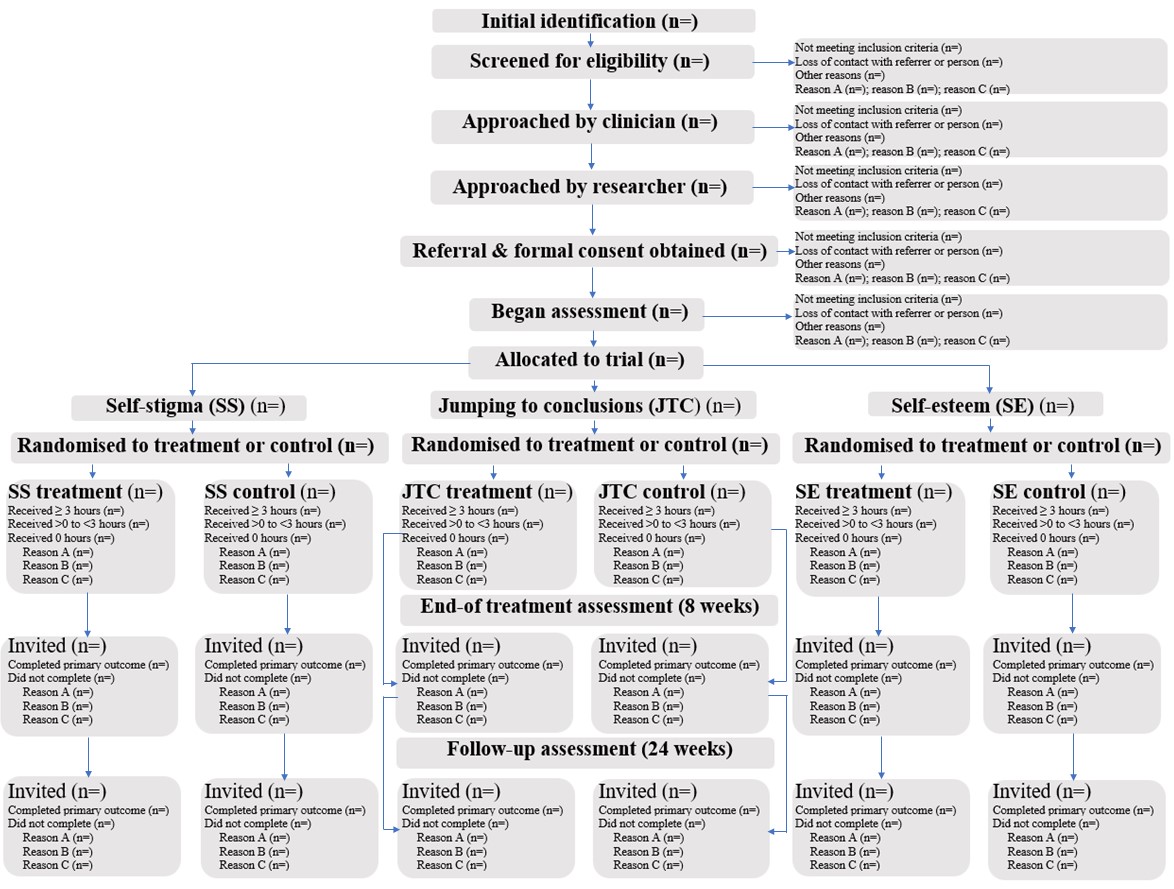
Appendix

**Figure 1:** CONSORT Diagram

1. <https://www.sealedenvelope.com/> [↑](#footnote-ref-1)
2. We intended to use the Expanded Schedule for the Assessment of Insight, however an error led to the original version being used instead. [↑](#footnote-ref-2)
3. In the original (pre-pandemic) protocol, we stated *“only those randomised in the first 5 (England) to 8 (Scotland) months will be eligible for follow-up assessment”.* The trial was then extended to mitigate the effect of the pandemic on recruitment. We acquired resources to complete follow-up assessments until the last scheduled end-of-treatment assessments were completed, however we lacked resources to continue them when those end-of-treatment assessments were delayed due to the effects of the pandemic on clinical staffing. The net effect of this was that *only those randomised in the first 5 (England) to 23 (Scotland) months were eligible for follow-up assessment.*. [↑](#footnote-ref-3)
4. We will report which events were identified via research assistants who were blind to treatment arm allocation (assessed at weeks 0, 8 and 24), and which events were identified via clinicians who were not blind to allocation (assessed at start of each clinical session). [↑](#footnote-ref-4)
5. We will report which events were identified via research assistants who were blind to treatment arm allocation (assessed at weeks 0, 8 and 24), and which events were identified via clinicians who were not blind to allocation (assessed at start of each clinical session). [↑](#footnote-ref-5)
6. We will report which events were identified via research assistants who were blind to treatment arm allocation (assessed at weeks 0, 8 and 24), and which events were identified via clinicians who were not blind to allocation (assessed at start of each clinical session). [↑](#footnote-ref-6)
7. We intended to use the Expanded Schedule for the Assessment of Insight, however an error led to the original version being used instead. [↑](#footnote-ref-7)
