# Supplementary material for: Accelerating the development of a psychological intervention to restore treatment decision-making capacity in patients with schizophrenia-spectrum disorder: a study protocol for a multi-site, assessor-blinded, pilot Umbrella trial (the DEC:IDES trial)
Source: Pilot Feasibility Stud. 2023 Jul 8;9:117. doi: 10.1186/s40814-023-01323-0 (PMC10329297; doi:10.1186/s40814-023-01323-0)
Supplement: Supplementary file 2 — Additional file 2: Supplementary Appendix. [file 40814_2023_1323_MOESM2_ESM.docx]

**Supplementary appendix to:**

Hutton, P., Kelly, J., Taylor, C. D. J., Williams, B., Emsley, R., Ho, C., Vikram, A., Saddington, D., McCann, A., Burke, J., Eliasson, E., Harper, S., Karatzias, T., Taylor, P. J. T., Watson, A., Dougall, N., Stavert, J., O’Rourke, S., Glasgow, A., Murphy, R., Palmer, K., Zaidi, N., Bidwell, P., Pritchard, J., Carr, L., Woodrow, A. (submitted). Accelerating the development of a psychological intervention to restore treatment decision-making capacity in patients with schizophrenia-spectrum disorder: study protocol for a multi-site, assessor-blinded, pilot Umbrella trial (the DEC:IDES trial).

**Contents**

[1. Sponsor contact details 3](#_Toc129376852)

[2. World Health Organisation trial registration data set 4](#_Toc129376853)

[3. Protocol versions 6](#_Toc129376854)

[4. Data management plan (version 2) 7](#_Toc129376855)

[5. Trial Steering Committee terms of reference (version 2) 13](#_Toc129376856)

[6. Combined reporting checklist 16](#_Toc129376857)

[7. CONSERVE Checklist 34](#_Toc129376858)

[8. Recruitment poster – participants (version 2) 36](#_Toc129376859)

[9. Participant information sheet (version 4) 37](#_Toc129376860)

[10. Referrer information sheet (version 2) 44](#_Toc129376861)

[11. Information sheet for Welfare Attorney, Welfare Guardian or nearest relative (Scotland) (version 5) 50](#_Toc129376862)

[12. Information sheet for Consultee (England) (version 4) 57](#_Toc129376863)

[13. Consent form for Welfare Attorney, Welfare Guardian or nearest relative (Scotland) (version 2) 64](#_Toc129376864)

[14. Consultee Declaration Form (England) (version 1) 66](#_Toc129376865)

[15. Information sheet for clinicians participating in qualitative study (version 1) 67](#_Toc129376866)

[16. Participant consent form (version 1) 72](#_Toc129376867)

[17. Consent form for clinicians participating in qualitative study (version 1) 73](#_Toc129376868)

1. Sponsor contact details

| **Trial Sponsor**: | Edinburgh Napier University |
| --- | --- |
| **Sponsor’s Reference:** | 1206004 |
| **Contact name**: | Paula Stevenson |
| **Address**: | Research and Innovation Office, Edinburgh Napier University, 9 Sighthill Court Edinburgh EH11 4BN |
| **Telephone**: | 0131 455 6009 |
| **Email**: | [researchintegrity@napier.ac.uk](mailto:researchintegrity@napier.ac.uk) |

2. World Health Organisation trial registration data set

| **Data category** | **Information** |
| --- | --- |
| Primary registry and trial identifying number | ClinicalTrials.gov NCT04309435 |
| Date of registration in primary registry | 16 March, 2020 |
| Secondary identifying numbers | IRAS: 263575 & 265638  Scotland A REC: 19/SS/0069  Wales REC 5: 19/WA/0155  Funder: HIPS/18/60  Sponsor: 1206004 |
| Source(s) of monetary or material support | Chief Scientist Office, Scotland |
| Primary sponsor | Edinburgh Napier University |
| Secondary sponsor(s) | NA |
| Contact for public queries | Professor Paul Hutton [0131 455 3555] [p.hutton@napier.ac.uk] |
| Contact for scientific queries | Professor Paul Hutton [0131 455 3555] [p.hutton@napier.ac.uk] |
| Public title | DEcision-making Capacity: Intervention Development & Evaluation in Schizophrenia-spectrum disorders (DEC:IDES) |
| Scientific title | DEcision-making Capacity: Intervention Development & Evaluation in Schizophrenia-spectrum disorders (DEC:IDES): a multi-site, assessor-blinded, pilot Umbrella trial |
| Countries of recruitment | Scotland, England |
| Health condition(s) or problem(s) studied | Impaired treatment decision-making capacity |
| Intervention(s) | Active comparators:  Psychological intervention for self-stigma  Psychological intervention for low self-esteem  Psychological intervention for jumping-to-conclusions bias Placebo comparator: Further assessment of factors affecting treatment decision-making capacity |
| Key inclusion and exclusion criteria | Ages eligible for study: ≥18-65 years Sexes eligible for study: both Accepts healthy volunteers: no Inclusion criteria: diagnosed with schizophrenia-spectrum disorder (schizophrenia, schizoaffective disorder, delusional disorder, psychosis not otherwise specified, brief psychotic disorder); aged 18-65; judged to lack capacity to make treatment decisions by their referring clinician and the researcher (using the MacCAT-T); have either low self-esteem, defined as a score of <15 on the Rosenberg Self-Esteem Scale (RSES), high self-stigma, defined as a score of ≥60 on Internalised Stigma of Mental Illness Inventory (ISMI) and/or a JTC bias, defined as selecting ≤2 beads on the Beads Task; able to be interviewed and complete the measures; be registered as a patient with clinical or social care services. Exclusion criteria: presence of a moderate to severe learning disability; psychosis of a predominantly organic origin (e.g. brain injury, physical health condition, epilepsy); primary diagnosis of substance or alcohol use disorder; cannot understand English sufficiently to engage in conversation without an interpreter; present with a level of risk to others that cannot be managed via suitable adjustments. |
| Study type | Interventional Allocation: randomized Intervention model: parallel assignment Masking: single blind (outcomes assessor) Primary purpose: improvement of treatment decision-making capacity Feasibility / pilot |
| Date of first enrolment | January 2021 |
| Target sample size | 60 |
| Recruitment status | Not recruiting |
| Primary outcome(s) | Feasibility of recruitment and data retention on the MacArthur Competence Assessment Tool-Treatment at end-of-treatment (8 weeks). |
| Key secondary outcomes | (1) Data completion rates on the MacCAT-T at 24-weeks and data completion at 8 and 24 weeks on the Positive And Negative Syndrome Scale, the Questionnaire on the Process of Recovery, the Schizophrenia Quality of Life scale, the Client Service Receipt Inventory, the Beck Anxiety Inventory, the Brief Core Schema Scale, the Calgary Depression Scale for Schizophrenia, the Rosenberg Self-esteem Scale, the Beads Task (85:15 version) and the Structured Interview Measure of Stigma. (2) Adverse events. (3) MacCAT-T construct validity |

3. Protocol versions

| Date | Description | Version No. |
| --- | --- | --- |
| 10/7/2019 | Original | 3 |
| 17/02/2020 | Amendment SCO1 (17/02/2020): Rosenberg Self Esteem Scale replaced the Robson Self Concept Questionnaire. Clinical Interview for Psychotic Disorders (CIPD) replaced the Structured Clinical Interview for DSM-V. Addition of Brief Core Schema Scale. Addition of Client Service Receipt Inventory. Randomisation sequence parameters changed.  Amendment ENG1 (20/10/2020): Primary reason for amendment: To align English site documentation with that of the Scottish site (IRAS ID 263575), a statement has been added to the information sheets and recruitment posters to explain the status of the study as a 'feasibility trial' being run to determine if it will be possible to take the research on to a larger main trial. A further statement was added to make clear to participants that although they may self-refer or be referred by clinicians into the trial, they may not meet all eligibility criteria and therefore may not be able to take part. | 4 |
| 1/7/2021 | Amendments ENG3 & SCO4: Primary reason for amendments: Extension to study duration to mitigate effects of pandemic. Replacement of independent statistician with analysis by Dr Peter Taylor to save costs, to mitigate effects of pandemic. Removal of unnecessary CIPD assessments at weeks 8 and 24. | 5 |
| 8/6/2022 | Amendments ENG4 & SCO6: Primary reason for amendments: Extension to study duration to mitigate effects of pandemic. | 6 |
| 23/8/2022 | Amendment ENG5^[[1]](#footnote-1)^: Primary reason for amendment: Self-stigma and JTC trials reopened. Preferential allocation of new participants to the self-esteem trial. Participants who have completed one trial allowed to take part in another, if eligible for it. | 7 |
| 27/9/2022 | Amendment ENG6: Primary reason for amendment: To formally allow randomisations to be performed prior to session 1 if randomisation administrator not available at the time of session 1. | 8 |
| 06/12/2022 | Amendment ENG7: Primary reason for amendment: To extend the formal end date of the project in all sites from 31/12/22 until 30/6/23. | 9 |

4. Data management plan (version 2)


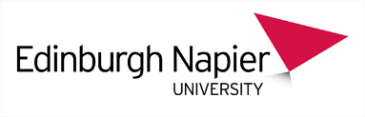


Logo of host NHS organisation to be inserted here


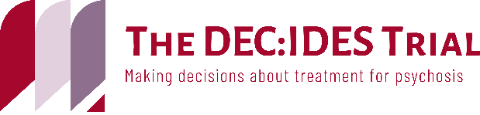


**Data Management Plan**

**DE**cision-making **C**apacity: **I**ntervention **D**evelopment & **E**valuation in **S**chizophrenia-spectrum disorder: The DEC:IDES Trial

| **0. Proposal name** | |
| --- | --- |
| **DEcision-making Capacity: Intervention Development & Evaluation in Schizophrenia-spectrum disorder: The DEC:IDES Trial** | |
| **1. Description of the data** | |
| **1.1 Type of study**  The main aim of this study is to find out whether people with psychosis will take part in an ‘Umbrella trial’ of talking therapies to improve their treatment decision-making capacity (the DEC:IDES trial). We want to understand their experiences of participation, and we also want to understand the experiences of clinicians who have referred their patients to this study. In particular, we need to find out whether participants will stay in DEC:IDES until it is finished, or whether they will leave early. We also need to understand why people might leave DEC:IDES early, so that we can improve it. For these reasons, we are running a smaller version first. This will involve **3 small randomised controlled trials**, each testing 1 of 3 different interventions. Each intervention has been designed to help participants resolve a problem which previous evidence suggests may reduce their decision-making ability. One intervention is designed to improve self-esteem, another is designed to reduce negative beliefs about psychosis (‘self-stigma’) and another is designed to help people with psychosis gather more information before making decisions. **We will record how many people participate in and complete our trial (including data completion rates), and we will ask 12 people (6 patient participants and 6 referrers or clinicians) for their views on what they liked and did not like about the trial, and we will ask a further 10 patient participants to discuss in more detail the nature of any change in decision-making.** All this information will help us ensure a larger DEC:IDES trial is more acceptable to people with psychosis.   - 1. **Types of data**   We will gather both quantitative and qualitative data.  A trained and supervised research assistant (RA) will gather the following information via interviews with each patient participant (hereafter P’):   - P’s name, age and address (for follow up) - P’s current and past psychiatric treatment - P’s mental health condition and related experiences - P’s views on treatments such as medication and hospital care - P’s self-esteem, current mood and anxiety - P’s views about their diagnosis - What services P has used - P’s decision-making   Any missing information on service usage will be gathered with P’s consent, via access to their medical records.  The RA will also gather the following information from the P’s keyworker or care coordinator:   - Information relevant to assessing risk of harm to the participant, researcher or others. This will include previous violence and previous self-harm or suicide, and drug and alcohol use.   The RA will also interview referrers and clinicians involved in the care of P, to examine their experiences of the trial. The RA will gather the following:   - Their name, age, occupation and place of work - Their relationship to P - Their professional qualifications and training   **1.3 Format and scale of the data**  Data will be collected on paper and encrypted audio from up to 60 patient participants on up to 3 occasions over a 20-month period (up to 16 of whom will be asked to provide both quantitative and qualitative data) and 6 clinician participants (qualitative data only). Only the informed consent forms and audio recordings will contain any participant identifiable data. Completion of the consent form will link the participant to a unique identification code which will be attached to all subsequent data (questionnaires and recordings). Data is not anonymous as such, but will not be traceable to any particular individual unless access to the consent form is obtained.  The list of codes and consent forms will be kept separately from one another in different secure, encrypted and password-protected files. Only the lead researcher and researchers with direct participant contact (including supervisors) will have access to this file. No participant identifiable data will be removed from NHS premises. All data will be held securely and treated in accordance with  NHS policies on Confidentiality and Data Protection as well as the BPS (2009) Code of Ethics and Conduct and BPS (2014) Code of Human Research Ethics guidelines documents and the study will adhere to the principles of Good Clinical Practice.  Non-personally identifiable data will be entered into a database for analysis. This format will allow for data analysis and long term retention. | |
| **2. Data collection / generation** | |
| **2.1 Methodologies for data collection / generation**  Patient participant data from standardised questionnaires and interviews will be collected in up to 3 meetings with the researcher. Up to 16 patient participants will be invited to an additional meeting, where qualitative data will be gathered. We wil also interview up to 6 clinician participants. All interviews will be audio-recorded on encrypted digital recorders. Participant identifiable information (name, date of birth, address) will be recorded on consent forms and will be held separately (in hard copy format) from their research data and will not leave NHS premises.  Paper and audio recorder with be stored in locking filing cabinets in NHS premises. Paper will be scanned, stripped of PII and stored on encrypted and password protected NHS databases. Audio will either be used to score responses and then destroyed, or will be transcribed before being destroyed. Transcriptions will be stored as with paper. Scores (derived from inspection of paper and audio) will be uploaded to ENU databases, with unique codes linking back to consent form etc.  Participant identifiable information will be linked to their research data by a code accessible only to researchers and approved personnel from ENU or the host NHS organisation, to enable data linkage and removal if requested by a participant and/or audit. Qualitative data will be transcribed on NHS premises by the RA, and all patient identifiable information will be removed from the transcripts. All audio recordings will be stored securely on encrypted and non web-linked electronic storage, and will not leave NHS premises. Transcriptions and other data without PII will be transferred to ENU data storage according to local NHS protocols, but using password protection and encryption at a minimum  **2.2 Data quality and standards**  A trained and supervised Research Assistant (RA) will administer measures to all participants. All measures will be valid and reliable, and RAs will receive regular training to ensure consistency and reliability in administration and scoring.  For the qualitative component, an interview schedule will be developed by Professor Brian Williams and Dr Paul Hutton to explore participant experience, any problems identified in the quantitative data, and to understand the nature of any change in participant decision-making following the interventions.  CI will inspect all transripts to ensure any identifying information is changed or removed (age, name, illness duration etc). We can only aim for pseudoanonymity with qual research (hence our avoidance of term ‘anonymity’) given we are using quotes and case materal etc. | |
| **3. Data management, documentation and curation** | |
| **3.1 Managing, storing and curating data.**  Research data, free of participant identifiable information, will be stored on the University’s X-drive. **University-managed data storage** is resilient, with multiple copies stored in more than one physical location and protection against corruption. Daily backups are kept for 14 days and monthly backups for an additional year.  **3.2 Metadata standards and data documentation**  All research data will be organised as per the Universities metadata standards <http://staff.napier.ac.uk/services/research-innovation-office/research-data/Pages/Organising.aspx>  **3.3 Data preservation strategy and standards**  The [Edinburgh Napier Data Management Policy](http://staff.napier.ac.uk/services/research-innovation-office/Documents/Research%20Data%20Management%20Policy.pdf) states requires research data to be retained after project completion if they substantiate research findings, are of potential long-term value or support a patent for at least 10 years. The policy also requires that funders and/or sponsors requirements are met. The Chief Scientist Office is funding this project, and requires data to be stored for a minimum of 5 years after completion. Long term storage is provided through the University data repository. | |
| **4. Data security and confidentiality of potentially disclosive information** | |
| **4.1 Formal information/data security standards**  N/A  **4.2 Main risks to data security**  Upon entering the study, participants will receive a unique identification number. There will be a code linking these numbers with participants’ personal information. No personal identification information will be removed from hospital premises. The code linking these details will be stored in a secure encrypted and password protected file on NHS computer systems, only accessible to the research team and approved staff from ENU and the host NHS organisation (for audit and data compliance purposes). All other data will be free of participant identifiable information. Hard copy questionnaires will initially be stored in locked filing cabinets, separately to the identification key, before being scanned and uploaded to a secure University system where it will be stored in a password protected file. All personal identifiable information (consent forms, contact details, audio recordings) will be destroyed 6-12 months after study completion. All research data, free of participant identifiable information, will be stored on the University’s X-drive. **University-managed data storage** is resilient, with multiple copies stored in more than one physical location and protection against corruption. Daily backups are kept for 14 days and monthly backups for an additional year. | |
| **5. Data sharing and access** | |
| Identify any data repository (-ies) that are, or will be, entrusted with storing, curating and/or sharing data from your study, where they exist for particular disciplinary domains or data types. [Information on repositories is available here.](http://www.wellcome.ac.uk/About-us/Policy/Spotlight-issues/Data-sharing/Guidance-for-researchers/WTX060360.htm)  **5.1 Suitability for sharing**  Data collected in this study will be stored for 10 years and will not be made available to other researchers (because of the small sample sizes involved). Summary data will be provided in publications, but the individual data wont be open given the risks this raises with identification of participants.   - 1. **Discovery by potential users of the research data**   Other researchers will be aware of the data set as peer reviewed articles will be set for publication. Datasets will be allocated a DOI and stored on our Research Repository in accordance with the University research data deposit process.  **5.3 Governance of access**  The data will not be shared except for the purposes of audit by ENU or NHS approved staff.  **5.4 The study team’s exclusive use of the data**  With the exception of auditing by appropriate authorities, exclusive access to data by the study team will be for 10 years.  **5.5 Restrictions or delays to sharing, with planned actions to limit such restrictions**  Participants will give their consent for their data to be stored for the purposes of audit and substantiation of the research findings.  **5.6 Regulation of responsibilities of users**  Data will not be shared with third parties, except for the purposes of audit and data management compliance. | |
| **6. Responsibilities** | |
| The first point of contact for all queries in relation to this data is the PrincipaI Investigator (PI), Dr Paul Hutton. The PI will also have overall responsibility for the production and maintenance of metadata. Preparation and upload of the research data will be carried out by the team with the support of the University’s Information Services staff. | |
| **7. Relevant institutional, departmental or study policies on data sharing and data security** | |
| *Please complete, where such policies are (i) relevant to your study, and (ii) are in the public domain, e.g. accessible through the internet.*  *Add any others that are relevant* | |
| **Policy** | **URL or Reference** |
| Data Management Policy & Procedures | <https://staff.napier.ac.uk/services/research-innovation-office/Documents/Research%20Data%20Management%20Policy.pdf> |
| Data Security Policy | <https://staff.napier.ac.uk/services/cit/infosecurity/Pages/InformationSecurityPolicy.aspx> |
| Data Sharing Policy | <https://staff.napier.ac.uk/services/governance-compliance/governance/DataProtection/Pages/DataSharing.aspx> |
| Institutional Information Policy |  |
| Other: |  |
| Other |  |
| **8. Author of this Data Management Plan (Name)** and, if different to that of the Principal Investigator, their **telephone & email contact details** | |
| Dr Paul Hutton (Associate Professor & Chief Investigator)  [p.hutton@napier.ac.uk](mailto:p.hutton@napier.ac.uk) | |

5. Trial Steering Committee terms of reference (version 2)


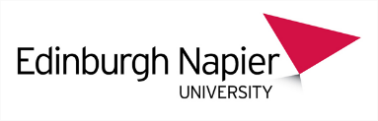


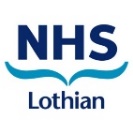


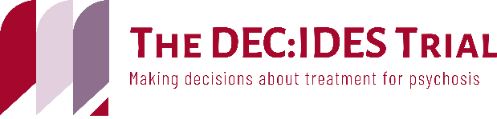


**The DEC:IDES Trial: Trial Steering Committee (TSC)**

1. **TERMS OF REFERENCE**
   1. To monitor and supervise the progress of the DEC:IDES trial towards its interim and overall objectives.
   2. To review at regular intervals relevant information from other sources (e.g. other related trials).
   3. The TSC will incorporate some functions normally allocated to a Data Monitoring and Ethics Committee (DMEC), such as reviewing interim data and monitoring serious adverse event (SAE) reports.
   4. To respond to requests for information about the progress of the trial from the host organisations (NHS Lothian, Pennine and Lancashire).
2. **MEMBERSHIP**

**Independent members:**

- 1. Dr Craig Whittington, Group Director Literature Synthesis & Biostatistics, Real World Evidence Generation at Sanofi **[Chair]**
  2. Prof Daniel Freeman, Lead for Oxford Cognitive Approached to Psychosis (Department of Psychiatry, Oxford University), Consultant Clinical Psychologist (Oxford Health NHS Foundation Trust)
  3. Olympia Gianfrancesco, Service user representative
  4. Prof Colin Mackay, Professor of Mental Health and Capacity Law (School of Heath & Social Care, Edinburgh Napier University)
  5. Frances Simpson, Chief Executive, Support in Mind Scotland.
  6. Tom Todd, Service user Representative
  7. Dr Filippo Varese, Clinical Senior Lecturer in Psychology (Division of Psychology & Mental Health, University of Manchester), Clinical Psychologist (HCPC)

**Research team:**

- 1. Dr Paul Hutton, Associate Professor of Therapeutic Interventions
     & Lead for Postgraduate Research (School of Health & Social Care, Edinburgh Napier University)) **[Chief investigator]**
  2. Dr Amanda Woodrow, Research Assistant (School of Health & Social Care, Edinburgh Napier University); Honorary Assistant Psychologist (NHS Lothian)

1. **GUIDANCE NOTES**
   1. **Meetings**

Meetings will take place at least every 4 months over the course of the DEC:IDES trial, although there may be periods when more frequent meetings are necessary. Meetings will be called for and organised by the Chief Investigator (CI). Papers for meetings will be circulated well in advance of the meeting rather than tabled and an accurate minute of the meeting will be prepared by the Research Assistant and agreed by all members.

Should the need arise, independent members of the TSC have the option to meet without members of the research team being present.

- 1. **Trial Steering and Management**

The role of the TSC is to provide overall support to the trial on behalf of the host NHS organisations (Lothian, Pennine and Lancashire). In particular, the TSC should concentrate on the trial’s adherence to protocol, patient safety and the consideration of new information. Day-to-day management of the trial is the responsibility of the CI.

- 1. **Patient Safety**

In all the deliberations of the TSC the rights, safety and well-being of the trial participants are the most important considerations and should prevail over the interests of science and society. The TSC should ensure that the protocol demands freely given consent from every trial participant. As the study is also open to individuals who may lack the capacity to consent to research, the relevant legislation must be adhered to. In Scotland, Guardians, Welfare Attorneys or nearest relatives will be asked to provide consent on participants’ behalf to taking part. In England, Consultees will be asked to provide advice (which is binding on the researcher) as to whether a participant would wish to take part. The TSC should look closely at the patient information provided and advise the investigators on its completeness and suitability.

- 1. **Progress of the Trial**

It is the role of the TSC to monitor the progress of the trial and to maximise the chances of completing the study within the time scale agreed by the CSO. At the first TSC meeting, targets for recruitment, data collection, compliance etc. should be agreed with the investigators. These targets should not be “set in stone” but are designed to act as a gauge of trial progress. The TSC should agree a set of data, based on the study targets, which will be presented to each TSC for review.

- 1. **Adherence to Protocol**

The protocol will be presented as an agenda item at the first TSC meeting. If the investigators are required to make any changes to the protocol during the course of trial, necessary approvals will be sought from the relevant approving bodies.

- 1. **Consideration of New Information**

The TSC should consider new information relevant to the trial including the results of others studies. It is the responsibility of the CI and the Chair and other independent members of the TSC to bring to the attention of the TSC any results from other studies that may have a direct bearing on the future conduct of the trial.

On consideration of this information the TSC should recommend appropriate action, such as changes to the trial protocol, additional patient information or stopping of the study. The rights, safety and well-being of the trial participants should be the most important considerations in these deliberations.

It is the responsibility of the investigators to notify the TSC and relevant regulatory authorities of any unexpected serious adverse events during the course of the study.

- 1. **Data Monitoring and Ethics Committee**

As a pilot feasibility trial, DEC:IDES does not require a separate Data Monitoring and Ethics Committee (DMEC). Therefore, some of the functions normally carried out by a DMEC will be incorporated into the TSC. This includes reviewing interim data and reports of serious adverse events.

- 1. **MRC GCP**

The TSC should endeavour to ensure that the trial is conducted at all times to the rigorous standards set out in the MRC Guidelines for Good Clinical Practice.

6. Combined reporting checklist

| **Section** | **SPIRIT** | **Pp.** | **CONSORT-PILOT** | **Pp.** | **CONSORT-HARMS** | **Pp.** | **CONSERVE-SPIRIT** | **Pp.** | **CONSORT-SPI** | **Pp.** |
| --- | --- | --- | --- | --- | --- | --- | --- | --- | --- | --- |
| **Title & abstract** | | | | | | | | | | |
| Title | 1. Descriptive title identifying the study design, population, interventions, and, if applicable, trial acronym | 1 | 1a. Identification as a pilot or feasibility randomised trial in the title | 1 | 1. If the study collected data on harms and benefits, the title or abstract should so state. | 1 |  |  |  |  |
| Abstract |  |  | 1b. Structured summary of pilot trial design, methods, results, and conclusions (see CONSORT abstract extension for pilot trials) | 2 | 1. If the study collected data on harms and benefits, the title or abstract should so state. | 2 |  |  | 1b. Refer to CONSORT extension for social and psychological intervention trial abstracts | 2 |
| **Administrative information** | | | | | | | | | | |
| Trial registration | 2a. Trial identifier and registry name. If not yet registered, name of intended registry | 2, 5 | 23. Registration number for pilot trial and name of trial registry | 2, 5 |  |  |  |  |  |  |
|  | 2b. All items from the World Health Organization Trial Registration Data Set | A |  |  |  |  |  |  |  |  |
| Protocol version | 3. Date and version identifier | A |  |  |  |  |  |  |  |  |
| Funding | 4. Sources and types of financial, material, and other support | 13 |  |  |  |  |  |  |  |  |
| Roles and responsibilities | 5a. Names, affiliations, and roles of protocol contributors | 1, 13 |  |  |  |  |  |  |  |  |
|  | 5b. Name and contact information for the trial sponsor | A |  |  |  |  |  |  |  |  |
|  | 5c. Role of study sponsor and funders, if any, in study design; collection, management, analysis, and interpretation of data; writing of the report; and the decision to submit the report for publication, including whether they will have ultimate authority over any of these activities | 13 |  |  |  |  |  |  |  |  |
|  | 5d. Composition, roles, and responsibilities of the coordinating centre, steering committee, endpoint adjudication committee, data management team, and other individuals or groups overseeing the trial, if applicable (see Item 21a for data monitoring committee) | 5, 13 |  |  |  |  |  |  |  |  |
| Declaration of interests |  | 14 |  |  |  |  |  |  | 25. Declaration of any other interests | 14 |
| Stakeholder involvement |  |  |  |  |  |  |  |  | 26a. Any involvement of the intervention developer in the design, conduct, analysis or reporting of the trial | 9 |
|  |  |  |  |  |  |  |  |  | 26b. Other stakeholder involvement in trial design, conduct, or analyses. | 14 |
|  |  |  |  |  |  |  |  |  | 26c. Incentives offered as part of the trial | 6 |
| **Introduction** | | | | | | | | | | |
| Background and rationale | 6a. Description of research question and justification for undertaking the trial, including summary of relevant studies (published and unpublished) examining benefits and harms for each intervention | 3-4 | 2a. Scientific background and explanation of rationale for future definitive trial, and reasons for randomised pilot trial | 3-4 | 2. If the study addresses both harms and benefits, the introduction should so state. | 4 |  |  | 2b. If pre-specified, how the intervention was hypothesised to work. | 8-9 |
|  | 6b. Explanation for choice of comparators | 9, 12 |  |  |  |  |  |  |  |  |
| Objectives | 7. Specific objectives or hypotheses | 4 | 2b. Specific objectives or research questions for pilot trial | 4 |  |  |  |  |  |  |
| Trial design | 8. Description of trial design including type of trial (eg, parallel group, crossover, factorial, single group), allocation ratio, and framework (eg, superiority, equivalence, noninferiority, exploratory) | 4-7 | 3a. Description of pilot trial design (such as parallel, factorial) including allocation ratio | 4-7 |  |  |  |  |  |  |
|  |  |  | 3b. Important changes to methods after pilot trial commencement (such as eligibility criteria), with reasons | 11, T.4 |  |  |  |  |  |  |
| **Methods: Participants, interventions, and outcomes** | | | | | | | | | | |
| Study setting | 9. Description of study settings (eg, community clinic, academic hospital) and list of countries where data will be collected. Reference to where list of study sites can be obtained | 5-6 |  |  |  |  |  |  |  |  |
| Eligibility criteria | 10. Inclusion and exclusion criteria for participants. If applicable, eligibility criteria for study centres and individuals who will perform the interventions (eg, surgeons, psychotherapists) | 5-6 | 4c. How participants were identified and consented | 6 |  |  |  |  | 4a. Where applicable, eligibility criteria for settings and those delivering the interventions. | 9 |
| Interventions | 11a. Interventions for each group with sufficient detail to allow replication, including how and when they will be administered | 8-9, T.3 |  |  |  |  |  |  | 5b. Where other informational materials about delivering the intervention can be accessed. | 9 |
|  | 11b. Criteria for discontinuing or modifying allocated interventions for a given trial participant (eg, drug dose change in response to harms, participant request, or improving/worsening disease) | 9 |  |  |  |  |  |  | 5c. Where applicable, how intervention providers were assigned to each group. | 7 |
|  | 11c. Strategies to improve adherence to intervention protocols, and any procedures for monitoring adherence (eg, drug tablet return, laboratory tests) | 9 |  |  |  |  |  |  |  |  |
|  | 11d. Relevant concomitant care and interventions that are permitted or prohibited during the trial | 4 |  |  |  |  |  |  |  |  |
| Outcomes | 12. Primary, secondary, and other outcomes, including the specific measurement variable (eg, systolic blood pressure), analysis metric (eg, change from baseline, final value, time to event), method of aggregation (eg, median, proportion), and time point for each outcome. Explanation of the clinical relevance of chosen efficacy and harm outcomes is strongly recommended | 4, 7-8, A | 6a. Completely defined prespecified assessments or measurements to address each pilot trial objective specified in 2b, including how and when they were assessed | 4, 7-8, A | 6. List addressed adverse events with definitions for each (with attention, when relevant, to grading, expected vs. unexpected events, reference to standardized and validated definitions, and description of new definitions).  Clarify how harms-related information [will be] collected (mode of data collection, timing, attribution methods, intensity of ascertainment, and harms-related monitoring and stopping rules, if pertinent). | 8 |  |  |  |  |
|  |  |  | 6b. Any changes to pilot trial assessments or measurements after the pilot trial commenced, with reasons | 11 |  |  |  |  |  |  |
|  |  |  | 6c. If applicable, prespecified criteria used to judge whether, or how, to proceed with future definitive trial | 7 |  |  |  |  |  |  |
| Participant timeline | 13. Time schedule of enrolment, interventions (including any run-ins and washouts), assessments, and visits for participants. A schematic diagram is highly recommended | 4-5, T1, T2 |  |  |  |  |  |  |  |  |
| Sample size | 14. Estimated number of participants needed to achieve study objectives and how it was determined, including clinical and statistical assumptions supporting any sample size calculations | 6 | 7a. Rationale for numbers in the pilot trial | 6 |  |  |  |  |  |  |
| Recruitment | 15. Strategies for achieving adequate participant enrolment to reach target sample size | 6 |  |  |  |  |  |  |  |  |
| **Methods: Assignment of interventions (for controlled trials)** | | | | | | | | | | |
| Allocation: |  |  |  |  |  |  |  |  |  |  |
| Sequence generation | 16a. Method of generating the allocation sequence (eg, computer-generated random numbers), and list of any factors for stratification. To reduce predictability of a random sequence, details of any planned restriction (eg, blocking) should be provided in a separate document that is unavailable to those who enrol participants or assign interventions | 6-7 | 8b. Type of randomisation(s); details of any restriction (such as blocking and block size) | 6-7 |  |  |  |  |  |  |
| Allocation concealment mechanism | 16b. Mechanism of implementing the allocation sequence (eg, central telephone; sequentially numbered, opaque, sealed envelopes), describing any steps to conceal the sequence until interventions are assigned | 6-7 |  |  |  |  |  |  |  |  |
| Implementation | 16c. Who will generate the allocation sequence, who will enrol participants, and who will assign participants to interventions | 6-7 |  |  |  |  |  |  |  |  |
| Blinding (masking) | 17a. Who will be blinded after assignment to interventions (eg, trial participants, care providers, outcome assessors, data analysts), and how | 6-7 |  |  |  |  |  |  |  |  |
|  | 17b. If blinded, circumstances under which unblinding is permissible, and procedure for revealing a participant’s allocated intervention during the trial | 7 |  |  |  |  |  |  |  |  |
| **Methods: Data collection, management, and analysis** | | | | | | | | | | |
| Data collection methods | 18a. Plans for assessment and collection of outcome, baseline, and other trial data, including any related processes to promote data quality (eg, duplicate measurements, training of assessors) and a description of study instruments (eg, questionnaires, laboratory tests) along with their reliability and validity, if known. Reference to where data collection forms can be found, if not in the protocol | 7-9 |  |  |  |  |  |  |  |  |
|  | 18b. Plans to promote participant retention and complete follow-up, including list of any outcome data to be collected for participants who discontinue or deviate from intervention protocols | 6, 7, 8 |  |  |  |  |  |  |  |  |
| Data management | 19. Plans for data entry, coding, security, and storage, including any related processes to promote data quality (eg, double data entry; range checks for data values). Reference to where details of data management procedures can be found, if not in the protocol | A |  |  |  |  |  |  |  |  |
| Statistical methods | 20a. Statistical methods for analysing primary and secondary outcomes. Reference to where other details of the statistical analysis plan can be found, if not in the protocol | 10-11, A | 12a. Methods [which will] used to address each pilot trial objective whether qualitative or quantitative | 10-11, A | 12. Describe plans for presenting and analyzing information on harms (including coding, handling of recurrent events, specification of timing issues, handling of continuous measures, and any statistical analyses). | 10-11, A |  |  | 12a. How missing data were handled, with details of any imputation method | A |
|  | 20b. Methods for any additional analyses (eg, subgroup and adjusted analyses) | A |  |  |  |  |  |  |  |  |
|  | 20c. Definition of analysis population relating to protocol non-adherence (eg, as randomised analysis), and any statistical methods to handle missing data (eg, multiple imputation) | 10 |  |  |  |  |  |  |  |  |
| **Methods: Monitoring** | | | | | | | | | | |
| Data monitoring | 21a. Composition of data monitoring committee (DMC); summary of its role and reporting structure; statement of whether it is independent from the sponsor and competing interests; and reference to where further details about its charter can be found, if not in the protocol. Alternatively, an explanation of why a DMC is not needed | 5 |  |  |  |  |  |  |  |  |
|  | 21b. Description of any interim analyses and stopping guidelines, including who will have access to these interim results and make the final decision to terminate the trial | 5, A |  |  |  |  |  |  |  |  |
| Harms | 22. Plans for collecting, assessing, reporting, and managing solicited and spontaneously reported adverse events and other unintended effects of trial interventions or trial conduct | 8-9, 10-11 |  |  |  |  |  |  |  |  |
| Auditing | 23. Frequency and procedures for auditing trial conduct, if any, and whether the process will be independent from investigators and the sponsor | 5 |  |  |  |  |  |  |  |  |
| **Ethics and dissemination** | | | | | | | | | | |
| Research ethics approval | 24. Plans for seeking research ethics committee/institutional review board (REC/IRB) approval | 5, 14 | 26. Ethical approval or approval by research review committee, confirmed with reference number | 5, 14 |  |  |  |  |  |  |
| Protocol amendments | 25. Plans for communicating important protocol modifications (eg, changes to eligibility criteria, outcomes, analyses) to relevant parties (eg, investigators, REC/IRBs, trial participants, trial registries, journals, regulators) | 5, 11 |  |  |  |  | Complete CONSERVE SPIRIT checklist, covering each section of manuscript. If important modifications occurred check “direct impact” and/or “mitigating strategy” in the checklist and describe the changes in the trial manuscript or supplement. Check “no change” for items that are unaffected in the extenuating circumstance. | A |  |  |
| Extenuating circumstances |  |  |  |  |  |  | I. Describe the circumstances and how they constitute extenuating circumstances | 11, 13, T4 |  |  |
| Important modifications |  |  |  |  |  |  | IIa.Describe how the modifications are important modifications. | 11, 13, T4 |  |  |
|  |  |  |  |  |  |  | IIb. Describe the impacts and mitigating strategies, including their rationale and implications for the trial | 11, 13, T4 |  |  |
|  |  |  |  |  |  |  | IIc. Provide a modification timeline | 11, 13, T4 |  |  |
| Responsible parties |  |  |  |  |  |  | III. State who planned, reviewed and approved the modifications. | 11, 13, T4 |  |  |
| Interim data |  |  |  |  |  |  | IV. If modifications were informed by trial data, describe how the interim data were used, including whether they were examined by study group, and whether the individuals reviewing the data were blinded to the treatment allocation. | 11, 13, T4 |  |  |
| Consent or assent | 26a. Who will obtain informed consent or assent from potential trial participants or authorised surrogates, and how (see Item 32) | 6, 14 |  |  |  |  |  |  |  |  |
|  | 26b. Additional consent provisions for collection and use of participant data and biological specimens in ancillary studies, if applicable | NA |  |  |  |  |  |  |  |  |
| Confidentiality | 27. How personal information about potential and enrolled participants will be collected, shared, and maintained in order to protect confidentiality before, during, and after the trial | A |  |  |  |  |  |  |  |  |
| Declaration of interests | 28. Financial and other competing interests for principal investigators for the overall trial and each study site | 15 |  |  |  |  |  |  |  |  |
| Access to data | 29. Statement of who will have access to the final trial dataset, and disclosure of contractual agreements that limit such access for investigators | 14, A |  |  |  |  |  |  |  |  |
| Ancillary and post-trial care | 30. Provisions, if any, for ancillary and post-trial care, and for compensation to those who suffer harm from trial participation | NA |  |  |  |  |  |  |  |  |
| Dissemination policy | 31a. Plans for investigators and sponsor to communicate trial results to participants, healthcare professionals, the public, and other relevant groups (eg, via publication, reporting in results databases, or other data sharing arrangements), including any publication restrictions | 13 |  |  |  |  |  |  |  |  |
|  | 31b. Authorship eligibility guidelines and any intended use of professional writers | NA |  |  |  |  |  |  |  |  |
|  | 31c. Plans, if any, for granting public access to the full protocol, participant-level dataset, and statistical code | 14, A |  |  |  |  |  |  |  |  |
| **Discussion** | | | | | | | | | | |
| Limitations |  |  | 20. Pilot trial limitations, addressing sources of potential bias and remaining uncertainty about feasibility | 11-12 |  |  |  |  |  |  |
| Generalisability |  |  | 21. Generalisability (applicability) of pilot trial methods and findings to future definitive trial and other studies | 11-12 |  |  |  |  |  |  |
| **Appendices** | | | | | | | | | | |
| Informed consent materials | 32. Model consent form and other related documentation given to participants and authorised surrogates | A |  |  |  |  |  |  |  |  |
| Biological specimens | 33. Plans for collection, laboratory evaluation, and storage of biological specimens for genetic or molecular analysis in the current trial and for future use in ancillary studies, if applicable | NA |  |  |  |  |  |  |  |  |
|  |  |  |  |  |  |  |  |  |  |  |

Note: A = appendix

7. CONSERVE Checklist

| CONSERVE-SPIRIT Extension: [DATE] | | | | | |
| --- | --- | --- | --- | --- | --- |
| Item | Item Title | Description | | | Page No. |
| I. | Extenuating Circumstances | Describe the circumstances and how they constitute extenuating circumstances. | | | See above |
| II. | Important Modifications | 1. Describe how the modifications are important modifications. | | | See above |
|  |  | 1. Describe the impacts and mitigating strategies, including their rationale and implications for the trial. | | | (see below) |
|  |  | 1. Provide a modification timeline. | | | See above |
| III. | Responsible Parties | State who planned, reviewed and approved the modifications. | | | See above |
| IV. | Interim data | If modifications were informed by trial data, describe how the interim data were used, including whether they were examined by study group, and whether the individuals reviewing the data were blinded to the treatment allocation. | | | See above |
| SPIRIT Item and Number | | For each row, if important modifications occurred, check one or both of “impact” and/or “mitigating strategy” and describe the changes in the protocol. Check “no change” for items that are unaffected in the extenuating circumstance. | | | Page No. |
|  |  | No Change | Impact* | Mitigating Strategy** |  |
| 1 | Title | X |  |  |  |
| 2 | Trial registration | X |  |  |  |
| 3 | Protocol version |  | X | X | Table 4 |
| 4 | Funding |  | X | X | 13 |
| 5 | Roles and responsibilities |  | X | X | 13, Table 4 |
| 6 | Background and rationale | X |  |  |  |
| 7 | Objectives | X |  |  |  |
| 8 | Trial design | X |  |  |  |
| 9 | Study setting |  | X | X | 13, Table 4 |
| 10 | Eligibility criteria | X |  |  |  |
| 11 | Interventions | X |  |  |  |
| 12 | Outcomes | X |  |  |  |
| 13 | Participant timeline | X |  |  |  |
| 14 | Sample size |  | X | X | 11, Table 4 |
| 15 | Recruitment |  | X | X | 11, Table 4 |
| 16 | Allocation | X |  |  |  |
| 17 | Blinding (masking) | X |  |  |  |
| 18 | Data collection methods |  | X | X | 11, Table 4 |
| 19 | Data management | X |  |  |  |
| 20 | Statistical methods | X |  |  |  |
| 21 | Data monitoring | X |  |  |  |
| 22 | Harms | X |  |  |  |
| 23 | Auditing | X |  |  |  |
| 24 | Research ethics approval | X |  |  |  |
| 25 | Protocol amendments |  | X | X | 11, Table 4 |
| 26 | Consent or assent |  | X | X | Table 4 |
| 27 | Confidentiality | X |  |  |  |
| 28 | Declaration of interests | X |  |  |  |
| 29 | Access to data | X |  |  |  |
| 30 | Ancillary and post-trial care | X |  |  |  |
| 31 | Dissemination policy | X |  |  |  |
| 32 | Informed consent materials | X |  |  |  |
| 33 | Biological specimens | NA |  |  |  |
| *Aspects of the trial that are directly affected or changed by the extenuating circumstance and are not under the control of investigators, sponsor or funder.  **Aspects of the trial that are modified by the study investigators, sponsor or funder to respond to the extenuating circumstance or manage the direct impacts on the trial. | | | | | |

8. Recruitment poster – participants (version 2)


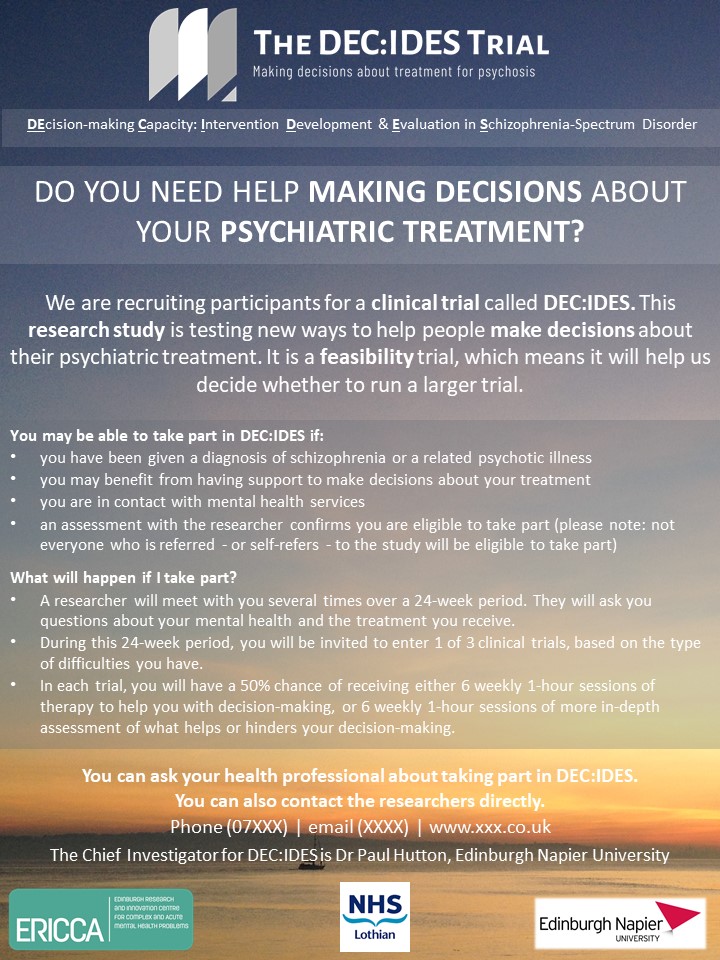


Insert NHS logo

9. Participant information sheet (version 4)


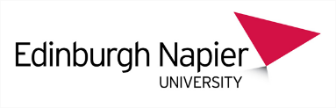


Logo of host NHS organisation to be inserted here


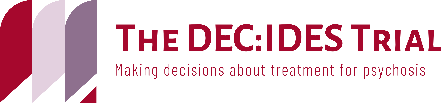


**Participant Information Sheet**

**DE**cision-making **C**apacity: **I**ntervention **D**evelopment & **E**valuation in **S**chizophrenia-spectrum disorder: The DEC:IDES Trial

**You are being invited to take part in a research study. Before you decide whether or not to take part, it is important for you to understand why the research is being done and what it will involve. Please take time to read the following information carefully. Talk to others about the study if you wish. Contact us if there is anything that is not clear or if you would like more information. Take time to decide whether or not you wish to take part.**

**Do I have to take part?**

No, it is up to you to decide whether or not to take part. If you do decide to take part you will be given this information sheet to keep and be asked to sign a consent form. If you decide to take part you are still free to withdraw at any time and without giving a reason. Deciding not to take part or withdrawing from the study will not affect the healthcare that you receive, or your legal rights.

**What is the purpose of the study?**

Some people hear or see things that others do not, or believe things that others do not. They may be worried that others want to harm them. Sometimes, these experiences and beliefs can lead to a person being diagnosed with a mental health problem such as schizophrenia or psychosis. Sometimes, psychosis can also affect a person’s ability to make their own decisions about treatment – such as taking medication or going into hospital. This means other people, including doctors, may make these decisions instead.

Over the last few years we have been developing new approaches to help people with psychosis make their own decisions about treatment. However, to find out if these approaches are helpful, we need to carry out ‘clinical trials’. Trials are a kind of research study that can compare how helpful different treatment approaches are. However, to produce reliable findings, trials often need to include a lot of people and they need to be very carefully designed.

To ensure these larger trials are well-designed, it is common to run several small trials first. These are known as ‘feasibility’ or ‘pilot’ trials. Although these small trials cannot tell us whether a new approach is effective, they do provide essential information for designing the larger trials.

The aim of our study is therefore to complete several small trials of new approaches to help people with psychosis make their own decisions about treatment. This will help us design larger trials of these new approaches, which will help to ensure they produce reliable results.

**Why have I been asked to take part?**

You have been invited to take part because:

- your doctor has given you a diagnosis of schizophrenia or a related psychotic illness.
- you may benefit from having support to make decisions about your treatment
- you are in contact with mental health services in (name of NHS organisation).

Please note further assessment is required to confirm whether you are eligible to take part in the study. The researchers will only know this once they have met with you and asked you some additional questions about your mental health and treatment. We will let you know the outcome of this assessment as soon as possible.

**What will happen if I take part?**

- A staff member from your NHS mental health team will give you this form to read. If you are interested in taking part, then this staff member will ask you for your permission to share your name, contact details and some information about your condition and care with the researcher. This will allow the researcher to begin to assess whether you are able to take part, and to contact you directly.
- With your agreement, the researcher will arrange to meet with you, either on the phone or in person. This will allow you to ask the researcher any questions you like about the study. If you decide you don’t want to take part, then the researcher will not contact you again.
- If you remain interested in taking part, then the researcher will contact you no sooner than 2 days after this first meeting, to give you time to think about it before deciding. You can have a longer time if you prefer. The researcher will then invite you to meeting in person, where they will assess in more detail whether you are eligible to take part. They will ask you some questions about your mental health and the treatment you receive. If this assessment confirms you are eligible to take part then the researcher will ask you to sign a consent form. The researcher will check you understand everything on the form before you sign it.
- Sometimes, a person may lose the ability to decide whether or not to continue taking part in a study. This can happen if they become unwell. If this happens to you and you live in Scotland, we will ask your nearest relative, welfare attorney or guardian if they would like to give consent on your behalf to continue with the study. At all times we will follow the legal requirements of the Adults with Incapacity (Scotland) Act 2000. This means you would still be free to withdraw from the study at any time and without giving a reason.
- If you live in England and lose the ability to decide whether or not to continue taking part, we will seek and follow the opinion of your ‘Consultee’ as to whether you would wish to continue. Your Consultee will be someone who you know and trust. At all times we will follow the legal requirements of the Mental Capacity (England and Wales) Act 2005. This means you would still be free to withdraw from the study at any time and without giving a reason.
- If you decide to take part, then a researcher will meet with you several times over a 24-week period. In each meeting, they will ask you questions about your mental health and the treatment you receive. With your permission, these meetings will be audio-recorded.
- During this 24-week period, you will be invited to enter 1 of 3 clinical trials, based on the type of difficulties you have.
- In each trial, you will have a 50% chance of receiving either 6 weekly 1-hour sessions of therapy to help you with decision-making, or 6 weekly 1-hour sessions of more in-depth assessment of what helps or hinders your decision-making. This will be decided randomly. This means neither the researchers, the therapist or the participant can choose what they will receive. This is important for finding out which approach is most helpful and safe.
- The therapy sessions are designed to help you with one of the following type of difficulty:
  - Low self-esteem
  - Fears about your diagnosis
  - Gathering information before making decisions
- The assessment sessions are designed to gather more information about what helps or hinders your decision-making ability. If you are offered this, then the therapist will offer to meet with you after the study is over to discuss the results of this assessment. They will help you understand why you might have difficulties in decision-making, and what could help you with these. With your permission, we will share this information with your clinical team.
- We will also invite some participants to tell the researcher more about their experiences of taking part in the study. Some participants will be invited to tell us more about any improvements they had in their decision-making. These extra meetings should last around 1 hour and will also be audio-recorded. We may quote some of the things you tell us in any reports we produce, however we will not reveal your name or other information which could identify you.

**What are the possible benefits of taking part?**

- If you receive help for self-esteem, fears about your diagnosis or using more information before making decisions, then you may experience improvements in these areas.
- Taking part in this study may also help you understand the factors that help or hinder your ability to make decisions about your treatment. This may help your clinical team work out how best to support you in the future.
- The results of this study may also contribute to better mental health care and treatment for people experiencing similar difficulties.

**Who is doing this study?**

Dr Paul Hutton (Associate Professor, Edinburgh Napier University) is leading the overall study. The research team in Scotland includes Professors Thanos Karatzias, Brian Williams and Jill Stavert, and Associate Professor Nadine Dougall, from Edinburgh Napier University, Dr Suzanne O’Rourke from University of Edinburgh, Dr Sean Harper and Dr Andrew Watson from NHS Lothian.

The research team in England includes Dr Chris Taylor (Pennine Care NHS Foundation Trust), Dr James Kelly (Lancashire Care NHS Foundation Trust), Dr Peter Taylor (University of Manchester) and Professor Richard Emsley (King’s College London).

The study is funded by the Chief Scientist Office (Health Improvement, Protection and Services Research Committee – Response Mode Funding Scheme).

**What are the possible disadvantages and risks of taking part?**

- The number of assessments you might be asked to take part in ranges from 2 to 3. However some participants will also be invited to 1 or 2 additional meetings. These assessments and meetings can vary in length. This depends on lots of things, including how you are feeling at the time.
- We will offer breaks every 30 minutes and we will work flexibly according to your needs. Efforts will be made to make the meetings as comfortable as possible for you. We will reimburse you for your travel expenses and time, with a £10 Tesco voucher per each of the 2 or 3 assessments. At no point should you feel under pressure to complete the assessments.
- If any aspect of the study causes you distress or you become upset or anxious, this will be communicated to your mental health team team so that they can follow up with you.
- If it appears that you present a serious risk to yourself or to other people, this will also be communicated and standard NHS procedures would be followed.

**What if there is a problem?**

If you live in Scotland and have a concern about any aspect of this study please contact Dr Paul Hutton by phoning 07XXX or emailing p.hutton@napier.ac.uk.

If you live in England and are receiving care from Pennine Care NHS Foundation Trust, you can contact Dr Chris Taylor by phoning 07XXX or emailing [chrisdjtaylor@nhs.net](mailto:chrisdjtaylor@nhs.net).

If you live in England and are receiving care from Lancashire Care NHS Foundation Trust, you can contact Dr James Kelly by phoning 07XXX or emailing [j.a.kelly@lancaster.ac.uk](mailto:j.a.kelly@lancaster.ac.uk)

They will do their best to answer your questions.

If you would like to speak to someone independent from the study, please contact Dr David Carmichael (NHS Lothian) by phoning 07XXX or emailing [XXXX@XXXX](mailto:david.carmichael1@nhs.net)

In the unlikely event that something goes wrong and you are harmed during the research and this is due to someone‘s negligence then you may have grounds for a legal action for compensation against your NHS organisation but you may have to pay your legal costs. The normal National Health Service complaints mechanisms will still be available to you (if appropriate).

**What happens when the study is finished?**

For most participants, their involvement in the main part of the study will end around 24 weeks after they joined it. For some participants (those who joined the study later on), their involvement will last around 8 weeks only. Some participants will also be invited to attend additional meetings with the research team.

At the end of the research we will analyse the data from all the participants and write a report. Your data will be made anonymous as soon as possible and less than three months after your last session. The anonymous data will be kept for 10 years. We may quote some of the things you tell us in any reports we produce, however we will not reveal your name or other information which could identify you.

You can choose to have a summary of the results and outcome of the study sent to you once the research has been completed. This information will also be available on our study website ([www.XXX.co.uk](http://www.XXX.co.uk)).

**Will my taking part in the study be kept confidential?**

All the information we collect during the course of the research will be kept confidential and there are strict laws which safeguard your privacy at every stage. With your consent we will inform your GP that you are taking part.

To ensure that the study is being run correctly, we will ask your consent for responsible representatives from the Sponsor (Edinburgh Napier University) and [name of NHS organisation] to access your medical records and data collected during the study, where it is relevant to you taking part in this research. The Sponsor is responsible for overall management of the study and providing insurance and indemnity.

Should information come to light from disclosure during the study suggesting that you, another adult or a child is at risk of harm, standard NHS procedures would be followed to address this risk which may limit confidentiality. Any such disclosure would be handled within NHS policy and would protect confidentiality as best possible.

All identifiable information used at the beginning of the study will be destroyed as soon as possible and replaced with anonymous identifiers. All identifiable information will be kept in NHS sites, before being destroyed.

**What will happen to the results of the study?**

The study will be written up as a scientific journal article. The results will also be presented at conferences. You will not be identifiable in any published results. You can choose to have a summary of the results and outcome of the study sent to you once the research has been completed.

**Who is organising the research?**

This study is being organised and sponsored by Edinburgh Napier University.

**Who has reviewed the study?**

The study proposal has been reviewed by the Health Improvement, Protection and Services Research Committee of the Chief Scientist Office, Scotland. A favourable ethical opinion has been obtained from the Scotland A Research Ethics Committee. Edinburgh Napier University and the Research & Development departments of NHS Lothian, Pennine Care NHS Foundation Trust and Lancashire Care NHS Foundation Trust have also reviewed and approved the study.

**If you have any further questions about the study, the research team can be contacted using the details below:**

Dr Paul Hutton (Chief Investigator and Principal Investigator for Scotland) at [p.hutton@napier.ac.uk](mailto:p.hutton@napier.ac.uk) **or 07XXX**;

Dr Chris Taylor (Co-Investigator and Principal Investigator for Pennine Care NHS Foundation Trust) at [chrisdjtaylor@nhs.net](mailto:chrisdjtaylor@nhs.net) **or 07XXX**;

Dr James Kelly (Co-Investigator and Principal Investigator for Lancashire Care NHS Foundation Trust) at [j.a.kelly@lancaster.ac.uk](mailto:j.a.kelly@lancaster.ac.uk) **or 07XXX**;

If you wish to discuss this study with someone who is not involved in the research, please contact:

Dr David Carmichael (NHS Lothian) at [XXXX@XXXX](mailto:david.carmichael1@nhs.net) or **07XXX**

**If you wish to make a complaint about the study please contact NHS Lothian:**

**Patient Experience Team
NHS Lothian
2nd Floor
Waverley Gate
2-4 Waterloo Place
Edinburgh
EH1 3EG
Tel: 0131 536 3370
Email:** [**feedback@nhslothian.scot.nhs.uk**](mailto:feedback@nhslothian.scot.nhs.uk)

Thank you for taking the time to read this information sheet.

10. Referrer information sheet (version 2)


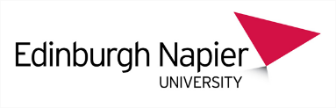


Logo of host NHS organisation to be inserted here


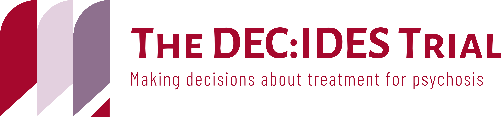


**Information for Referrers**

**DE**cision-making **C**apacity: **I**ntervention **D**evelopment & **E**valuation in **S**chizophrenia-spectrum disorder: The DEC:IDES Trial

**What is the research about?**

Treatment decision-making capacity (‘capacity’) refers to a person’s ability to make decisions about their treatment. It is an important issue for people diagnosed with a schizophrenia-spectrum disorder (‘psychosis’) because impaired capacity can mean a person does not understand what treatment options are available, or the implications of those options.

In 2018 the National Institute of Health & Care Excellence (NICE) called for clinical trials of interventions such as talking therapies to help people regain capacity. However, running these trials can take several years. One way of reducing this delay is to run several trials at the same time, as part of one bigger trial. This bigger type of trial is also called an ‘Umbrella’ trial. Although Umbrella trials have been used to accelerate the development of physical health interventions, they have yet to be used in mental health.

The main aims of this study are therefore to find out whether people with psychosis will take part in an Umbrella trial of talking therapies to improve their treatment decision-making capacity (the DEC:IDES trial), and to understand their experiences of participation.

**Why is the research being carried out?**

Before we can begin a larger version of the DEC:IDES trial, we need to find out whether people with psychosis will want to take part in it. In particular, we need to find out whether they will stay in the trial until it is finished, or whether they will leave early. We also need to understand why people might leave DEC:IDES early, so that we can improve it. For these reasons, we are running a smaller version first. This will involve 3 small clinical trials, each testing 1 of 3 different interventions. Each intervention has been designed to help participants resolve a problem which previous evidence suggests may reduce their decision-making ability. One intervention is designed to improve self-esteem, another is designed to reduce negative beliefs about psychosis (‘self-stigma’) and another is designed to help people with psychosis gather more information before making decisions.

We will record how many people participate in and complete our trial, and we will ask people for their views on what they liked and did not like about taking part. All this information will help us ensure the larger DEC:IDES trial is more acceptable to people with psychosis.

**Who is being asked to take part?**

In order to take part in the DEC:IDES pilot trial, potential participants need to be:

1. aged between 18 and 65 years;
2. able to be interviewed and complete the measures;
3. diagnosed with a schizophrenia-spectrum disorder (schizophrenia, schizoaffective disorder, delusional disorder, psychosis not otherwise specified, brief psychotic disorder);
4. presumed or already judged to have impaired treatment decision-making capacity.

They will unable to take part if they:

1. have a moderate to severe learning disability;
2. have psychosis of a predominantly organic origin (e.g. brain injury, physical health condition, epilepsy) or have a primary diagnosis of substance or alcohol use disorder;
3. cannot understand English sufficiently to engage in conversation without an interpreter.

Please note further assessment will be required to confirm whether your patient is eligible to take part in the study. The researchers will only know this once they have met with your patient and asked them some additional questions about their mental health and treatment. We will let them know the outcome of this assessment as soon as possible.

Capacity to consent to medication or a hospital admission is distinct from capacity to consent to research or psychological therapy. This means people who lack capacity to consent to medication or hospital care may still retain capacity to consent to research or psychological therapy. However some people with psychosis will lack capacity to make all these decisions. We do not wish to exclude these people from our trial. Instead, we will follow a specialised consent process, in accordance with legal guidelines in Scotland and England (see ‘How do I refer a patient for this research?’ for further details).

**What will happen to participants if they take part?**

Participants will first be invited to meet with a fully trained and supervised research assistant, who will complete an assessment. This will involve interviews and questionnaires, and may take 3 meetings to complete. The results of this assessment will tell us whether a participant mainly has difficulties with self-esteem, self-stigma or information-gathering.

If a participant mainly has difficulties with self-esteem, they will be able to take part in the self-esteem trial. Half of these participants will be offered the self-esteem intervention, whereas the other half will be offered assessment and support. This will be decided randomly. This means the researchers cannot choose who will receive the intervention.

If a participant mainly has difficulties with self-stigma, they will be able to take part in the self-stigma trial. Half of these participants will be offered the self-stigma intervention, whereas the other half will be offered assessment and support. Again, this will be decided randomly.

If a participant mainly has difficulties with information-gathering, they will be able to take part in information-gathering trial. Half of these participants will be offered the information-gathering intervention, whereas the other half will be offered assessment and support. As with the other trials, this will be decided randomly.

The self-esteem, self-stigma and information-gathering interventions each involve 6 weekly 1-hour therapy sessions, each of which will be provided by a fully trained and supervised psychological therapist. Each intervention will involve the following elements:

- Engagement and listening
- Positive regard and empathy
- Collaboration
- Development of a shared understanding of problem (a ‘psychological formulation’)
- Provision of written or audio-visual information relating to problem
- Between-session activity for participant
- Provision of structured self-help material relating to problem
- Testing of beliefs related to problems
- Practicing new strategies related to problem
- Development of a shared plan to maintain gains

‘Assessment and support’ will also involve 6 weekly 1-hour sessions with a psychological therapist. However, in these meetings, the therapist will work in collaboration with the person to complete a more detailed assessment of factors which help or hinder their decision-making capacity. They will provide engagement, listening, positive regard and empathy, but they will not develop a psychological formulation, nor will they provide the person with information relating to their problems. They will also not provide self-help material, or encourage the person to test their beliefs, practice new strategies or develop a shared plan for the future. Once the trial is over, however, the therapist will offer to meet with the person to share the results of the assessment and develop a psychological formulation. This may help them understand why they have difficulties in decision-making, and may help them identify ways of improving it. With the participant’s consent, this information will also be shared with the clinical team.

Eight weeks after a participant enters the trial, they will be invited to attend a post-treatment assessment with our research assistant. This will involve the same interviews and questionnaires which the participant completed in the first assessment, and may again take 3 meetings to complete. To ensure the assessments are free from bias, the research assistant will not know which intervention the participant has received. They will ask the participant not to tell them.

Twenty-four weeks after a participant enters the trial, they will be invited to attend a follow-up assessment with our research assistant. This will again involve the same interviews and questionnaires which the participant completed in the first assessment, and may again take 3 meetings to complete. As before, the research assistant will not know which intervention the participant has received.

Some participants will also be invited to meet the research assistant to discuss their experiences of taking part in the DEC:IDES trial, and what they liked and did not like.

We will also invite some of our participants’ clinicians to meet with the research assistant to discuss their experiences of the DEC:IDES trial, and what they liked and did not like.

Some participants may show an improvement in the extent to which they appreciate they have a mental health problem, for which they may need help. To fully understand the nature of this improvement, we will invite some of these participants to complete further interviews with the research assistant.

**Who is doing this research?**

Dr Paul Hutton (Associate Professor, Edinburgh Napier University) is the Chief Investigator and Principal Investigator for Scotland. Co-Investigators from Edinburgh Napier University are Professors Thanos Karatzias, Brian Williams and Jill Stavert, and Associate Professor Nadine Dougall. Dr Suzanne O’Rourke is a Co-investigator from the University of Edinburgh. Co-investigators from NHS Lothian are Dr Sean Harper and Dr Andrew Watson. Co-investigators in England are Dr Chris Taylor (Principal Investigator for Pennine Care NHS Foundation Trust), Dr James Kelly (Principal Investigator for Lancashire Care NHS Foundation Trust), Dr Peter Taylor (University of Manchester) and Professor Richard Emsley (King’s College London).

The study is funded by the Chief Scientist Office (Health Improvement, Protection and Services Research Committee – Response Mode Funding Scheme).

**Who is organising the research?**

This study is being organised and sponsored by Edinburgh Napier University. Collaborating institutions and organisations are University of Edinburgh, University of Manchester, King’s College London, NHS Lothian, Pennine Care NHS Foundation Trust and Lancashire Care NHS Foundation Trust.

**Who has reviewed the study?**

The study proposal has been reviewed by the Health Improvement, Protection and Services Research Committee of the Chief Scientist Office, Scotland. A favourable ethical opinion has been obtained from the Scotland A Research Ethics Committee. Edinburgh Napier University and the Research & Development departments of NHS Lothian, Pennine Care NHS Foundation Trust and Lancashire Care NHS Foundation Trust have also reviewed and approved the study.

**How do I refer a patient for this research?**

We encourage referrers to contact us to discuss whether a patient may be eligible. We also encourage referrers to let us know at this stage whether they think their patient currently has capacity to consent to taking part in research.

If a potential participant has capacity to consent, then we will ask their key worker or care coordinator to approach them and provide them with an information leaflet describing the study and what will be asked of them should they wish to participate. Their key worker or care coordinator will be asked to assure them that participation in the study is voluntary and they can change their mind at any time. If the potential participant agrees, then we will contact them directly to arrange to discuss the study further. Please note we will need to consult with the referrer to conduct a risk assessment before meeting any potential participants in person. Potential participants will be given as long as they like to decide to take part, with a minimum period of 48 hours. If the potential participant consents to take part, then we will contact the referrer to complete a referral form.

If a potential participant living in Scotland does not have capacity to consent, then we will ask their key worker to contact their legal representative (i.e., their Guardian or welfare attorney, or their nearest relative). This representative will be asked to give consent on behalf of the person to take part in the study. They will be advised that they are free to decide whether they wish to make this decision or not, and that they are being asked to consider what the person would want, and to set aside their own personal views when making this decision. We will not contact a potential participant or their legal representative until they or their legal representative has informed their key worker that we have permission to do so. If we are given permission to make contact with them, then we will send them and their legal representative information about the study, and offer to discuss it further.

If a potential participant living in England does not have capacity to consent, then we will ask their care coordinator to contact their Nominated or Personal Consultee. This Consultee will be asked for their advice as to whether the person would wish to participate. They will be advised that the researchers will act in accordance with their advice, that they are free to decide whether they wish to offer this advice or not, and that they are being asked to consider what the person would want, and to set aside their own personal views when providing their advice. We will not contact a potential participant or their Consultee until they or their Consultee has informed their care coordinator that we have permission to do so. If we are given permission to make contact with them, then we will send them and their Consultee information about the study, and offer to discuss it further.

If a potential participant living in England or Scotland objects to taking part, or shows distress related to taking part, then we will not include them in the study. If they object or show distress related to participation after they have started taking part, then we will withdraw them from the study.

We can be contacted using the details below:

Dr Paul Hutton (Chief Investigator and Principal Investigator for Scotland) at [p.hutton@napier.ac.uk](mailto:p.hutton@napier.ac.uk) **or 07XXX**;

Dr Chris Taylor (Co-Investigator and Principal Investigator for Pennine Care NHS Foundation Trust) at [chrisdjtaylor@nhs.net](mailto:chrisdjtaylor@nhs.net) **or 07XXX**;

Dr James Kelly (Co-Investigator and Principal Investigator for Lancashire Care NHS Foundation Trust) at [j.a.kelly@lancaster.ac.uk](mailto:j.a.kelly@lancaster.ac.uk) **or 07XXX**;

If you wish to discuss this study with someone who is not involved in the research, please contact:

Dr David Carmichael (NHS Lothian) at [XXXX@XXXX](mailto:david.carmichael1@nhs.net) or **07XXX**

11. Information sheet for Welfare Attorney, Welfare Guardian or nearest relative (Scotland) (version 5)


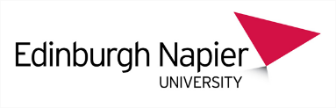


Logo of host NHS organisation to be inserted here


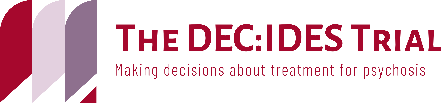


**Information Sheet for Participant Welfare Attorney, Welfare Guardian or Nearest Relative**

**DE**cision-making **C**apacity: **I**ntervention **D**evelopment & **E**valuation in **S**chizophrenia-spectrum disorder: The DEC:IDES Trial

**You have been identified as the Welfare Attorney, Welfare Guardian or Nearest Relative for a person (hereafter ‘P’) who is eligible to take part in our research study. You are being invited to consider giving consent on behalf of P to take part in this study. Before you decide it is important for you to understand why the research is being done and what it will involve. Please take time to read the following information carefully and discuss it with others if you wish. Please ask me if there is anything that is not clear or if you would like more information. Although you are being asked to provide consent on behalf of P, the Adults with Incapacity (Scotland) Act 2000 requires you to put your own views about the research aside and to take into account and consider the present and past wishes and feelings of P, had they been able to consent for themselves. Please let us know of any advance decisions they may have made about participating in research. These should take precedence.**

**If you consent to P taking part we will ask you to read and sign the enclosed consent form. We’ll then give you a copy to keep. We will keep you fully informed during the study so you can let us know if you have any concerns or you think P should be withdrawn.**

**If you decide that P would not wish to take part it will not affect the standard of care they receive in any way.**

**If you are unsure about providing consent on behalf of P you may seek independent advice.**

**We will understand if you do not want to take on this responsibility.**

**The following information is the same as would have been provided to P:**

**Does P have to take part?**

No, it is up to you to decide whether or not P would wish take part. If you do decide P would like to take part you will be given this information sheet to keep and be asked to sign a consent form. If you decide P would wish to take part you are still free to withdraw your consent at any time and without giving a reason. Deciding that P would not wish to take part or withdrawing P from the study will not affect the healthcare that P receives, or his or her’s legal rights.

**What is the purpose of the study?**

Some people hear or see things that others do not, or believe things that others do not. They may be worried that others want to harm them. Sometimes, these experiences and beliefs can lead to the person being diagnosed with a mental health problem such as schizophrenia or psychosis. Sometimes, psychosis can also affect a person’s ability to make their own decisions about treatment – such as taking medication or going into hospital. This means other people, including doctors, may make these decisions instead.

Over the last few years we have been developing new approaches to help people with psychosis make their own decisions about treatment. However, to find out if these approaches are helpful, we need to carry out ‘clinical trials’. Trials are a kind of research study that can compare how helpful different treatment approaches are. However, to produce reliable findings, trials often need to include a lot of people and they need to be very carefully designed.

To ensure these larger trials are well-designed, it is common to run several small trials first. These are known as ‘feasibility’ or ‘pilot’ trials. Although these small trials cannot tell us whether a new approach is effective, they do provide essential information for designing the larger trials.

The aim of our study is therefore to complete several small trials of new approaches to help people with psychosis make their own decisions about treatment. This will help us design larger trials of these new approaches, which will help to ensure they produce reliable results.

**Why has P been asked to take part?**

P has been invited to take part because:

- P’s doctor has given them a diagnosis of schizophrenia or a related psychotic illness.
- P may benefit from having support to make decisions about their treatment
- P is in contact with mental health services in (name of NHS organisation).

Please note further assessment is required to confirm whether P is eligible to take part in the study. The researchers will only know this once they have met with P and asked them some additional questions about their mental health and treatment. We will let you and P know the outcome of this assessment as soon as possible.

**What will happen if I consent to P taking part?**

- A staff member from P’s NHS mental health team will give you this form to read. If you believe P would be interested in taking part, then this staff member will ask you for your permission to share your name, contact details and some information about P’s condition and care with the researcher. This will allow the researcher to begin to assess whether P is eligible to take part, and to contact you directly.
- With your agreement, the researcher will arrange to meet with you, either on the phone or in person. This will allow you to ask the researcher any questions you like about the study. If you decide P would not wish to take part, then the researcher will not contact you or P again.
- If you believe P may wish to take part, then the researcher will contact you no sooner than 2 days after this first meeting, to give you time to think about it and speak to P, before deciding. You can have a longer time if you prefer. If you decide P would wish to take part, then the researcher will ask you to sign a consent form. The researcher will go through this with you in person. They will check you understand everything on the form before you sign it.
- If you consent to P taking part, then the researcher will invite P to a meeting. They will explain the study carefully to them, and provide them with a Participant Information Sheet. They will answer any questions P has and they will assess in more detail whether they are eligible to take part. They will ask P questions about their mental health and the treatment their receive. If this assessment shows P is eligible to take part and if P agrees to doing so, then their involvement in the study will continue and a further meeting will be arranged to begin the research. If P indicates at any point during the study that they do not wish to take part, or if they show any sign of distress related to taking part, then they will be withdrawn. If the assessment shows P is not eligible to take part, the researcher will let you and P know as soon as possible.
- Some participants may regain the capacity to make a decision about whether or not to continue with a study. If this happens to P, we will ask them for their consent to continue with the study, following our process for participants who regain capacity. At all times we will follow the legal requirements of the Adults with Incapacity (Scotland) Act 2000. This means P will always be free to withdraw from the study at any time and without giving a reason.
- If P agrees to taking part, a researcher will meet with them several times over a 24-week period. In each meeting, they will ask P questions about their mental health and the treatment their receive. With your consent and P’s agreement, these meetings will be audio-recorded.
- During this 24-week period, P will be invited to enter 1 of 3 clinical trials, based on the type of difficulties they have.
- In each trial, P will have a 50% chance of receiving either 6 weekly 1-hour sessions of therapy to help them with their decision-making, or 6 weekly 1-hour sessions of more in-depth assessment of what helps or hinders their decision-making. This will be decided randomly. This means neither the researchers, the therapist or P can choose what they will receive. This is important for finding out which approach is most helpful and safe.
- The therapy sessions are designed to help participants with one of the following type of difficulty:
  - Low self-esteem
  - Fears about their diagnosis
  - Gathering information before making decisions
- The assessment sessions are designed to gather more information about what helps or hinders a participant’s decision-making ability. If P is offered this, then the therapist will offer to meet with them after the study is over to discuss the results of this assessment. They will help P understand why they might have difficulties in decision-making, and what could help P with these. With your consent and P’s agreement, we will share this information with P’s clinical team.
- We will also invite some participants to tell the researcher more about their experiences of taking part in the study. Some participants will be invited to tell us more about any improvements they had in their decision-making. These extra meetings should last around 1 hour and will also be audio-recorded. We may quote some of the things participants tell us in any reports we produce, however we will not reveal their name or other information which could identify them.

**What are the possible benefits for P of taking part?**

- If P receives help for self-esteem, fears about their diagnosis or using more information before making decisions, then they may experience improvements in these areas.
- Taking part in this study may also help P understand the factors that help or hinder their ability to make decisions about their treatment. This may help P’s clinical team work out how best to support P in the future.
- The results of this study may also contribute to better mental health care and treatment for people experiencing similar difficulties.

**Who is doing this study?**

Dr Paul Hutton (Associate Professor, Edinburgh Napier University) is leading the overall study. The research team in Scotland includes Professors Thanos Karatzias, Brian Williams and Jill Stavert, and Associate Professor Nadine Dougall, from Edinburgh Napier University, Dr Suzanne O’Rourke from University of Edinburgh, Dr Sean Harper and Dr Andrew Watson from NHS Lothian.

The research team in England includes Dr Chris Taylor (Pennine Care NHS Foundation Trust), Dr James Kelly (Lancashire Care NHS Foundation Trust), Dr Peter Taylor (University of Manchester) and Professor Richard Emsley (King’s College London).

The study is funded by the Chief Scientist Office (Health Improvement, Protection and Services Research Committee – Response Mode Funding Scheme).

**What are the possible disadvantages and risks to P of taking part?**

- The number of assessments P may be asked to take part in ranges from 2 to 3. However P may also be invited to 1 or 2 additional meetings. These assessments and meetings can vary in length. This depends on lots of things, including how P is feeling at the time.
- We will offer breaks every 30 minutes and we will work flexibly according to P’s needs. Efforts will be made to make the meetings as comfortable as possible for P. We will also reimburse P for travel expenses and time, with a £10 Tesco voucher per each of the 2 or 3 assessments. At no point should P feel under pressure to complete the assessments.
- If any aspect of the study causes P distress or they become upset or anxious, this will be communicated to their mental health team team so that they can follow up with P.
- If it appears that P presents a serious risk of self-harm or harm to other people, this will also be communicated and standard NHS procedures would be followed.

**What if there is a problem?**

If you have a concern about any aspect of this study please contact Dr Paul Hutton by phoning 07XXX or emailing [p.hutton@napier.ac.uk](mailto:p.hutton@napier.ac.uk).

If you would like to speak to someone independent from the study, please contact Dr David Carmichael (NHS Lothian) by phoning 07XXX or emailing [XXXX@XXXX](mailto:david.carmichael1@nhs.net)

In the unlikely event that something goes wrong and P is harmed during the research and this is due to someone‘s negligence then P or their representative may have grounds for a legal action for compensation against their NHS organisation but they may have to pay their legal costs. The normal National Health Service complaints mechanisms will still be available to P or their representative (if appropriate).

**What happens when the study is finished?**

For most participants, their involvement in the main part of the study will end around 24 weeks after they joined it. For some participants (those who joined the study later on), their involvement will last around 8 weeks only. Some participants will also be invited to attend additional meetings with the research team.

At the end of the research we will analyse the data from all the participants and write a report. P’s data will be made anonymous as soon as possible and less than three months after their last meeting with the researcher. The anonymous data will be kept for 10 years. We may quote some of the things P tells us in any reports we produce, however we will not reveal P’s name or other information which could identify them.

You and P can choose to have a summary of the results and outcome of the study sent to you once the research has been completed. This information will also be available on our study website ([www.XXX.co.uk](http://www.XXX.co.uk)).

**Will P’s participation in the study be kept confidential?**

All the information we collect during the course of the research will be kept confidential and there are strict laws which safeguard your and P’s privacy at every stage. With your consent we will inform P’s GP that they are taking part.

To ensure that the study is being run correctly, we will ask your consent for responsible representatives from the Sponsor (Edinburgh Napier University) and NHS Institution accessing P’s medical records and data collected during the study, where it is relevant to P taking part in this research. The Sponsor is responsible for overall management of the study and providing insurance and indemnity.

Should information come to light from disclosure during the study suggesting that P, another adult or a child is at risk of harm, standard NHS procedures would be followed to address this risk which may limit confidentiality. Any such disclosure would be handled within NHS policy and would protect confidentiality as best possible.

All identifiable information used at the beginning of the study will be destroyed as soon as possible and replaced with anonymous identifiers. All identifiable information will be kept in NHS sites, before being destroyed.

**What will happen to the results of the study?**

The study will be written up as a scientific journal article. The results will also be presented at conferences. P will not be identifiable in any published results. You and P can choose to have a summary of the results and outcome of the study sent to you once the research has been completed.

**Who is organising the research?**

This study is being organised and sponsored by Edinburgh Napier University.

**Who has reviewed the study?**

The study proposal has been reviewed by the Health Improvement, Protection and Services Research Committee of the Chief Scientist Office, Scotland. A favourable ethical opinion has been obtained from the Scotland A Research Ethics Committee. Edinburgh Napier University and the Research & Development departments of NHS Lothian, Pennine Care NHS Foundation Trust and Lancashire Care NHS Foundation Trust have also reviewed and approved the study.

**If you have any further questions about the study, the research team can be contacted using the details below:**

Dr Paul Hutton (Chief Investigator and Principal Investigator for Scotland) at [p.hutton@napier.ac.uk](mailto:p.hutton@napier.ac.uk) **or 07XXX**;

If you wish to discuss this study with someone who is not involved in the research, please contact:

Dr David Carmichael (NHS Lothian) at [XXXX@XXXX](mailto:david.carmichael1@nhs.net) or **07XXX**

**If you wish to make a complaint about the study please contact NHS Lothian:**

**Patient Experience Team
NHS Lothian
2nd Floor
Waverley Gate
2-4 Waterloo Place
Edinburgh
EH1 3EG
Tel: 0131 536 3370
Email:** [**feedback@nhslothian.scot.nhs.uk**](mailto:feedback@nhslothian.scot.nhs.uk)

Thank you for taking the time to read this information sheet.

12. Information sheet for Consultee (England) (version 4)


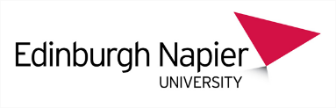


Logo of host NHS organisation to be inserted here


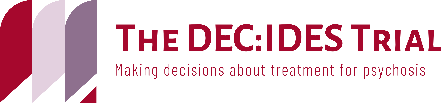


**Consultee Information Sheet**

**DE**cision-making **C**apacity: **I**ntervention **D**evelopment & **E**valuation in **S**chizophrenia-spectrum disorder: The DEC:IDES Trial

**You have been identified as a Consultee for a person (hereafter ‘P’) who is eligible to take part in our research study. We feel P is unable to decide for himself/herself whether to participate in this research. To help decide if P should join the study, we’d like to ask your opinion whether or not they would want to be involved. We’d ask you to consider what you know of P’s wishes and feelings, and to consider their interests.** **Please let us know of any advance decisions they may have made about participating in research. These should take precedence.**

**If you decide P would have no objection to taking part we will ask you to read and sign the enclosed consultee declaration form. We’ll then give you a copy to keep. We will keep you fully informed during the study so you can let us know if you have any concerns or you think P should be withdrawn.**

**If you decide that P would not wish to take part it will not affect the standard of care they receive in any way.**

**If you are unsure about taking the role of consultee you may seek independent advice.**

**We will understand if you do not want to take on this responsibility.**

**The following information is the same as would have been provided to P:**

**Does P have to take part?**

No, we will act in accordance with your advice as to P’s wishes. If you advise that P would like to take part you will be given this information sheet to keep and be asked to sign a consultee declaration form. If you advise that P would wish to take part you are still free to change your advice at any time and without giving a reason. If you advise that P would not wish to take part we will withdraw P from the study. This will not affect the healthcare that P receives, or his or her’s legal rights.

**What is the purpose of the study?**

Some people hear or see things that others do not, or believe things that others do not. They may be worried that others want to harm them. Sometimes, these experiences and beliefs can lead to the person being diagnosed with a mental health problem such as schizophrenia or psychosis. Sometimes, psychosis can also affect a person’s ability to make their own decisions about treatment – such as taking medication or going into hospital. This means other people, including doctors, may make these decisions instead.

Over the last few years we have been developing new approaches to help people with psychosis make their own decisions about treatment. However, to find out if these approaches are helpful, we need to carry out ‘clinical trials’. Trials are a kind of research study that can compare how helpful different treatment approaches are. However, to produce reliable findings, trials often need to include a lot of people and they need to be very carefully designed.

To ensure these larger trials are well-designed, it is common to run several small trials first. These are known as ‘feasibility’ or ‘pilot’ trials. Although these small trials cannot tell us whether a new approach is effective, they do provide essential information for designing the larger trials.

The aim of our study is therefore to complete several small trials of new approaches to help people with psychosis make their own decisions about treatment. This will help us design larger trials of these new approaches, which will help to ensure they produce reliable results.

**Why has P been asked to take part?**

P has been invited to take part because:

- P’s doctor has given them a diagnosis of schizophrenia or a related psychotic illness.
- P may benefit from having support to make decisions about their treatment
- P is in contact with mental health services in (name of NHS organisation).

Please note further assessment is required to confirm whether P is eligible to take part in the study. The researchers will only know this once they have met with P and asked them some additional questions about their mental health and treatment. We will let you and P know the outcome of this assessment as soon as possible.

**What will happen if I advise that P would wish to take part?**

- A staff member from P’s NHS mental health team will give you this form to read. If you believe P would be interested in taking part, then this staff member will ask you for your permission to share your name, contact details and some information about P’s condition and care with the researcher. This will allow the researcher to begin to assess whether P is eligible to take part, and to contact you directly.
- With your agreement, the researcher will arrange to meet with you, either on the phone or in person. This will allow you to ask the researcher any questions you like about the study. If you decide P would not wish to take part, then the researcher will not contact you or P again.
- If you believe P may wish to take part, then the researcher will contact you no sooner than 2 days after this first meeting, to give you time to think about it and speak to P, before providing your advice. You can have a longer time if you prefer. If you decide P would wish to take part, then the researcher will ask you to sign a consultee declaration form. The researcher will go through this with you in person. They will check you understand everything on the form before you sign it.
- If you advise that P would wish to take part, then the researcher will invite P to a meeting. They will explain the study carefully to them, and provide them with a Participant Information Sheet. They will answer any questions P has and they will assess in more detail whether they are eligible to take part. They will ask P questions about their mental health and the treatment their receive. If this assessment shows P is eligible to take part and if P agrees to doing so, then their involvement in the study will continue and a further meeting will be arranged to begin the research. If P indicates at any point during the study that they do not wish to take part, or if they show any sign of distress related to taking part, then they will be withdrawn. If the assessment shows P is not eligible to take part, the researcher will let you and P know as soon as possible.
- Some participants may regain the capacity to make a decision about whether or not to continue with a study. If this happens to P, we will ask them for their consent to continue with the study, following our process for participants who regain capacity. At all times we will follow the legal requirements of the Mental Capacity (England and Wales) Act 2005. This means P will always be free to withdraw from the study at any time and without giving a reason.
- If P agrees to taking part, a researcher will meet with them several times over a 24-week period. In each meeting, they will ask P questions about their mental health and the treatment their receive. With your and P’s agreement, these meetings will be audio-recorded.
- During this 24-week period, P will be invited to enter 1 of 3 clinical trials, based on the type of difficulties they have.
- In each trial, P will have a 50% chance of receiving either 6 weekly 1-hour sessions of therapy to help them with their decision-making, or 6 weekly 1-hour sessions of more in-depth assessment of what helps or hinders their decision-making. This will be decided randomly. This means neither the researchers, the therapist or P can choose what they will receive. This is important for finding out which approach is most helpful and safe.
- The therapy sessions are designed to help participants with one of the following type of difficulty:
  - Low self-esteem
  - Fears about their diagnosis
  - Gathering information before making decisions
- The assessment sessions are designed to gather more information about what helps or hinders a participant’s decision-making ability. If P is offered this, then the therapist will offer to meet with them after the study is over to discuss the results of this assessment. They will help P understand why they might have difficulties in decision-making, and what could help P with these. With your and P’s agreement, we will share this information with P’s clinical team.
- We will also invite some participants to tell the researcher more about their experiences of taking part in the study. Some participants will be invited to tell us more about any improvements they had in their decision-making. These extra meetings should last around 1 hour and will also be audio-recorded. We may quote some of the things participants tell us in any reports we produce, however we will not reveal their name or other information which could identify them.

**What are the possible benefits for P of taking part?**

- If P receives help for self-esteem, fears about their diagnosis or using more information before making decisions, then they may experience improvements in these areas.
- Taking part in this study may also help P understand the factors that help or hinder their ability to make decisions about their treatment. This may help P’s clinical team work out how best to support P in the future.
- The results of this study may also contribute to better mental health care and treatment for people experiencing similar difficulties.

**Who is doing this study?**

Dr Paul Hutton (Associate Professor, Edinburgh Napier University) is leading the overall study. The research team in Scotland includes Professors Thanos Karatzias, Brian Williams and Jill Stavert, and Associate Professor Nadine Dougall, from Edinburgh Napier University, Dr Suzanne O’Rourke from University of Edinburgh, Dr Sean Harper and Dr Andrew Watson from NHS Lothian.

The research team in England includes Dr Chris Taylor (Pennine Care NHS Foundation Trust), Dr James Kelly (Lancashire Care NHS Foundation Trust), Dr Peter Taylor (University of Manchester) and Professor Richard Emsley (King’s College London).

The study is funded by the Chief Scientist Office (Health Improvement, Protection and Services Research Committee – Response Mode Funding Scheme).

**What are the possible disadvantages and risks of taking part?**

- The number of assessments P may be asked to take part in ranges from 2 to 3. However P may also be invited to 1 or 2 additional meetings. These assessments and meetings can vary in length. This depends on lots of things, including how P is feeling at the time.
- We will offer breaks every 30 minutes and we will work flexibly according to P’s needs. Efforts will be made to make the meetings as comfortable as possible for P. We will also reimburse P for travel expenses and time, with a £10 Tesco voucher per each of the 2 or 3 assessments. At no point should P feel under pressure to complete the assessments.
- If any aspect of the study causes P distress or they become upset or anxious, this will be communicated to their mental health team team so that they can follow up with P.
- If it appears that P presents a serious risk of self-harm or harm to other people, this will also be communicated and standard NHS procedures would be followed.

**What if there is a problem?**

If you have a concern about any aspect of this study please contact Dr Paul Hutton by phoning 07XXX or emailing [p.hutton@napier.ac.uk](mailto:p.hutton@napier.ac.uk).

If you would like to speak to someone independent from the study, please contact Dr David Carmichael (NHS Lothian) by phoning 07XXX or emailing [XXXX@XXXX](mailto:david.carmichael1@nhs.net)

In the unlikely event that something goes wrong and P is harmed during the research and this is due to someone‘s negligence then P may have grounds for a legal action for compensation against their NHS organisation but they may have to pay their legal costs. The normal National Health Service complaints mechanisms will still be available to you or P (if appropriate).Some participants may regain the capacity to make a decision about whether or not to continue with a study. If this happens to P, we will ask them for their consent to continue with the study, following our process for new participants who have capacity. At all times we will follow the legal requirements of the Mental Capacity (England and Wales) Act 2005. This means P will always be free to withdraw from the study at any time and without giving a reason.

**What happens when the study is finished?**

For most participants, their involvement in the main part of the study will end around 24 weeks after they joined it. For some participants (those who joined the study later on), their involvement will last around 8 weeks only. Some participants will also be invited to attend additional meetings with the research team.

At the end of the research we will analyse the data from all the participants and write a report. P’s data will be made anonymous as soon as possible and less than three months after their last meeting with the researcher. The anonymous data will be kept for 10 years. We may quote some of the things P tells us in any reports we produce, however we will not reveal P’s name or other information which could identify them.

You and P can choose to have a summary of the results and outcome of the study sent to you once the research has been completed. This information will also be available on our study website ([www.XXX.co.uk](http://www.XXX.co.uk)).

**Will P’s participation in the study be kept confidential?**

All the information we collect during the course of the research will be kept confidential and there are strict laws which safeguard your and P’s privacy at every stage. We will inform P’s GP that they are taking part, but only if you advise that P would agree to this.

To ensure that the study is being run correctly, we will ask you whether P would agree to responsible representatives from the Sponsor (Edinburgh Napier University) and NHS Institution accessing P’s medical records and data collected during the study, where it is relevant to P taking part in this research. The Sponsor is responsible for overall management of the study and providing insurance and indemnity.

Should information come to light from disclosure during the study suggesting that P, another adult or a child is at risk of harm, standard NHS procedures would be followed to address this risk which may limit confidentiality. Any such disclosure would be handled within NHS policy and would protect confidentiality as best possible.

All identifiable information used at the beginning of the study will be destroyed as soon as possible and replaced with anonymous identifiers. All identifiable information will be kept in NHS sites, before being destroyed.

**What will happen to the results of the study?**

The study will be written up as a scientific journal article. The results will also be presented at conferences. P will not be identifiable in any published results. You and P can choose to have a summary of the results and outcome of the study sent to you once the research has been completed.

**Who is organising the research?**

This study is being organised and sponsored by Edinburgh Napier University.

**Who has reviewed the study?**

The study proposal has been reviewed by the Health Improvement, Protection and Services Research Committee of the Chief Scientist Office, Scotland. A favourable ethical opinion has been obtained from the Scotland A Research Ethics Committee. Edinburgh Napier University and the Research & Development departments of NHS Lothian, Pennine Care NHS Foundation Trust and Lancashire Care NHS Foundation Trust have also reviewed and approved the study.

**If you have any further questions about the study, the research team can be contacted using the details below:**

Dr Paul Hutton (Chief Investigator and Principal Investigator for Scotland) at [p.hutton@napier.ac.uk](mailto:p.hutton@napier.ac.uk) **or 07XXX**;

Dr Chris Taylor (Co-Investigator and Principal Investigator for Pennine Care NHS Foundation Trust) at [chrisdjtaylor@nhs.net](mailto:chrisdjtaylor@nhs.net) **or 07XXX**;

Dr James Kelly (Co-Investigator and Principal Investigator for Lancashire Care NHS Foundation Trust) at [j.a.kelly@lancaster.ac.uk](mailto:j.a.kelly@lancaster.ac.uk) **or 07XXX**;

If you wish to discuss this study with someone who is not involved in the research, please contact:

Dr David Carmichael (NHS Lothian) at [XXXX@XXXX](mailto:david.carmichael1@nhs.net) or **07XXX**

**If you wish to make a complaint about the study and P is receiving care from Pennine Care NHS Foundation Trust, please contact:**

**Patient Advice and Liaison Services (PALS)**

**Pennine Care NHS Foundation Trust**

**Trust Headquarters**

**225 Old Street**

**Ashton-under-Lyne**

**Lancashire**

**OL6 7SR**

**Tel: 0161 716 3178**

**Online**: <https://www.penninecare.nhs.uk/contact/?id=1218>

**If you wish to make a complaint about the study and P is receiving care from Lancashire Care NHS Foundation Trust, please contact:**

**Hearing Feedback Team**

**Lancashire Care NHS Foundation Trust**

**Sceptre Point**

**Sceptre Way**

**Walton Summit**

**Bamber Bridge**

**Preston**

**PR5 6AW**

**Tel: 01772 695315**

**Email:** [**hearing.feedback@lancashirecare.nhs.uk**](mailto:hearing.feedback@lancashirecare.nhs.uk)

Thank you for taking the time to read this information sheet.

13. Consent form for Welfare Attorney, Welfare Guardian or nearest relative (Scotland) (version 2)


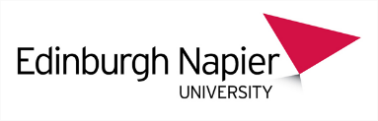


Logo of host NHS organisation to be inserted here


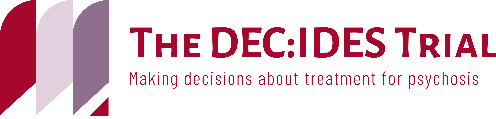


**Welfare Attorney, Welfare Guardian or Nearest Relative**

**Consent Form**

**Participant Identification Number**:

**Title of Project:** DEcision-making Capacity: Intervention Development & Evaluation in Schizophrenia-spectrum disorder: The DEC:IDES Trial

**Please initial box**

| I confirm that I have read and understand the information sheet for the above study and have had the opportunity to consider the information and ask questions. |
| --- |
|  |
| I understand that [participant name]’s participation is voluntary and that I am free to withdraw them from the study at any time, without giving any reason, without their medical care or legal rights being affected |
|  |
| I understand that relevant sections of [participant name]’s medical notes and data collected during the study may be looked at by the researchers, individuals from the Edinburgh Napier University (Study Sponsor), from [name of NHS organisation] or other authorities, where it is relevant to them taking part in this research. I give permission for these individuals to have access to [participant name]’s data. |
|  |
| I agree to their General Practitioner or other care professional being informed of their participation in the study. |
|  |
| I agree that audio recordings may be made of parts of [participant name]’s sessions and assessments to help monitor the project and to analyse the data. I understand that this may involve some of their quotes from the audio recordings being used for the write-up up of a report which could be published. I understand [participant name]’s name or other information which could identify them will not be revealed *(optional).* |
|  |
| I agree that if [participant name] withdraws from the study the researchers can retain any data [participant name] has provided up until that point, unless I or [participant name] inform the researchers we would like it to be withdrawn. |
|  |
| I give my consent for [participant name] to take part in the above study. |

**Name of Researcher:**

| I confirm that I am the nearest relative for ______________________________ and that no other nearest relative or welfare attorney or guardian exists | | | | | | |
| --- | --- | --- | --- | --- | --- | --- |
|  | | | | | | |
| Relationship to participant: |  |  | |  |  |  |
|  |  |  | |  |  |  |
| I confirm that I am the Welfare Attorney or Guardian for: | | |  | | |  |
|  | | |  |  |  |  |
| Name of person giving consent: |  | Date: | |  | Signature: |  |
|  |  |  | |  |  |  |
| Name of person taking consent: |  | Date: | |  | Signature |  |

When completed: 1 (original) to be kept in care record, 1 for Guardian, Welfare Attorney or Nearest Relative; 1 for researcher site file.

14. Consultee Declaration Form (England) (version 1)


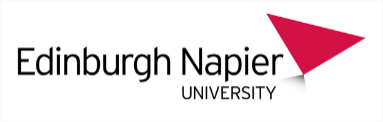


Logo of host NHS organisation to be inserted here


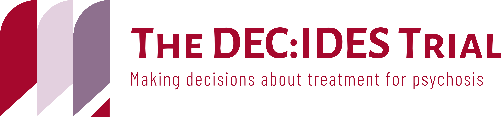


**Consultee Declaration Form**

**Participant Identification Number**:

**Title of Project:** DEcision-making Capacity: Intervention Development & Evaluation in Schizophrenia-spectrum disorder: The DEC:IDES Trial

**Name of Researcher:**

**Please initial box**

| I [name of consultee] have been consulted about [name of potential participant]’s  participation in this research project. I have had the opportunity to ask questions  about the study and understand what is involved. |
| --- |
|  |
| In my opinion he/she would have no objection to taking part in the above study. |
|  |
| I understand that I can request he/she is withdrawn from the study at any time, without giving any reason and without his/her care or legal rights being affected. |
|  |
| I understand that relevant sections of his/her care record and data collected during the study may be looked at by responsible individuals from the study Sponsor (Edinburgh Napier University) and [name of NHS organisation] or from regulatory authorities, where it is relevant to their taking part in this research. |
|  |
| I agree to their GP or other care professional being informed of their participation in the study |
|  |
| I agree that if [participant name] withdraws from the study the researchers can retain any data [participant name] has provided up until that point, unless I or [participant name] inform the researchers we would like it to be withdrawn. |
|  |
| I agree that audio recordings may be made of parts of [participant name]’s sessions and assessments to help monitor the project and to analyse the data. I understand that this may involve some of their quotes from the audio recordings being used for the write-up up of a report which could be published. I understand their name or other information which could identify them will not be revealed *(optional).* |

.

| Name of consultee: |  | | Date: |  | Signature: |  |
| --- | --- | --- | --- | --- | --- | --- |
|  |  | |  |  |  |  |
| Relationship to participant: | |  |  |  |  |  |
|  | |  |  |  |  |  |
| Person undertaking consultation (if different to researcher): |  | | Date: |  | Signature: |  |
|  |  | |  |  |  |  |
| Researcher: |  | | Date: |  | Signature |  |

When completed: 1 (original) to be kept in care record, 1 for consultee; 1 for researcher site file.

15. Information sheet for clinicians participating in qualitative study (version 1)


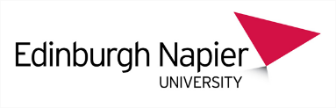


Logo of host NHS organisation to be inserted here


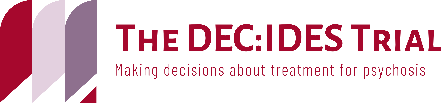


**Participant Information Sheet**

**DE**cision-making **C**apacity: **I**ntervention **D**evelopment & **E**valuation in **S**chizophrenia-spectrum disorder: The DEC:IDES Trial

**You are being invited to take part in a research study. Before you decide whether or not to take part, it is important for you to understand why the research is being done and what it will involve. Please take time to read the following information carefully. Talk to others about the study if you wish. Contact us if there is anything that is not clear or if you would like more information. Take time to decide whether or not you wish to take part.**

**What is the purpose of the study?**

Treatment decision-making capacity (‘capacity’) refers to a person’s ability to make decisions about their treatment. It is an important issue for people diagnosed with a schizophrenia-spectrum disorder (‘psychosis’) because impaired capacity can mean a person does not understand what treatment options are available, or the implications of those options.

In 2018 the National Institute of Health & Care Excellence (NICE) called for clinical trials of interventions such as talking therapies to help people regain capacity. However, running these trials can take several years. One way of reducing this delay is to run several trials at the same time, as part of one bigger trial. This bigger type of trial is also called an ‘Umbrella’ trial. Although Umbrella trials have been used to accelerate the development of physical health interventions, they have yet to be used in mental health.

For these reasons, we are running a study to find out whether people with psychosis will take part in an Umbrella trial of talking therapies to improve their treatment decision-making capacity (the DEC:IDES trial). We want to understand their experiences of participation, and we also want to understand the experiences of clinicians who have referred their patients to this study.

**Do I have to take part?**

No, it is up to you to decide whether or not to take part. If you do decide to take part you will be given this information sheet to keep and be asked to sign a consent form. If you decide to take part you are still free to withdraw at any time and without giving a reason. Deciding not to take part or withdrawing from the study will not affect the healthcare that you receive, or your legal rights.

**Why have I been asked to take part?**

You have been invited to take part because:

- You are a health or social care professional working in NHS mental health services
- You have referred a participant to the DEC:IDES trial and/or
- You have provided clinical or social care support to a DEC:IDES participant while they have taken part

**What will happen if I take part?**

A researcher will give you this form to read. If you are interested in taking part, then the researcher will arrange to meet with you, either on the phone or in person. This will allow you to ask the researcher any questions you like about the study. If you decide you don’t want to take part, then the researcher will not contact you again. If you remain interested in taking part, then the researcher will contact you no sooner than 2 days after this first meeting, to give you time to think about it before deciding. You can have a longer time if you prefer. If you decide to take part, then the researcher will then meet with you to sign a consent form. The researcher will go through this with you in person. They will check you understand everything on the form before you sign it.

If you consent to taking part, then the researcher will ask you a number of questions about your experience of the DEC:IDES trial, and ask you to provide some information about your professional background and training. This meeting will last around 1 hour. To understand your responses, they will be audio-recorded and transcribed. They may contact you again to seek your view on the issues that appear to be emerging from interviews with you and other participants. This follow-up meeting will last between 30 minutes and 1 hour.

**What are the possible benefits of taking part?**

The results will help us understand what health and social care staff view as helpful about the DEC:IDES trial, and what we need to change. This will be important for designing and running a larger version of this trial, in turn helping us to improve the mental health care and treatment for people experiencing psychosis.

**Who is doing this study?**

Dr Paul Hutton (Associate Professor, Edinburgh Napier University) is leading the overall study. The research team in Scotland includes Professors Thanos Karatzias, Brian Williams and Jill Stavert, and Associate Professor Nadine Dougall, from Edinburgh Napier University, Dr Suzanne O’Rourke from University of Edinburgh, Dr Sean Harper and Dr Andrew Watson from NHS Lothian.

The research team in England includes Dr Chris Taylor (Pennine Care NHS Foundation Trust), Dr James Kelly (Lancashire Care NHS Foundation Trust), Dr Peter Taylor (University of Manchester) and Professor Richard Emsley (King’s College London).

The study is funded by the Chief Scientist Office (Health Improvement, Protection and Services Research Committee – Response Mode Funding Scheme).

**What are the possible disadvantages and risks of taking part?**

We do not foresee any risks to your taking part in this part of the research. The meetings will take up to two hours in total, but we will work flexibly according to your needs. Efforts will be made to make the meetings as comfortable as possible. At no point should you feel under pressure to take part.

**What if there is a problem?**

If you live in Scotland and have a concern about any aspect of this study please contact Dr Paul Hutton by phoning 07XXX or emailing p.hutton@napier.ac.uk.

If you live in England and are employed by Pennine Care NHS Foundation Trust, you can contact Dr Chris Taylor by phoning 07XXX or emailing [chrisdjtaylor@nhs.net](mailto:chrisdjtaylor@nhs.net).

If you live in England and are employed by Lancashire Care NHS Foundation Trust, you can contact Dr James Kelly by phoning 07XXX or emailing [j.a.kelly@lancaster.ac.uk](mailto:j.a.kelly@lancaster.ac.uk)

They will do their best to answer your questions.

If you would like to speak to someone independent from the study, please contact Dr David Carmichael (NHS Lothian) by phoning 07XXX or emailing [XXX@XXXX](mailto:david.carmichael1@nhs.XXX@XXXnet)

In the unlikely event that something goes wrong and you are harmed during the research and this is due to someone‘s negligence then you may have grounds for a legal action for compensation against your NHS organisation but you may have to pay your legal costs. The normal National Health Service complaints mechanisms will still be available to you (if appropriate).

**What happens when the study is finished?**

At the end of the research we will analyse the data from all the participants and write a report. Your data will be made anonymous as soon as possible and less than three months after the meeting. The anonymous data will be kept for 10 years. We may quote some of the things you tell us in any reports we produce, however we will not reveal your name or other information which could identify you.

You can choose to have a summary of the results and outcome of the study sent to you once the research has been completed. This information will also be available on our study website ([www.XXX.co.uk](http://www.XXX.co.uk)).

**Will my taking part in the study be kept confidential?**

All the information we collect during the course of the research will be kept confidential and there are strict laws which safeguard your privacy at every stage.

To ensure that the study is being run correctly, we will ask your consent for responsible representatives from the Sponsor (Edinburgh Napier University) and [name of NHS organisation] to access your data collected during the study, where it is relevant to you taking part in this research. The Sponsor is responsible for overall management of the study and providing insurance and indemnity.

Should information come to light from disclosure during the study suggesting that you, another adult or a child is at risk or harm, standard NHS procedures would be followed to address this risk which may limit confidentiality. Any such disclosure would be handled within NHS policy and would protect confidentiality as best possible.

All identifiable information used at the beginning of the study will be destroyed as soon as possible and replaced with anonymous identifiers. All identifiable information will be kept in NHS sites, before being destroyed.

**What will happen to the results of the study?**

The study will be written up as a scientific journal article. The results will also be presented at conferences. You will not be identifiable in any published results. You can choose to have a summary of the results and outcome of the study sent to you once the research has been completed.

**Who is organising the research?**

This study is being organised and sponsored by Edinburgh Napier University.

**Who has reviewed the study?**

The study proposal has been reviewed by the Health Improvement, Protection and Services Research Committee of the Chief Scientist Office, Scotland. A favourable ethical opinion has been obtained from the Scotland A Research Ethics Committee. Edinburgh Napier University and the Research & Development departments of NHS Lothian, Pennine Care NHS Foundation Trust and Lancashire Care NHS Foundation Trust have also reviewed and approved the study.

**If you have any further questions about the study, the research team can be contacted using the details below:**

Dr Paul Hutton (Chief Investigator and Principal Investigator for Scotland) at [p.hutton@napier.ac.uk](mailto:p.hutton@napier.ac.uk) **or 07XXX**;

Dr Chris Taylor (Co-Investigator and Principal Investigator for Pennine Care NHS Foundation Trust) at [chrisdjtaylor@nhs.net](mailto:chrisdjtaylor@nhs.net) **or 07XXX**;

Dr James Kelly (Co-Investigator and Principal Investigator for Lancashire Care NHS Foundation Trust) at [j.a.kelly@lancaster.ac.uk](mailto:j.a.kelly@lancaster.ac.uk) **or 07XXX**;

If you wish to discuss this study with someone who is not involved in the research, please contact:

Dr David Carmichael (NHS Lothian) at [XXXX@XXXX](mailto:david.carmichael1@nhs.net) or **07XXX**

**If you wish to make a complaint about the study please contact the Complaints Department of Edinburgh Napier University:**

**University complaints office**

**Appeals, complaints and conduct**

**Sighthill Campus**

**Edinburgh**

**EH11 4DE**

**Tel: 0131 455 2396
Email:** [complaints@napier.ac.uk](mailto:complaints@napier.ac.uk)

Thank you for taking the time to read this information sheet.

16. Participant consent form (version 1)


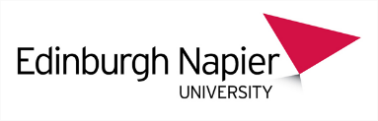


Logo of host NHS organisation to be inserted here


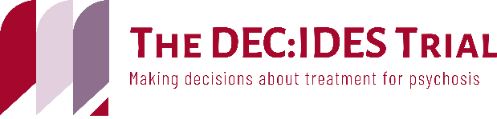


**Participant Consent Form**

**Participant Identification Number**:

**Title of Project:** DEcision-making Capacity: Intervention Development & Evaluation in Schizophrenia-spectrum disorder: The DEC:IDES Trial

**Please initial box**

**Name of Researcher:**

| I confirm that I have read and understand the information sheet for the above study and have had the opportunity to consider the information and ask questions. |
| --- |
|  |
| I understand that my participation is voluntary and that I am free to withdraw from the study at any time, without giving any reason, without my medical care or legal rights being affected |
|  |
| I understand that relevant sections of my medical notes and data collected during the study may be looked at by the researchers, individuals from the Edinburgh Napier University (Study Sponsor), from [name of NHS organisation] or other authorities, where it is relevant to my taking part in this research. I give permission for these individuals to have access to my data. |
|  |
| I agree to my General Practitioner or other care professional being informed of my participation in the study. |
|  |
| I agree that audio recordings may be made of parts of my sessions and assessments to help monitor the project and to analyse the data. I understand that this may involve some of my quotes from the audio recordings being used for the write-up up of a report which could be published. I understand my name or other information which could identify me will not be revealed *(optional).* |
|  |
| I agree that if I withdraw from the study the researchers can retain any data I have provided up until that point, unless I inform the researchers I would like it to be withdrawn. |
|  |
| I agree to take part in the above study. |

.

| Name of participant: |  | Date: |  | Signature: |  |
| --- | --- | --- | --- | --- | --- |
|  |  |  |  |  |  |
| Name of person taking consent: |  | Date: |  | Signature |  |

When completed: 1 (original) to be kept in care record, 1 for Participant; 1 for researcher site file.

17. Consent form for clinicians participating in qualitative study (version 1)


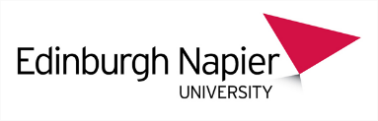


Logo of host NHS organisation to be inserted here


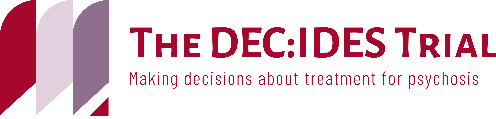


**Participant Consent Form**

**Participant Identification Number**:

**Title of Project:** DEcision-making Capacity: Intervention Development & Evaluation in Schizophrenia-spectrum disorder: The DEC:IDES Trial

**Please initial box**

**Name of Researcher:**

| I confirm that I have read and understand the information sheet for the above study and have had the opportunity to consider the information and ask questions. |
| --- |
|  |
| I understand that my participation is voluntary and that I am free to withdraw from the study at any time, without giving any reason, without my medical care or legal rights being affected |
|  |
| I understand that data collected during the study may be looked at by the researchers, individuals from the Edinburgh Napier University (Study Sponsor), from [name of NHS organisation] or other authorities, where it is relevant to my taking part in this research. I give permission for these individuals to have access to my data. |
|  |
| I agree that audio recordings may be made of parts of my sessions and assessments to help monitor the project and to analyse the data. I understand that this may involve some of my quotes from the audio recordings being used for the write-up up of a report which could be published. I understand my name or other information which could identify me will not be revealed. |
|  |
| I agree that if I withdraw from the study the researchers can retain any data I have provided up until that point, unless I inform the researchers I would like it to be withdrawn. |
|  |
| I agree to take part in the above study. |

.

| Name of participant: |  | Date: |  | Signature: |  |
| --- | --- | --- | --- | --- | --- |
|  |  |  |  |  |  |
| Name of person taking consent: |  | Date: |  | Signature |  |

When completed: 1 (original) to be kept in care record, 1 for Participant; 1 for researcher site file.

1. We were advised on 8^th^ September 2022 by the Scottish Research Ethics Committee (REC) that duplicate amendments to the Scottish and English RECs were not required if the amendment did not involve changes that pertain to documentation relevant to adults lacking capacity to consent to participation. No subsequent amendments involved changes to this documentation, so were submitted only to the English REC for approval. [↑](#footnote-ref-1)
